# Delineating molecular mechanisms on the acquisition of *in vitro-*adapted colistin-resistant *Klebsiella pneumoniae* by transcriptomic analysis

**DOI:** 10.1128/spectrum.03428-24

**Published:** 2025-10-21

**Authors:** Su Min Kyung, Jun Ho Lee, Eun-Seo Lee, Xi-Rui Xiang, Han Sang Yoo

**Affiliations:** 1 Department of Infectious Disease, College of Veterinary Medicine, Seoul National University 65505, Seoul, Republic of Korea; Emory University School of Medicine, Atlanta, Georgia, USA

**Keywords:** colistin, multidrug resistance, NDM, *Klebsiella pneumoniae*, RNA-seq, quorum-sensing

## Abstract

The worldwide dissemination of colistin-resistant pathogens, despite global efforts, is a serious public health concern, considering that colistin is regarded as a last-resort antimicrobial agent for the clinical treatment of multidrug-resistant (MDR) Gram-negative bacterial infections. Since the development of novel strategies and technologies, such as limiting bacterial adaptation, is essential for our society, adaptive laboratory evolution (ALE) was conducted under colistin pressure to uncover poorly understood mechanisms of colistin adaptation and provide a fundamental basis for future technologies. In this study, two isolates of *Klebsiella pneumoniae* were subjected to ALE under colistin pressure, with each isolate serving as the ancestor of three mutant strains resulting from independently conducted ALE experiments. Consequently, all strains surpassed the resistance threshold, and the resistant ratio of the bacterial population exceeded 60% by at least the seventh day of colistin pressure. Whole-genome resequencing revealed multiple gene variants potentially associated with colistin adaptation, warranting further assessment. The RNA-seq results from each stage of pressure revealed that in the early stage, pathways related to quorum sensing are involved, while in the later stage, pathways related to colistin target modification mechanisms were activated. This suggests that different reactions and pathways contribute to colistin survival at the early and later stages. The findings presented in this study will contribute to a deeper understanding of colistin adaptation in *K. pneumoniae* and provide valuable insights for further studies aimed at establishing new strategies to prevent the further emergence of colistin-resistant *K. pneumoniae*.

**IMPORTANCE:**

Nowadays, only a few treatment options remain for widespread multidrug-resistant (MDR) Gram-negative bacterial infections, including the old antibiotic colistin. However, colistin-resistant clinical pathogens are spreading globally, further limiting treatment options. The future does not look promising due to the increasing global use of antibiotics and the uncontrolled spread of MDR pathogens. Moreover, the development of novel antibiotics has been limited in the industrial sector due to the rapid adaptation of the clinical pathogens. Therefore, the hope relies solely on the development of novel strategies and technologies to manage bacterial infections or limit bacterial adaptations. In this study, a new strategy has been proposed based on findings from *in vitro* colistin pressure to limit bacterial adaptation to colistin. Given the growing future concerns, the novel perspectives proposed in this study could provide a fundamental basis for developing novel materials or measures to limit antimicrobial adaptation in clinical environments.

## INTRODUCTION

*Klebsiella pneumoniae* is one of the most threatening bacterial strains in the nosocomial environment due to its ability to accumulate antibiotic resistance genes in genes and adapt to new environments (1, 2). The capability of the *Klebsiella* species to gain multidrug resistance (MDR) is notorious. However, the speed of the evolution of the strain is more rapid than the invention of novel antibiotics.

Polymyxins (polymyxin E, or colistin and polymyxin B) are recognized as last-resort antimicrobial agents for treating MDR infections. Polymyxins are known to destroy bacterial cell membranes by binding the drug compound to phospholipids within the microbial cell membrane (3). Following its initial introduction in 1952, colistin was used for the treatment of Gram-negative bacterial infections until the early days of the 1980s (4). However, colistin was excluded from clinical use due to unwelcome side effects, such as nephrotoxicity, following the development of second- and third-generation cephalosporins (5, 6). Nevertheless, the rise of multidrug-resistant Gram-negative bacterial infections prompted the reintroduction of colistin due to its wide range of action against these strains. Colistin resistance is known to occur due to alterations in the bacterial lipopolysaccharide (LPS) membrane and the overexpression of efflux pumps. In particular, chromosomal point mutations in genes, such as *mgrB*, *pmrAB*, and *phoPQ,* lead to changes in the expression of two-component systems, resulting in the addition of 4-amino-4-deoxy-L-arabinose (L-Ara4N) or phosphoethanolamine (pEtN) to lipid A (7, 8). These modifications reduce the negative charge of LPS and interfere with colistin binding. Similarly, the presence of several *mcr* genes encoding a pEtN transferase leads to the addition of pEtN to the lipid A moiety of LPS, thereby reducing its negative charge of LPS (9, 10). Overexpression of efflux pumps, caused by point mutations in genes, such as *acrAB*, *ramA*, or *ramR,* can also lead to colistin resistance (11). Given the ability of *Klebsiella* species to rapidly adapt to colistin through the horizontal transfer of resistance genes (12), multiple strategies, along with novel approaches, should be implemented to address the emergence of additional MDR *Klebsiella* strains.

One of the promising strategies involves applying evolutionary biology to understand evolutionary mechanics and identifying methods to slow down or block the evolution of strains developing antimicrobial resistance (AMR) (13). Adaptive laboratory evolution (ALE) is an experimental guiding cellular evolution in the laboratory to enhance fitness to specific environmental conditions, potentially leading to the selection of species with beneficial mutations or recombination (14). Since the initial report of ALE in the 1950s by Novick and Szliard (15), various iterations of ALE experiments have been documented, leading to further understanding of various aspects of evolution (16, 17). One ongoing long-term *Escherichia coli* ALE experiment, conducted by Lenski et al. (18), was initiated in 1988 and has reached nearly 80,000 generations to date. Studying ALE allows for insights into species adaptation processes and the development of microbial cell factories for bioproduction. The ALE experiment is a widely used technique in the bacterial industry.

Recently, it has been increasingly noticed as a useful technique to gain a deeper understanding of the underlying mechanisms of bacterial evolution and biological phenomena. In altered environments, bacterial strains are known to respond to changes through either temporary or permanent adaptations. Long-term and intense environmental changes, such as antibiotic pressure, are known to induce both temporary and permanent adaptations (13, 19, 20).

In this study, ALE was performed to uncover previously unknown evolutionary processes that take place during the development of colistin resistance in *K. pneumoniae*. To reveal differentially expressed RNAs during each stage of resistance development, serial RNA-seq was conducted. The genomic variations in the adapted strains were identified using whole-genome resequencing.

## RESULTS

### Increased MIC values and resistant strains as a result of the ALE

**Fig 1:**
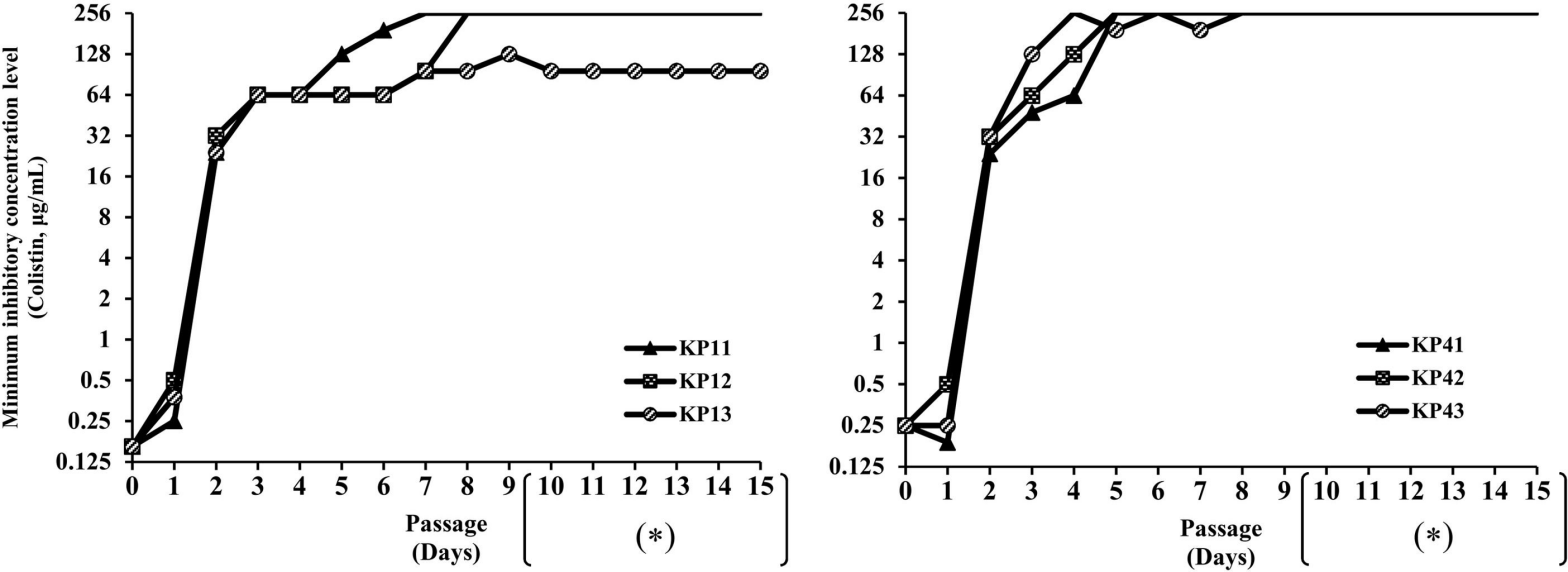
The MIC values against colistin throughout the antibiotic pressure passages. The MIC values were determined each day after the serial passages of the antibiotic pressure. The MIC values determined during cultures in fresh medium were marked with an asterisk (*). KP11, KP12, and KP13 all originated from KP1 and represent biological replicates of the ALE adaptation procedure. Similarly, KP41, KP42, and KP43 all originated from KP4 as biological replicates.

The ALE pressure was applied to the two strains for 9 days, resulting in the generation of three adapted strains from each ancestor (KP1 and KP4). KP11, KP12, and KP13 were derived from the same ancestor, KP1, through independent experiments, representing three biological replicates. Similarly, KP41, KP42, and KP43 were derived from KP4. At the start of the experiment, the minimum inhibitory concentration (MIC) value for KP1 was 0.1625 µg/mL, and for KP4, it was 0.25 µg/mL. As a result of the ALE experiments, all six strains exhibited resistance (identified by MIC values exceeding 4 µg/mL) after the third day of exposure to antibiotic pressure (Fig. 1). On the ninth day of exposure to pressure, MIC values for five out of the six strains exceeded 256 µg/mL, surpassing the limits of our MIC measurement. The MIC values remained consistent for all adapted strains after 6 days of culturing without colistin pressure.

**Fig 2:**
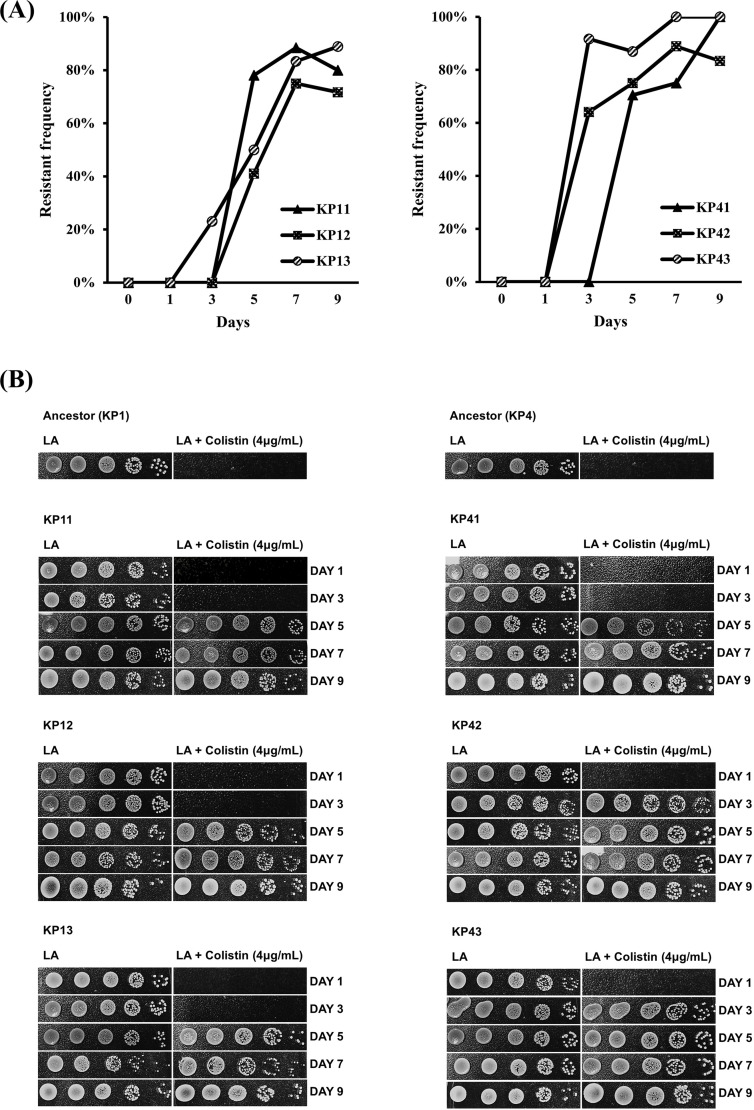
The frequency of resistance is increasing throughout the antibiotic pressure passages. The ratios of resistant strains were determined by dividing the count of resistant bacteria by the total bacterial count on colistin-supplemented (4 µg/mL) LA plates. The ratio of resistance was confirmed to increase (**A**) in colony formation test conducted on days 0, 1, 3, 5, 7, and 9 (**B**), which aimed to identify the resistant colony-forming unit (CFU) at each stage of the antibiotic pressure passages. KP11, KP12, and KP13 all originated from KP1 and represent biological replicates of the ALE adaptation procedure. Similarly, KP41, KP42, and KP43 all originated from KP4 as biological replicates.

The frequencies of resistant strains were confirmed to increase during passages of the antibiotic pressure (Fig. 2). The ratio of resistant colonies was confirmed to exceed 70% on the fifth day of antibiotic pressure in four strains (KP11, KP41, KP42, and KP43). The ratio of resistant colonies in two strains (KP41 and KP43) was confirmed to exceed 100% at the endpoint of the antibiotic passage. A significant fitness cost was unobservable in association with adaptation and increased antibiotic resistance compared to the ancestor strain, as determined by growth rate analysis (Fig. S1).

### The general differentiated expressed genes in strains

The general summary regarding the RNA-seq result is presented in Table S1. The mapped rate (%) of the subjects using the *K. pneumoniae* KP-1 gene (NCBI reference sequence NZ_CP012883.1) as a reference ranged from 79.75 to 89.74. The general summary of differentially expressed genes (DEGs) and the significantly differentiated genes (false discovery rate [FDR] < 0.05) is presented in Table S2. Differentially expressed genes of all subjected strains were analyzed and visualized using MA plots and volcano plots in Fig. S2 to S5. The correlation coefficient of the expression values from the RNA-seq and quantitative real-time PCR (qRT-PCR) data was 0.8146 (Fig. S6). The values of the correlation coefficient are considered sufficient evidence of the reliability of RNA-seq data sets.

**Fig 3:**
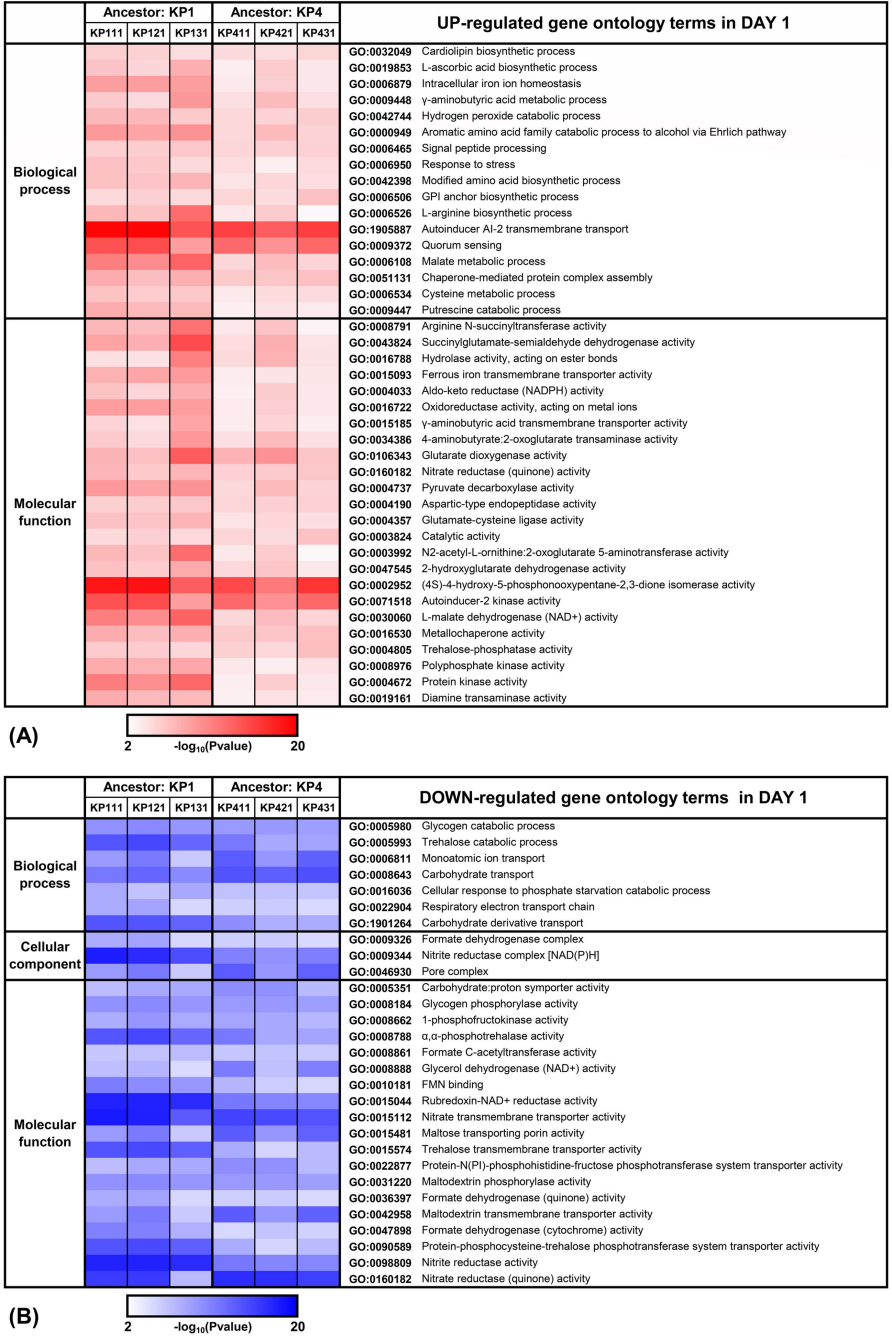
DAVID gene ontology analysis of adapted strains on day 1 of colistin adaptation. Overrepresentation analysis of significantly enriched genes in adapted strains versus ancestors identified GO terms meeting FDR <0.05 and ≥2-fold change thresholds. Panel (**A**) demonstrates up-regulated GO terms (red), while (**B**) shows down-regulated terms (blue), both observed on day 1 of adaptation. Color intensity corresponds to the −log₁₀(*P* value) of enrichment significance.

**Fig 4:**
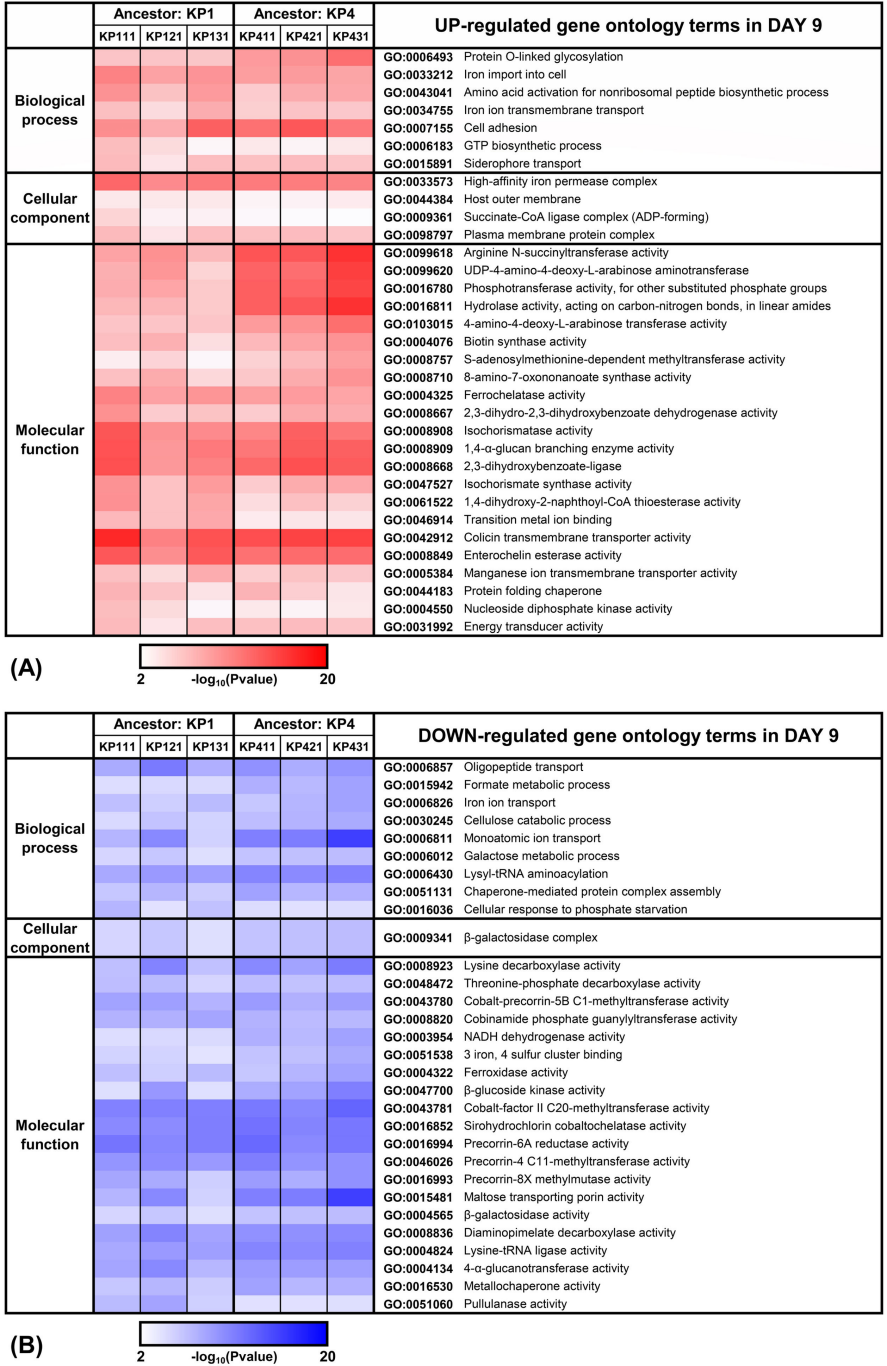
DAVID gene ontology analysis of adapted strains on day 9 of colistin adaptation. Overrepresentation analysis of significantly enriched genes in adapted strains versus ancestors identified GO terms meeting FDR <0.05 and ≥2-fold change thresholds. Panel (**A**) demonstrates up-regulated GO terms (red), while panel (**B**) shows down-regulated terms (blue), both observed on day 9 of adaptation. Color intensity corresponds to the −log₁₀(*P* value) of enrichment significance.

Gene ontology (GO) analysis revealed diverse differentially expressed genes across strains over time. Compared to the ancestor strain, the number of genes exhibiting differential expression (fold change > 2, FDR < 0.05) and those mapped to the ontology database are presented in Table S3. The analysis identified 96 to 434 up-regulated genes and 61 to 412 down-regulated genes. Variation was observed among the genes that were commonly enriched across both early and late adaptation phases. Heatmaps representing the enrichment patterns of these genes that were commonly and significantly enriched across all strains (more than twofold and FDR < 0.05) during early and late adaptation are shown in Fig. 3 and 4, respectively. During the early adaptation phase, the most prominently up-regulated GO terms were autoinducer AI-2 transmembrane transport and quorum sensing (QS), whereas nitrite reductase activity was observed as the most significantly down-regulated term (Fig. 3). Multiple GO terms were upregulated on day 9 of adaptation, including iron permease complex, colicin transmembrane transporter activity, and enterochelin esterase activity (Fig. 4).

**Fig 5:**
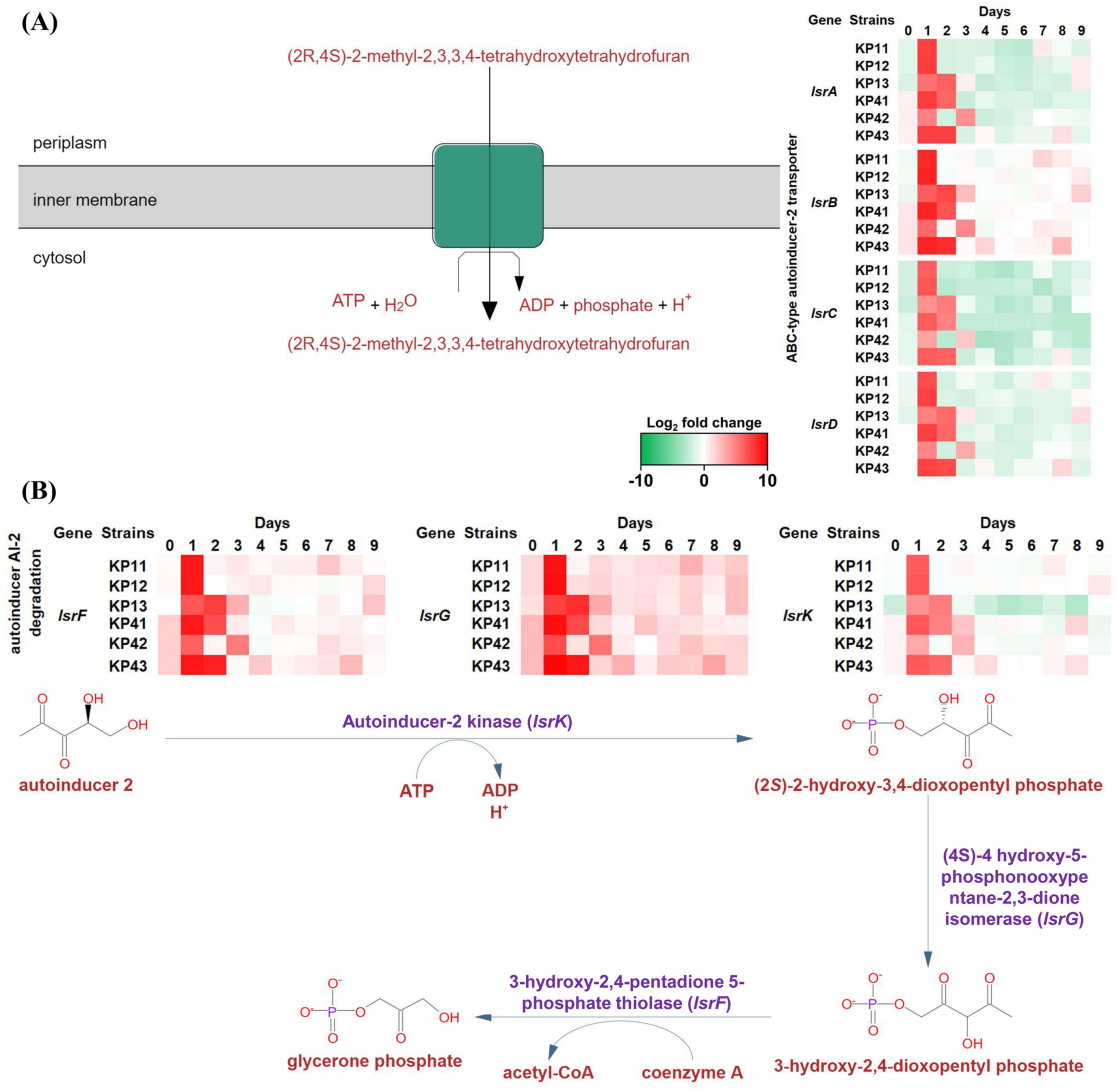
Differentially upregulated genes associated with AI-2 pathways between the first and second days in a given colistin pressure environment. Genes associated with the (**A**) autoinducer-2 ABC transporter and the (**B**) autoinducer AI-2 degradation pathway, such as *lsrABCD*, *lsrFG,* and *lsrK*, were confirmed to have increased during the first and second days of antibiotic pressure passages.

The ATP-binding cassette (ABC)-type autoinducer-2 transporter genes, *lsrABCD*, were observed to be upregulated (Fig. 5A), in the first to second days of the ALE passages. The average log_2_ fold change of *lsrABCD* in days 1–2 was 4.57 (21 DEGs), whereas the average log_2_ fold change for all other days, excluding days 1–2, was 0.74. The increased genes in only days 1–2 include *lsrFG* and *lsrK*, which are also an autoinducer AI-2 degradation pathway (Fig. 5B). The average log_2_ fold change of *lsrFG* and *lsrK* was 5.61 during days 1–2, whereas the value excluding these days was 0.80.

**Fig 6:**
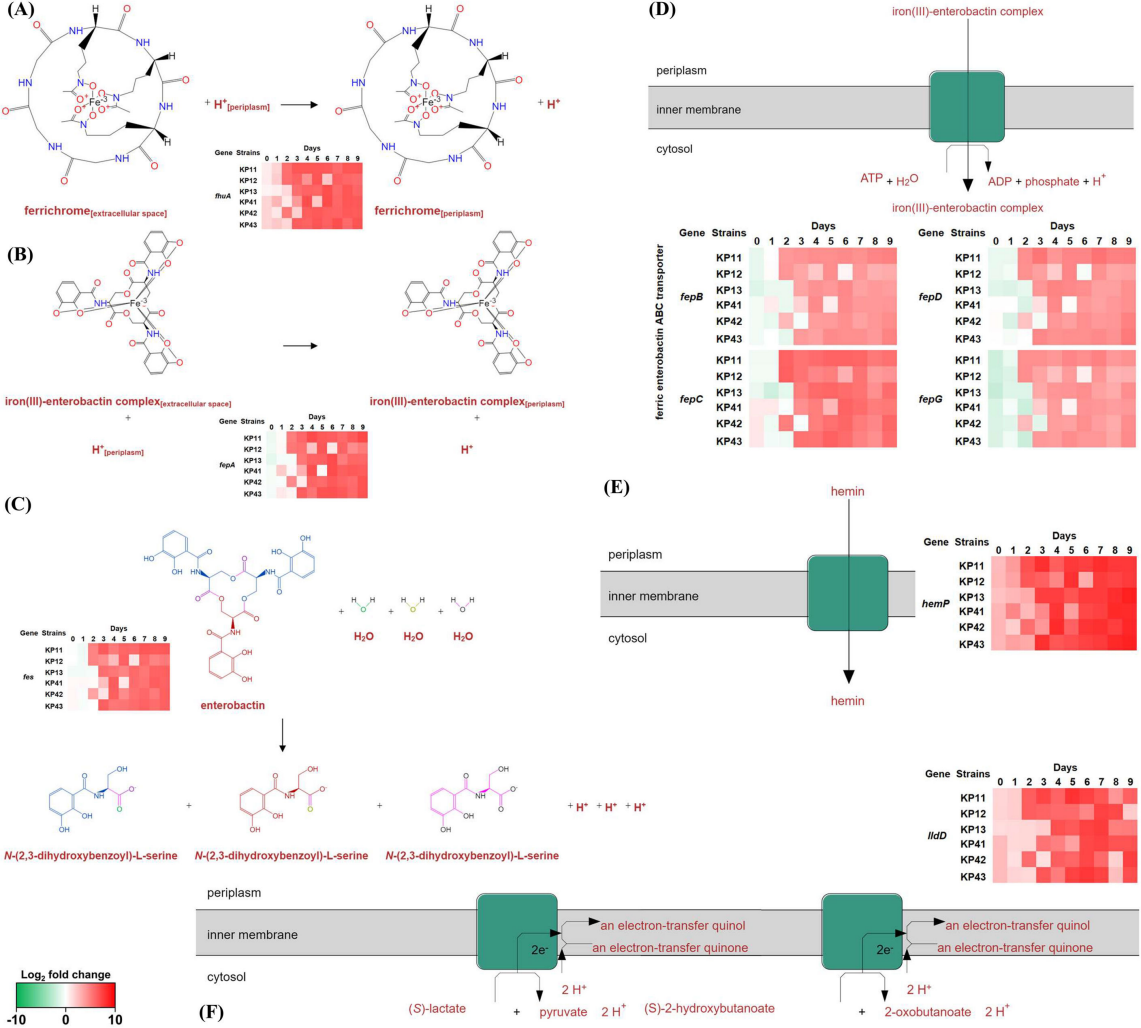
Differentially upregulated genes from the third day in the ALE procedure. A number of reactions are confirmed to be increased in all strains. The ferrichrome outer membrane transport complex gene *fhuA* (**A**) and the ferric enterobactin outer membrane transport complex gene *fepA* (**B**) were identified to be upregulated from the second day of antibiotic pressure cultures. The ferric enterochelin esterase harboring *fes* (**C**) was identified to be upregulated from day 2 onward. The genes associated with ferric enterobactin ABC transporter (**D**) also showed a significantly increased expression from the third day of antibiotic pressure. The gene regulating hemin uptake protein HemP (*hemP*) was identified to have increased in six strains after the third to fourth days of antibiotic pressure (**E**). The L lactate dehydrogenase gene (*lldD*) showed a significant increase (**F**) in all strains after passage day 4.

After the second day of antibiotic pressure, several genes were identified to be upregulated. The gene *fhuA*, which is involved in transporting ferrichrome from the extracellular space to the periplasm (Fig. 6A), shows upregulation from day 2 (17 DEGs; average log_2_ fold change 5.63) compared to the days prior (average log_2_ fold change 1.43). The gene *fepA*, associated with the ferric enterobactin outer membrane transport complex (Fig. 6B), showed upregulation starting from day 2 (17 DEGs; average log_2_ fold change 5.63), in comparison to previous days (average log_2_ fold change 1.43). The carboxyl ester hydrolases-associated gene *fes* was upregulated in most strains (Fig. 6C) from the second day (28 DEGs; average log_2_ fold change 4.89), relative to the preceding days (average log_2_ fold change −0.1). From day 2, ABC-type ferric enterobactin transporter genes *fepB*, *fepC*, *fepD*, and *fepG* (Fig. 4D) were upregulated (42 DEGs; average log_2_ fold change 3.31), compared to the days before (average log_2_ fold change −0.61). As shown in Fig. 6E, the hemin uptake protein HemP, regulating gene *hemP,* was also upregulated from day 3 (9 DEGs; average log_2_ fold change 7.26). The L-lactate dehydrogenase, associated with the gene *lldD*, is assumed to be increased in most strains (Fig. 6F) compared to before the second day (average log_2_ fold change of 1.90), and on the second day specifically (with 12 DEGs; average log_2_ fold change of 5.64).

**Fig 7:**
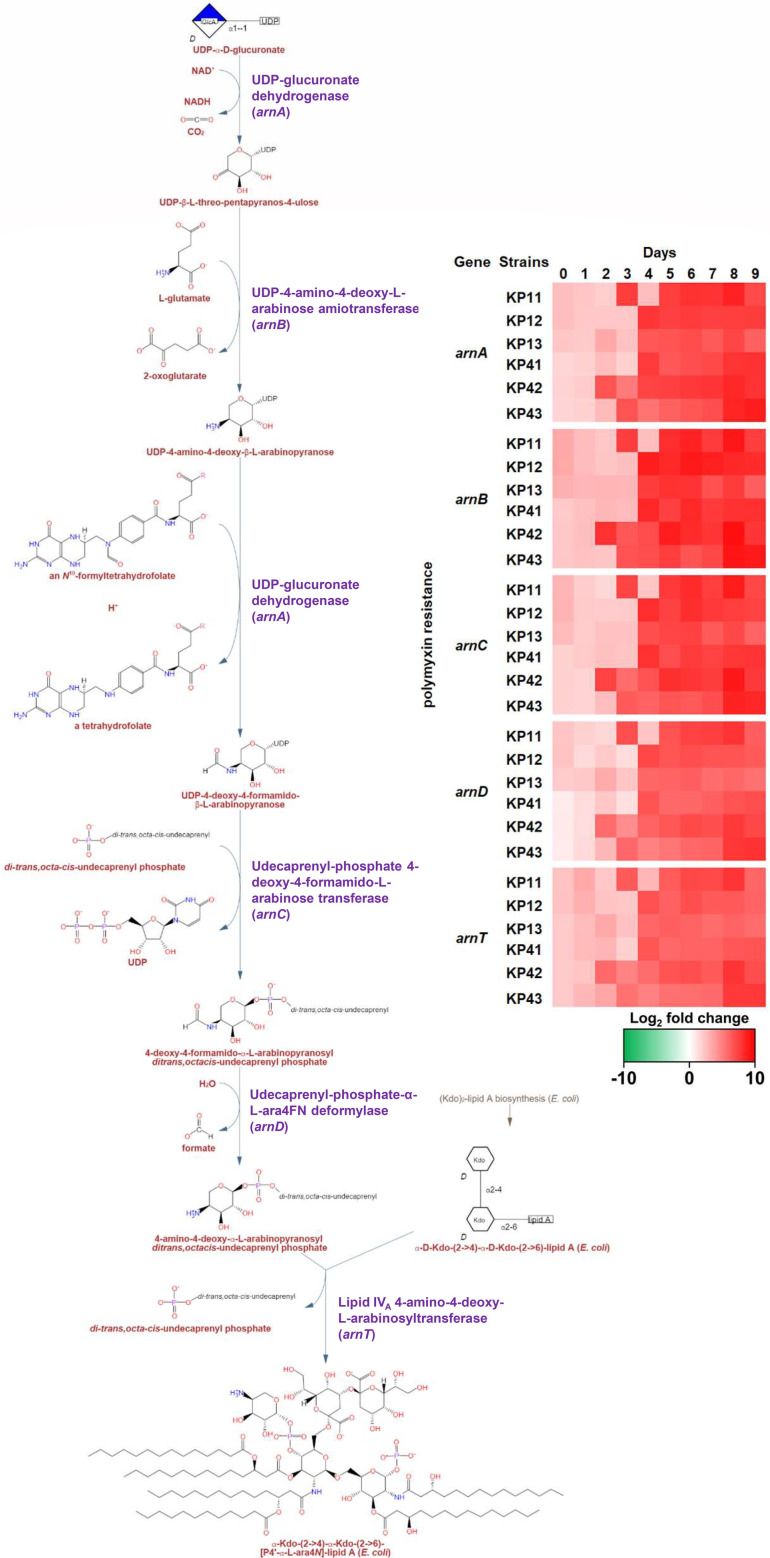
The up-regulated genes related to the polymyxin resistance pathway. The *arnBCADT* genes, which are associated with the regulation of the polymyxin resistance pathway, were identified to have increased from the third to fourth days of antibiotic pressure.

**Fig 8:**
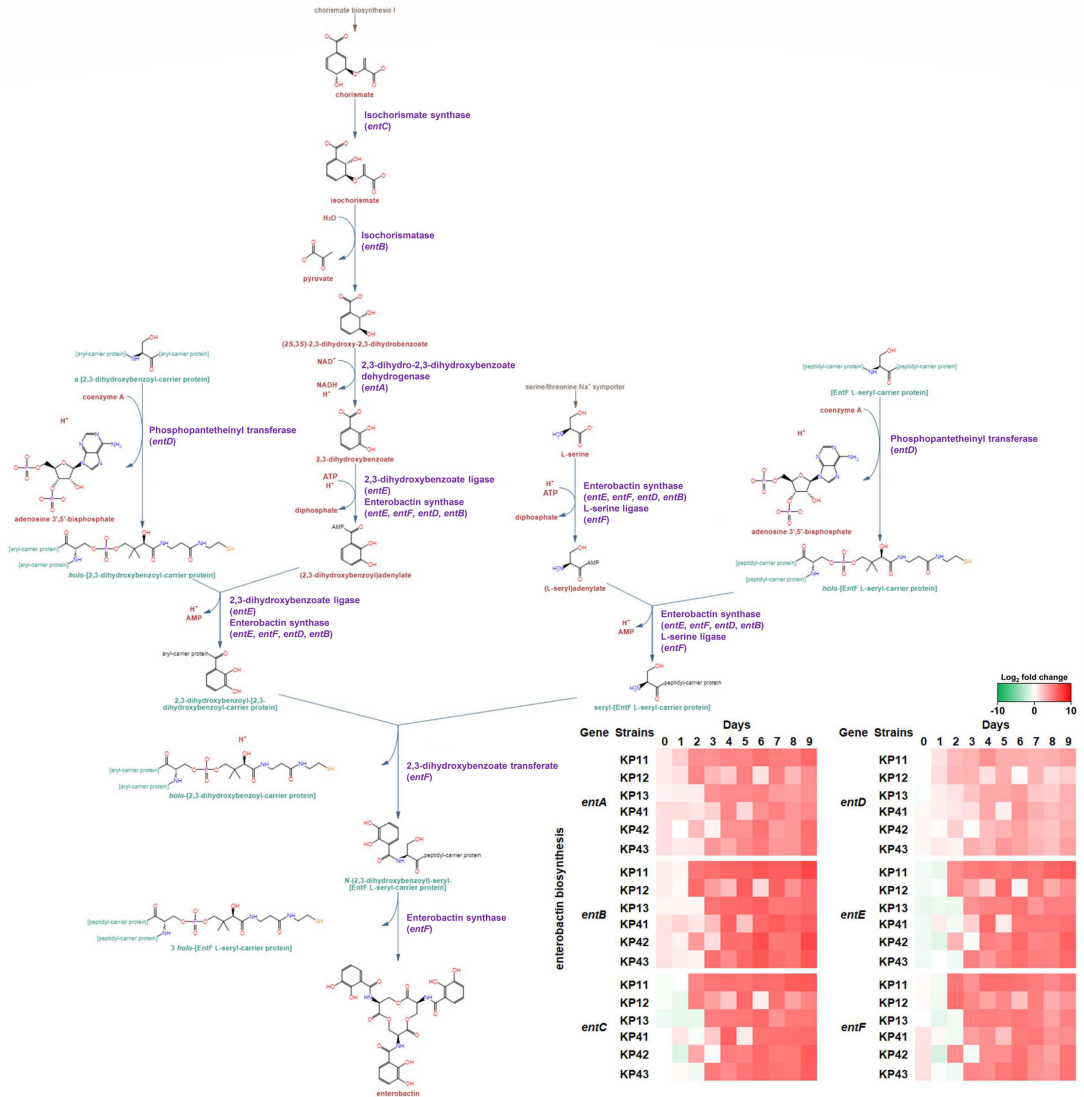
The genes related to the enterobactin biosynthesis pathway were confirmed to be elevated. Genes associated with the enterobactin biosynthesis pathway, *entCEBA*, *entD*, and *entF*, were confirmed to have increased from the second day of antibiotic pressure passages and remained elevated until the final passages.

Certain pathways, such as the polymyxin resistance pathway (Fig. 7) and the enterobactin biosynthesis pathway (Fig. 8), appear to play a significant role in the development of resistance in *K. pneumoniae* strains. Genes associated with those pathways displayed a clearly increasing trend from approximately the second to the third day under antibiotic pressure in most strains. While the average log_2_ fold change of *arnABCDT* (associated with polymyxin resistance) was 2.54 before the second day, it increased to 6.69 in the days following the second day (with 66 identified DEGs). The genes *entCEBA*, *entD*, and *entF* have increased from an average log_2_ fold change of 0.26 to 4.10 (62 DEGs) since the second day of antibiotic pressure.

**Fig 9:**
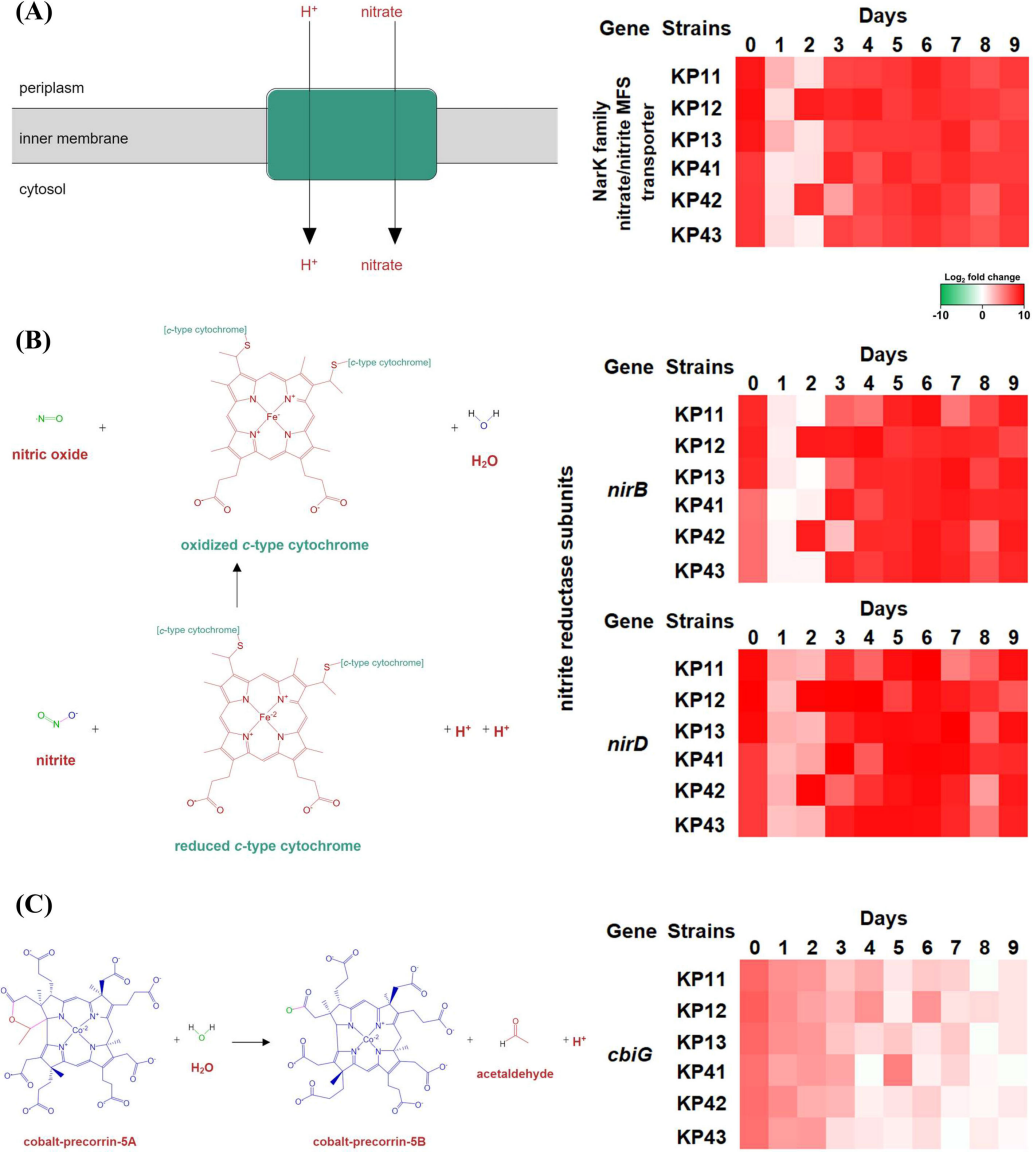
The reactions linked to the downregulation of genes during the antibiotic pressure passages. The NarK family nitrate/nitrite MFS transporter (**A**) and the nitrite reductase subunit harboring *nirD* and *nirB* (**B**) were identified to be temporarily downregulated on the first and second day of antibiotic pressure. Conversely, cobalt-precorrin 5A hydrolase gene *cbiG* (**C**) exhibited gradual downregulation throughout the passage.

Conversely, certain reactions showed a distinct decrease during specific periods of antibiotic pressure. The gene encoding the NarK family nitrate/nitrite MFS transporter exhibited a decrease during the first and second days of antibiotic pressure (11 DEGs and an average log_2_ fold change of 2.72, compared to the value of 7.75 for other days), followed by restoration of its expression levels (Fig. 9A). The genes of nitrite reductase reaction (Fig. 9B), *nirB* and *nirD*, were also downregulated (12 DEGs and an average log_2_ fold change of 3.00, compared to the value of 8.17 for other days). The average log_2_ fold change of cobalt-precorrin 5A hydrolase-related *cbiG* decreased from 5.93 on day 0 to 0.73 on the final day (Fig. 9C).

### Variated gene positions of the *in vitro* adaptive strains

**Fig 10:**
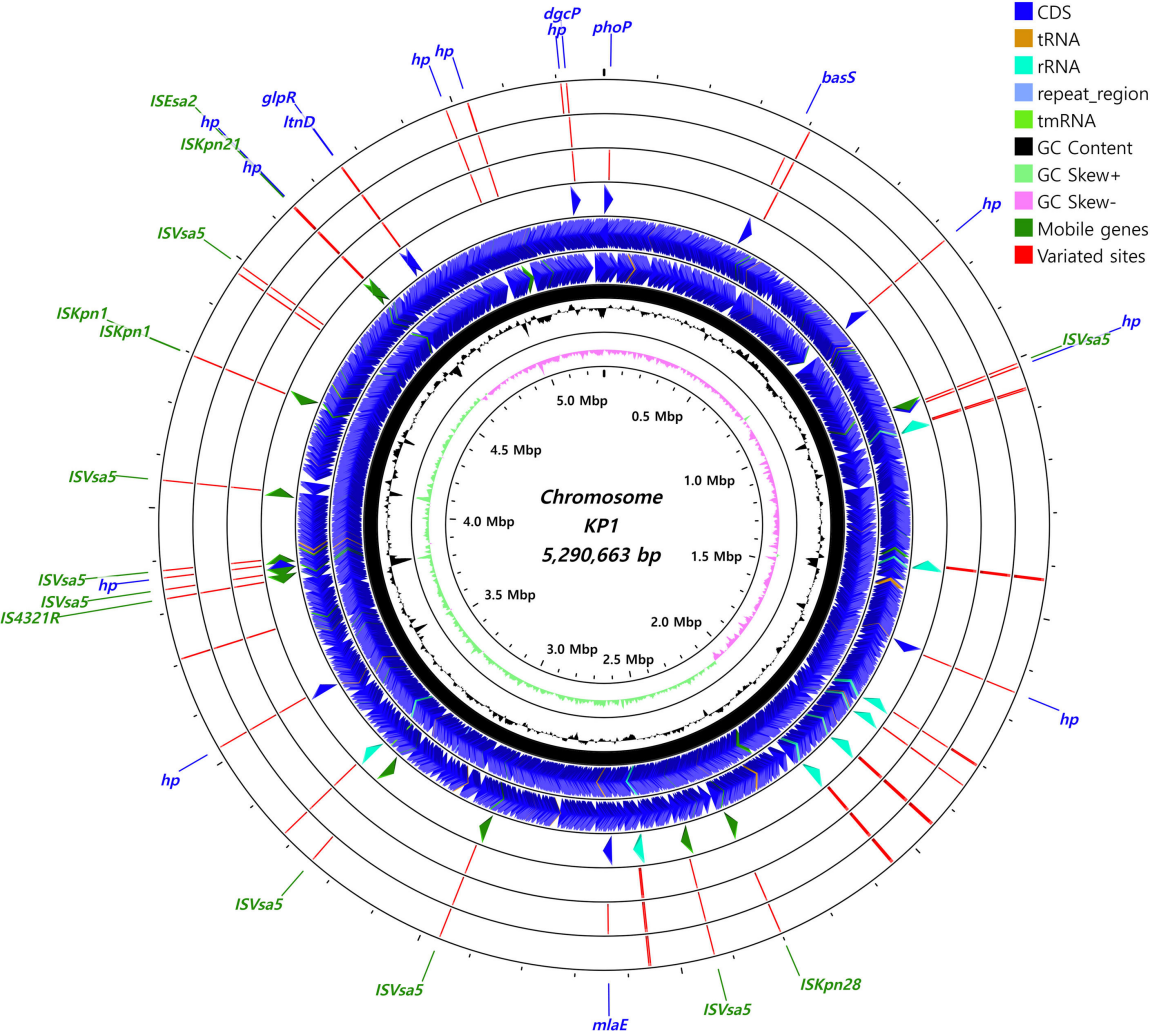
Visualization of the chromosomal map of the locations and characteristics of KP1 variants resulting from ALE, revealed by whole-genome resequencing. The genomic locations of each variant site are indicated with red strips in the order of 139, 129, and 119 from the outermost circle inward. The locations of the genes with discovered variants are additionally highlighted on the fourth circle from the outside. The positions of CDS genes are depicted as blue arrows, while mobile genes, such as transposases, are depicted as green arrows. CGView was used as the visualization tool for characterizing the genomic structures.

**Fig 11:**
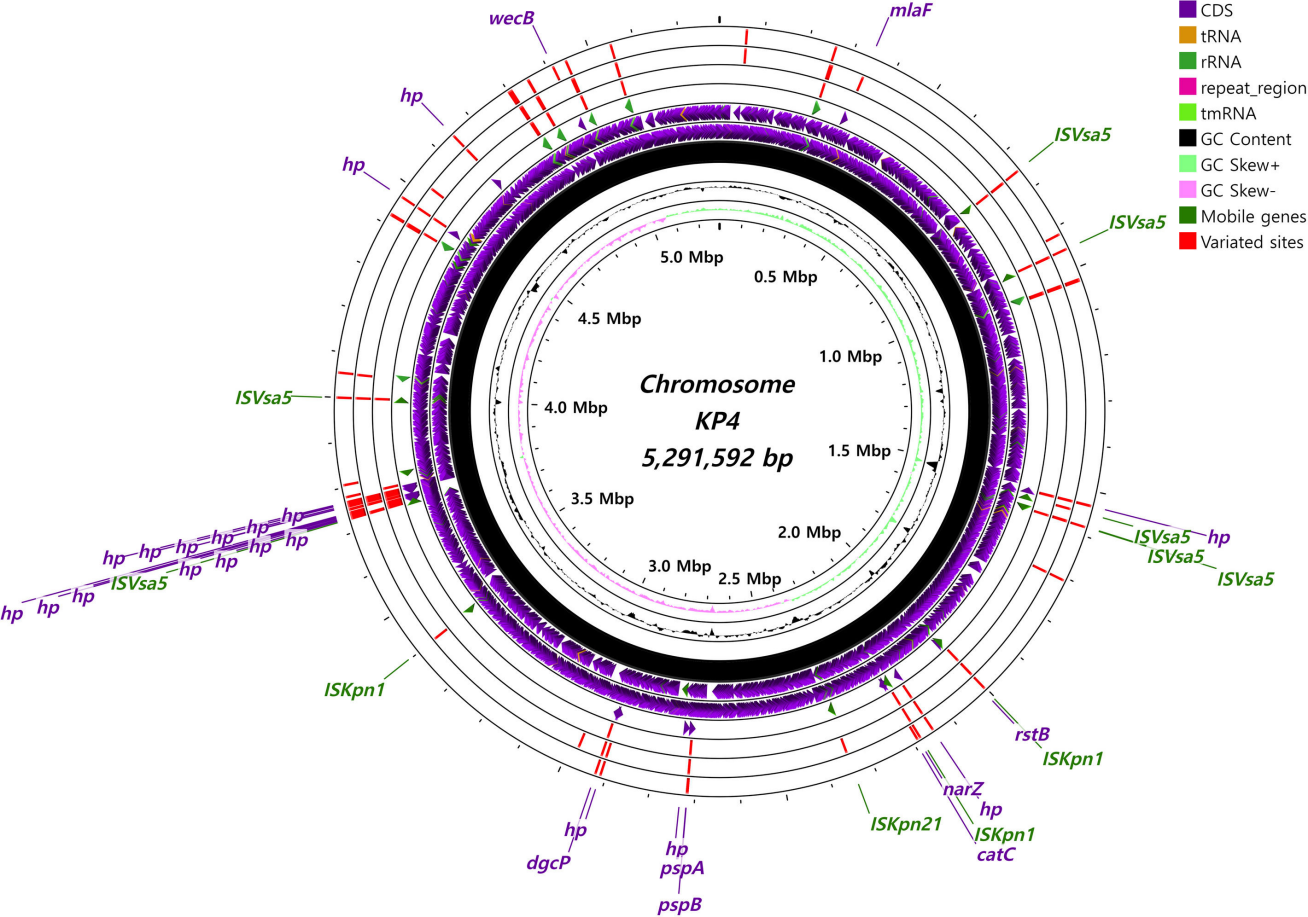
Visualization of the chromosomal map of the locations and characteristics of KP4 variants resulting from ALE, revealed by whole-genome resequencing. The genomic locations of each variant site are indicated with red strips in the order of 439, 429, and 419 from the outermost circle inward. The locations of the genes with discovered variants are additionally highlighted on the fourth circle from the outside. The positions of CDS genes are depicted as purple arrows, while mobile genes, such as transposases, are depicted as green arrows. CGView was used as the visualization tool for characterizing the genomic structures.

In order to characterize the genomic variations of adaptive strains, whole-genome resequencing was conducted on the six final isolates, namely KP119, KP129, KP139, KP419, KP429, and KP439. As a result of the whole-genome resequencing, a total of 70 and 80 CDS variation sites were identified in the KP1 and KP4 descendants, respectively, including multiple frameshift mutations (Tables S4 and S5). All gene segments, including the chromosomes (Fig. 10 and 11) and four heterogeneous plasmids (Fig. S7 and S8), were characterized to include CDS variations and visualized as schematic genomic maps.

Gene variation analysis revealed 19 frameshift mutations and 6 point mutations consistently observed across all KP1 descendant strains (Table S4). In contrast, KP4 descendants exhibited 23 frameshift mutations and 6 identical point mutations (Table S5). Most mutated genes were associated with mobile genetic elements, while 8 and 16 hypothetical proteins showed shifts in KP1 and KP4 descendant strains, respectively. However, commonly mutated sites across all six descendant strains were not observed. In addition, mutations directly associated with colistin resistance, which are frequently detected in clinical isolates, were not identified in the laboratory-adapted strains.

## DISCUSSION

In the early stages of the colistin pressure (days 1–2), the majority of the pressured species expressed high values of *lsrA*, *lsrB*, *lsrC*, and *lsrD* (Fig. 5A). The ABC transporter complex LsrABCD is recognized for its role in actively importing AI-2. The GO analysis revealed prominent enrichment of terms associated with AI-2 transmembrane transport, QS, and (4S)-4-hydroxy-5-phosphonooxypentane-2,3-dione isomerase across all strains (Fig. 3A). AI-2 is a signaling molecule assumed to enable interspecies communication among multiple species of bacteria and has been demonstrated to play an important role in bacterial drug resistance (21, 22). This transporter belongs to the ABC type, featuring two similar ATP-binding domains/proteins and two integral membrane domains/proteins. It functions as a bacterial enzyme that interacts with an extracytoplasmic substrate-binding protein, facilitating the uptake of the signaling molecule (2R,4S)-2-methyl-2,3,3,4-tetrahydoxytetrahydrofuran, also known as autoinducer-2 (23–25). Phosphorylated AI-2 has been shown to deactivate the transcriptional repressor, thus regulating the expression of QS-related genes (26). The AI-2 degradation pathway, associated with the upregulated *lsrFG* and *lsrK*, is known to be regulated by the presence of AI-2 (21). The enzyme (4S)-4-hydroxy-5-phosphonooxypentane-2,3-dione isomerase, encoded by the *lsrG*, is a key component of the Lsr operon, which is responsible for processing the AI-2 (27). Quorum sensing occurs through the exchange of extracellular signaling molecules known as autoinducers (28). This mechanism enables bacterial populations to coordinate gene expression based on cell density, leading to various cooperative behaviors, such as bioluminescence, virulence factor expression, antibiotic and biofilm production (28). Although the traditional view is that the signaling molecule accumulates extracellularly, certain bacteria, like *E. coli*, can uptake and degrade the autoinducer AI-2 (28). In 2024, a report by Feizi et al. (29) revealed numerous varied protein expressions, including LsrA, in *E. coli* DH5-α, following the acquisition of colistin resistance genes. The autoinducer AI-2 pathways have been reported to be associated with QS and biofilm synthesis, thereby contributing to AMR in *K. pneumoniae* (30, 31). Therefore, the findings of this study can be considered evidence supporting the speculation that *K. pneumoniae* isolates utilize QS mechanisms only in the early days of bacterial survival under colistin pressure.

After the second day of antibiotic pressure, other genes were identified to be upregulated (Fig. 4A). Overall, the overexpressed genes observed after the second day of resistance development included multiple reactions related to the iron uptake system (Fig. 6), such as *fhuA* (ferrichrome), *fepA,* and *fepBCDG* (enterobactin). The enterochelin esterase gene (*fes*) is associated with reactions that express the transformation of enterobactin + 3 H_2_O → 3N-(2,3-dihydroxybenzoyl)-L-serine + 3H^+^ (32). The upregulated genes from day 2 included ferric enterobactin ABC transporter regulating genes *fepBCDG*. The ferric enterobactin ABC transporter is recognized as an ABC-type transporter, characterized by two identical ATP-binding domains/proteins and two integral membrane domains/proteins (33–35). Additionally, it has been described to function as a bacterial enzyme interacting with an extracytoplasmic substrate-binding protein, facilitating the high-affinity uptake of Fe^3+^-enterobactin complexes. The reaction catalyzed by the ABC-type ferric enterobactin transporter (associated with gene *fepBCDG*) is expressed as follows: ATP + iron(III)-enterobactin complex_[periplasm]_ + H_2_O → ADP + iron(III)-enterobactin complex_[cytosol]_ + phosphate + H^+^ (36). The upregulated enzyme encompasses the L-lactate dehydrogenase, an flavin mononucleotide (FMN)-dependent membrane-associated dehydrogenase that operates in both aerobic and anaerobic nitrate respiration (37–40). To sum it up, it is assumed that after the third day of colistin resistance development, there would have been an increase in the use of enterobactin as an iron source. On the other hand, more work is needed to define the exact correlations between colistin resistance development and the actions of L-lactate dehydrogenase.

The upregulated pathways included the polymyxin resistance pathway (Fig. 7), which showed upregulation from the third day of antibiotic pressure and remained upregulated until the last day. Various strategies have been previously documented for developing resistance to polymyxins in several bacterial species. One such strategy involves the addition of L-Ara4N groups to phosphate groups within the lipid A structure, as revealed by research assessing polymyxin-resistant *E. coli* mutants (41–46). This modification increases the charge of lipid A and decreases the affinity for positively charged drugs (47). Although it is not surprising to observe the upregulation of the polymyxin resistance pathway under colistin pressure, the consistent upregulation starting from the third day of pressure in all six observed strains should be further assessed in detail. It is hypothesized that *lsr* operon overexpression may reduce *arnT* expression and L-Ara4N synthesis through ATP/NADPH depletion during AI-2 degradation, as both pathways compete for these metabolic resources (27, 48). Additionally, increased AI-2 breakdown could amplify RpoS-mediated stress signaling, potentially suppressing the PmrA-PhoPQ signaling pathway and consequently downregulating *arn* operon expression (49). Therefore, future studies confirming the relationship between AI-2 degradation mechanisms and *arn* operon expression in *Klebsiella* species would significantly advance our understanding of bacterial temporal adaptation strategies.

Enterobactin, a catecholate iron transport siderophore found in Enterobacterales (50), is a cyclic molecule that serves as a precursor to 2,3-dihydroxy-N-benzoylserine, enabling the transportation of iron into bacterial cells (34, 51). Enterobactin is also known as a siderophore with an exceptionally high affinity for Fe^3+^ due to the presence of 2,3-dihydroxybenzamide motifs, is synthesized in response to iron deficiency (52, 53). The synthesis of enterobactin comprises two stages: the conversion of chorismate to 2,3-dihydroxybenzoate, and the subsequent synthesis of enterobactin from 2,3-dihydroxybenzoate and L-serine (54). The importance of the iron uptake system in colistin resistance has been consistently demonstrated in recent studies involving *K. pneumoniae* and *E. coli* strains. Enhanced iron uptake, mediated by the enterobactin synthesis genes *entCEBA*, is known to activate the PmrAB-PhoPQ signaling pathways, leading to lipid A modification and increased colistin resistance. Iron acquisition through the enterobactin-iron complex system, which involves the coordinated action of *fepA*, *fepBCDG*, and *fes* genes, also contributes to the activation of the PmrAB-PhoPQ pathway and subsequent colistin resistance (10, 55, 56). The findings of this study provide further evidence, highlighting delayed adaptation strategies to colistin observed in *K. pneumoniae* strains. Combined approaches targeting these mechanisms—such as the use of kaempferol to inhibit *entCEBA* and *fepA* function—have been demonstrated to significantly enhance colistin susceptibility when used in conjunction with colistin (55).

The genes associated with nitrate transportation and nitric oxide (NO) formation via nitrite reductase were observed to decrease (Fig. 9) in most strains during the first and second days of antibiotic pressure. The reaction that exhibited a gradual decrease involved hydrolysis catalyzed by cobalt-precorrin 5A hydrolase, which targets the ring A acetate δ-lactone of cobalt-precorrin-5A, thereby resulting in the loss of the C-20 carbon and its attached methyl group in the form of acetaldehyde (57, 58). There are more works needed to be done to completely reveal the relations between colistin resistance development and speculatively variated reaction of enzymes observed in this study, such as nitrate reductase (*nirB* and *nirD*) and cobalt-precorrin 5A hydrolase (*cbiG*).

Whole-genome resequencing was conducted to identify variant gene positions, revealing 58 frameshifted CDS genes, including multiple unidentified hypothetical proteins and mobile gene elements (Fig. 10 and 11). It is suspected that genes correlated with the disrupted mobile gene elements may be significantly related to bacterial survival against colistin in *K. pneumoniae*. The functions of genes with sequence variations commonly observed across the adapted strains (KP119 to KP419), including hypothetical proteins, could not be confirmed. Sequence changes previously known to be associated with colistin resistance in field isolates were not identified, suggesting that the observed variations may be limited to laboratory conditions. However, even if these variations are limited to *in vitro* conditions, further investigations of these regions could provide new insights into colistin adaptation procedures in *Klebsiella* species. The functions of these genes should be thoroughly characterized through further studies.

In the growth rate comparison (Fig. S1) between the generated colistin-adapted strain and the ancestor strains (KP1 and KP4), no significant differences in growth were observed. This result is consistent with previous reports, which indicate that colistin resistance mutations do not necessarily impose a significant impact on growth capacity or fitness cost (59, 60). As shown by Aulin et al., colistin resistance caused by mutations in genes, such as *pmrAB*, *phoPQ*, *ccrAB*, or *mgrB,* did not always lead to a significant impairment of bacterial growth (59). The nosocomial *K. pneumoniae* strains reported by Janssen et al. demonstrated that the acquisition of various evolutionary resistance mechanisms under colistin pressure, such as lipid A modification, did not necessarily affect bacterial growth capacity (60).

To summarize, this study found that QS molecules were upregulated in the early stages of resistance development, while the iron uptake system and polymyxin resistance pathway via target modification were upregulated in the later stages. Considering the stability of the resistance capacity of the isolates shown in Fig. 1, it is speculated that the isolates use QS mechanisms, such as biofilm formation, in the early stages to survive under colistin pressure until they develop resistance strategies using the iron uptake system and polymyxin resistance pathway. Therefore, the possible future strategies to prevent the *K. pneumoniae* isolates from developing resistance would include the results of the recent studies subjecting quorum quenching, the disruption of QS signaling, in the early stages of colistin pressure (61).

However, further phenotypic validation under identical experimental conditions is necessary, as this study is limited to transcriptomic and genomic observations of processes, such as QS mechanisms and iron uptake systems, in *Klebsiella* strains. Furthermore, since the outcomes in this study were observed under artificial selective pressure in laboratory conditions, it is important to evaluate how these findings might differ in environments involving host microbial communities and immune responses. The confirmation through replication of the identical adaptation process in animal infection models should be conducted to validate whether the bacterial delayed adaptation strategy using interplay switching of QS and polymyxin resistance pathway function is identical *in vivo*.

## MATERIALS AND METHODS

### Strain selection and adaptive laboratory evolution

For analysis, two previously characterized *K. pneumoniae* strains (formerly named DMKP1 and DMKP4) (62) were chosen for the ALE experiment. The MICs of the *K. pneumoniae* strains against colistin were determined using broth microdilution methods according to the guidelines suggested by the Clinical and Laboratory Standards Institute (63). Protocols modified from previously reported methods (64) were performed to confer colistin pressure to the strains. The laboratory evolution passages were performed on 96-well round-bottom plates containing 100 µL of colistin-supplemented cation-adjusted Mueller-Hinton broth (CAMHB) for 9 days. The plates were designed to generate a colistin gradient and select strains with the highest resistance capacity. The gradient spanned concentrations ranging from 0 to 256 µg/mL at extended intervals. Dilutions were prepared in a twofold manner, starting from 256 and 192 µg/mL. The bacterial concentration in each well of the microtiter plate was adjusted to 5 × 10^5^ colony-forming units/mL (CFU/mL). All cultures were incubated for 16 h, and growth was confirmed using the VersaMax absorbance microplate reader (Molecular Devices, CA, USA) by determining if the optical density reading at 600 nm was ≥0.1. After a cycle of evolutionary growth, the MIC was determined by identifying the well with the lowest concentration that confirmed growth inhibition. The cultures grown at the highest drug concentration (just below the MIC concentration) were re-diluted for the subsequent evolutionary cycle after being checked for contamination by spreading them on an antibiotic-free sheep blood agar plate. To confirm the stability of each strain’s MIC capacity, each strain was cultured on fresh medium without antibiotic supplementation for 6 days, following the endpoint of the ALE experiments.

### Simple colony formation assay and growth rate comparison

To track and identify phenotypically resistant populations in the ALE experiment, a simple colony formation assay was conducted at each stage of the evolution experiments, with slight modifications to a previously reported method (64). Specifically, after overnight growth of each strain in fresh LB, each batch was diluted until a dilution factor of 10^6^ was achieved. Diluted cultures were spotted in volumes of up to 10 µL on antibiotic-free LA plates and colistin-supplemented (4 µg/mL) LA plates, for total and resistant CFU population counts, respectively. After adjusting the dilution factor, the CFU/mL numbers were used to determine the resistant ratio by dividing the count of resistant bacteria by the total bacterial count. After CFU enumeration, the corresponding colonies were photographed and included as images. Since transparent plates without colonies also captured the textured background of the table, all available original photographs were included as Supplementary material to prevent misinterpretation of these textures as false positives.

Growth rates were compared by analyzing growth curves in fresh Luria-Bertani (LB) broth. Overnight cultures of each strain were inoculated at a 1:1,000 ratio and incubated at 37°C with shaking at 220 rpm. Growth was monitored by measuring the OD_600_ hourly over a 12 h period. The assay was performed in triplicate, and statistical significance was determined using one-way analysis of variance, with a significance threshold of *P* < 0.05.

### Total RNA purification

The extraction procedure commenced with centrifugation at 3,800 rpm for 10 min at 4°C, resulting in the formation of a bacterial cell pellet. The pellet was subsequently washed twice with PBS. Following the removal of the supernatant, the extraction procedure continued without interruption. In detail, a solution comprising 0.5 mL containing 1% SDS (vol/vol), 160 mM.

Ethylenediaminetetraacetic acid, and lysozyme (50 mg/mL) was added to the cell pellet. The mixture was then transferred to a 2 mL Lo-bind tube containing sterilized glass balls. Subsequently, the solution was incubated at room temperature for 5 min to facilitate cell lysis by the lysozyme. Following this step, 100 µL of phenol-chloroform-isoamyl alcohol (Carl Roth, Karlsruhe, Germany) was added to the mixture and vortexed to achieve cell lysis. Subsequently, 400 µL of buffer RLT (QIAGEN, Hilden, Germany) containing β-mercaptoethanol (100:1) was added, and the mixture was incubated on ice for 10 min. This process was repeated for three cycles, involving vortexing, followed by incubation on ice. Afterward, 1 mL of phenol-chloroform-isoamyl alcohol was added. The Lo-bind tubes were then centrifuged for 15 min, and the aqueous phase was transferred to a new tube containing 700 µL of ethanol.

Total RNA extraction was carried out using the QIAGEN RNeasy Mini Kit as per the manufacturer’s instructions. The sample was transferred to an RNeasy Mini spin column placed in a 2 mL collection tube. Centrifugation was performed for 15 s at 8,000 × *g*, and the flow-through was discarded. Subsequently, 700 µL of Buffer RW1 was added to the RNeasy spin column and centrifuged for 15 s at 8,000 × *g*. Then, 500 µL of Buffer RPE was added to the RNeasy spin column and centrifuged for another 15 s at 8,000 × *g*. Following this, another 500 µL of Buffer RPE was added to the RNeasy spin column, and centrifugation was carried out for 2 min at 8,000 × *g*. The RNeasy spin column was transferred to a new 1.5 mL collection tube. Then, 50 µL of RNase-free water was added directly to the spin column membrane, and centrifugation was performed for 1 min at 8,000 × *g* to elute the RNA.

### Total RNA sequencing and bioinformatic analysis

The RNA purity was assessed by analyzing 1  µL of total RNA extract using a NanoDrop 1000 spectrophotometer (Thermo Fisher Scientific, Waltham, MA, USA). The integrity of the RNA was evaluated with an Agilent 2100 Bioanalyzer (Agilent Technologies, Santa Clara, CA, USA), which provided an RNA integrity number value. The total RNA sequencing library was prepared following the manufacturer’s instructions for the TruSeq Stranded Total RNA Sample prep kit with Ribo-Zero Plus rRNA Depletion kit (Illumina, San Diego, California, USA). Ribosomal RNA (rRNA) was removed from purified total RNA using DNA probes targeting rRNA. Following rRNA depletion, the samples were reverse transcribed into cDNA using reverse transcriptase and random hexamers, with dUTP incorporated in place of dTTP during amplification. These cDNA fragments were then appended with a single “A” base and ligated with adapters. The resultant products underwent purification and enrichment via PCR to form the final library. The quality of the final library was assessed through automated electrophoresis on an Agilent TapeStation system, and its concentration was determined via quantitative PCR (qPCR) using KAPA SYBR FAST qPCR Master Mix (Kapa Biosystems, Wilmington, MA, USA). Sequencing was conducted on an Illumina NovaSeq 6000 system following the provided protocols for 2 × 100 sequencing.

The raw data of RNA-Seq were aligned and mapped to the reference genome derived from *K. pneumoniae* KP-1 gene (NCBI Reference Sequence NZ_CP012883.1) using kallisto (65). The Kallisto algorithm was also applied to predict variant abundance per reference sequence. Calculations of transcript per million by trimmed mean of M values normalization and expected counts for each gene were conducted using the RSEM software package (66). Two widely used statistical analysis methods (edgeR and DESeq2) were applied to identify DEGs (67, 68). The DEGs of annotated mRNA transcripts were calculated using edgeR and selected based on the FDR *P* value of  <0.05. Significantly (FDR of <0.1) differentially expressed genes were identified using a log_2_ fold change in expression greater than 1.0. Pathway gene sets are established through consultation of the EcoCyc database (69).

To analyze the gene ontology and Kyoto Encyclopedia of Genes and Genomes (KEGG) pathway enrichment of adapted strains, functional annotation and mapping of transcripts to reference genes were performed using DIAMOND (version 2.1.11, https://github.com/bbuchfink/diamond/releases/tag/v2.1.11) (70) with the BLASTx option for sensitive protein sequence comparison against translated nucleotide databases, applying an e-value threshold of 1 × 10⁻⁵ (default: 0.001), retaining only alignments within the top 1% of the best alignment scores, and using the BLOSUM62 scoring matrix as the default setting. The resulting gene lists were then subjected to DAVID (version 6.8) (71, 72) for GO term and KEGG pathway enrichment analysis, with a significance threshold for enriched terms and pathways set at a *P* value of <0.05.

To verify the RNA-seq analysis data, six genes (*lsrB, lsrD, lsrG, arnA, arnC,* and *entB*) identified in the RNA-seq results were selected and subjected to qRT-PCR at time points from day 0 to day 5. The cDNA was synthesized with reverse transcription using a cDNA using the MultiScribe Reverse Transcriptase (Applied Biosystems, Inc.) according to the manufacturer’s directions. Real-time PCR was performed with 1 µL of cDNA using EzAMP TM qPCR 2× Master Mix SYBR (Elpis Biotech) following the manufacturer’s procedures and Rotor-Gene Q real-time PCR cycler (Qiagen). The cycling parameters were as follows: 95°C for 10 min for one cycle, followed by 45 cycles of 95°C for 15 s and 60°C for 45 s. The 16S rRNA gene was used as an internal control. The primers used are listed in Table S6. Relative expression was calculated using the comparative CT (2^−ΔΔCT^) method, and the Pearson correlation coefficient was calculated between RNA-seq and qRT-PCR results (73).

### Whole-genome resequencing and analysis

To evaluate genomic variations that occurred as a result of the ALE, whole-genome resequencing was conducted. Three descendants of each KP1 and KP4 were resequenced using the previously identified whole-genome data sets of the ancestral isolates. For the whole-genome resequencing, genomic libraries (550 bp insert size) preparation was conducted using the TruSeq DNA PCR-Free Low Throughput Library Prep Kit (Illumina, Inc., San Diego, CA, USA). Libraries were sequenced on the NovaSeq 6000 platform with paired-end (2 × 150 bp).

Variant calling was conducted with the nfcore/sarek 2.7.1 pipeline following Genome Analysis Toolkit (GATK) v4.1.7.0 (74) best practices, FreeBayes v1.3.2 (75), and SAMtools v1.9 (76) (using htslib 1.9 [77]) for the identification of single nucleotide variants (SNVs). Variant calling for SNVs was conducted by Strelka v2.9.10 (78), MSIsensor v0.5 (79), Control-FREEC v11.6 (80), and structural variants were identified using Manta v1.6.0 (81). Copy numbers were called by AlleleCount v4.0.2 and ASCAT v2.5.2 (82). Annotation of the structural variants was done using a combination of SnpEff v4.3 (83) and VEP v99.2. The quality control of the raw sequencing reads was performed with FastQC v0.11.9 (84) and MultiQC v1.8 (85). The result files were merged using bcftools v1.9 (86). Copy number was estimated using CNVkit v0.9.6 (87). Quality control statistics were computed with QualiMap v.2.2.2 -dev (88). The raw reads were filtered using Trim Galore v0.6.4_dev (https://www.bioinformatics.babraham.ac.uk/projects/trim_galore/) to remove low-quality reads, and VCFtool v0.1.16 (89) was used to perform further filtering of the samples. Statistical analysis was done with the R package (v4.0.2) and visualized using the CGView (90).

## Data Availability

All raw sequencing data analyzed in this study have been uploaded to the NCBI Sequence Read Archive (SRA) database under accession number PRJNA1276715.

## SUPPLEMENTAL MATERIAL

Fig. S9 spectrum.03428-24-s0001.pdf Original data complementing Fig. 2B.

Supplemental material spectrum.03428-24-s0002.pdf Supplemental figures and tables.

ASM does not own the copyrights to Supplemental Material that may be linked to, or accessed through, an article. The authors have granted ASM a non-exclusive, world-wide license to publish the Supplemental Material files. Please contact the corresponding author directly for reuse.

## References

[1] Navon-Venezia S, Kondratyeva K, Carattoli A. 2017. Klebsiella pneumoniae: a major worldwide source and shuttle for antibiotic resistance. FEMS Microbiol Rev 41:252–275. doi:10.1093/femsre/fux013 28521338

[2] Paczosa MK, Mecsas J. 2016. Klebsiella pneumoniae: going on the offense with a strong defense. Microbiol Mol Biol Rev 80:629–661. doi:10.1128/MMBR.00078-15 27307579 PMC4981674

[3] Kunin CM, Bugg A. 1971. Binding of polymyxin antibiotics to tissues: the major determinant of distribution and persistence in the body. J Infect Dis 124:394–400. doi:10.1093/infdis/124.4.394 4335457

[4] Yow EM, Tan E, Shane L, Schonfeld S, Abu-nassar H. 1961. Colistin (coly-mycin) in resistant bacterial infections. A clinical appraisal. Arch Intern Med 108:664–670. doi:10.1001/archinte.1961.03620110004002 14009411

[5] Levin AS, Barone AA, Penço J, Santos MV, Marinho IS, Arruda EA, Manrique EI, Costa SF. 1999. Intravenous colistin as therapy for nosocomial infections caused by multidrug-resistant Pseudomonas aeruginosa and Acinetobacter baumannii. Clin Infect Dis 28:1008–1011. doi:10.1086/514732 10452626

[6] Catchpole CR, Andrews JM, Brenwald N, Wise R. 1997. A reassessment of the in-vitro activity of colistin sulphomethate sodium. J Antimicrob Chemother 39:255–260. doi:10.1093/jac/39.2.255 9069549

[7] Hamel M, Rolain J-M, Baron SA. 2021. The history of colistin resistance mechanisms in bacteria: progress and challenges. Microorganisms 9:442. doi:10.3390/microorganisms9020442 33672663 PMC7924381

[8] Riquelme MP, Martinez RW, Brito B, García P, Legarraga P, Wozniak A. 2023. Chromosome-mediated colistin resistance in clinical isolates of Klebsiella pneumoniae and Escherichia coli: mutation analysis in the light of genetic background. Infect Drug Resist 16:6451–6462. doi:10.2147/IDR.S427398 37789836 PMC10544214

[9] Ewers C, Göpel L, Prenger-Berninghoff E, Semmler T, Kerner K, Bauerfeind R. 2022. Occurrence of mcr-1 and mcr-2 colistin resistance genes in porcine Escherichia coli isolates (2010–2020) and genomic characterization of mcr-2-positive E. coli. Front Microbiol 13:1076315. doi:10.3389/fmicb.2022.1076315 36569100 PMC9780603

[10] Humphrey M, Larrouy-Maumus GJ, Furniss RCD, Mavridou DAI, Sabnis A, Edwards AM. 2021. Colistin resistance in Escherichia coli confers protection of the cytoplasmic but not outer membrane from the polymyxin antibiotic. Microbiology 167:001104. doi:10.1099/mic.0.001104 34723787 PMC8743629

[11] Ding Y, Hao J, Xiao W, Ye C, Xiao X, Jian C, Tang M, Li G, Liu J, Zeng Z. 2023. Role of efflux pumps, their inhibitors, and regulators in colistin resistance. Front Microbiol 14:1207441. doi:10.3389/fmicb.2023.1207441 37601369 PMC10436536

[12] Paveenkittiporn W, Kamjumphol W, Ungcharoen R, Kerdsin A. 2020. Whole-genome sequencing of clinically isolated carbapenem-resistant Enterobacterales harboring mcr genes in Thailand, 2016–2019. Front Microbiol 11:586368. doi:10.3389/fmicb.2020.586368 33505364 PMC7829498

[13] Carroll SP, Jørgensen PS, Kinnison MT, Bergstrom CT, Denison RF, Gluckman P, Smith TB, Strauss SY, Tabashnik BE. 2014. Applying evolutionary biology to address global challenges. Science 346:1245993. doi:10.1126/science.1245993 25213376 PMC4245030

[14] Sandberg TE, Salazar MJ, Weng LL, Palsson BO, Feist AM. 2019. The emergence of adaptive laboratory evolution as an efficient tool for biological discovery and industrial biotechnology. Metab Eng 56:1–16. doi:10.1016/j.ymben.2019.08.004 31401242 PMC6944292

[15] Novick A, Szilard L. 1950. Experiments with the chemostat on spontaneous mutations of bacteria. Proc Natl Acad Sci USA 36:708–719. doi:10.1073/pnas.36.12.708 14808160 PMC1063276

[16] Elena SF, Lenski RE. 2003. Evolution experiments with microorganisms: the dynamics and genetic bases of adaptation. Nat Rev Genet 4:457–469. doi:10.1038/nrg1088 12776215

[17] Barrick JE, Lenski RE. 2013. Genome dynamics during experimental evolution. Nat Rev Genet 14:827–839. doi:10.1038/nrg3564 24166031 PMC4239992

[18] Lenski RE, Rose MR, Simpson SC, Tadler SC. 1991. Long-term experimental evolution in Escherichia coli. I. Adaptation and divergence during 2,000 generations. Am Nat 138:1315–1341. doi:10.1086/285289

[19] Levin BR, Concepción-Acevedo J, Udekwu KI. 2014. Persistence: a copacetic and parsimonious hypothesis for the existence of non-inherited resistance to antibiotics. Curr Opin Microbiol 21:18–21. doi:10.1016/j.mib.2014.06.016 25090240 PMC4253300

[20] Dhawale A, Rath A. 2014. Antibiotic resistance: a threat and challenge to society. Ann Appl Biosci 1:R1–R6.

[21] Pereira CS, Thompson JA, Xavier KB. 2013. AI-2-mediated signalling in bacteria. FEMS Microbiol Rev 37:156–181. doi:10.1111/j.1574-6976.2012.00345.x 22712853

[22] Jiang K, Xu Y, Yuan B, Yue Y, Zhao M, Luo R, Wu H, Wang L, Zhang Y, Xiao J, Lin F. 2022. Effect of autoinducer-2 quorum sensing inhibitor on interspecies quorum sensing. Front Microbiol 13:791802. doi:10.3389/fmicb.2022.791802 35418956 PMC8996156

[23] Taga ME, Semmelhack JL, Bassler BL. 2001. The LuxS-dependent autoinducer AI-2 controls the expression of an ABC transporter that functions in AI-2 uptake in Salmonella typhimurium. Mol Microbiol 42:777–793. doi:10.1046/j.1365-2958.2001.02669.x 11722742

[24] Wang L, Hashimoto Y, Tsao C-Y, Valdes JJ, Bentley WE. 2005. Cyclic AMP (cAMP) and cAMP receptor protein influence both synthesis and uptake of extracellular autoinducer 2 in Escherichia coli. J Bacteriol 187:2066–2076. doi:10.1128/JB.187.6.2066-2076.2005 15743955 PMC1064054

[25] Xavier KB, Bassler BL. 2005. Regulation of uptake and processing of the quorum-sensing autoinducer AI-2 in Escherichia coli. J Bacteriol 187:238–248. doi:10.1128/JB.187.1.238-248.2005 15601708 PMC538819

[26] Khera R, Mehdipour AR, Bolla JR, Kahnt J, Welsch S, Ermler U, Muenke C, Robinson CV, Hummer G, Xie H, Michel H. 2022. Cryo‐EM structures of pentameric autoinducer‐2 exporter from Escherichia coli reveal its transport mechanism. EMBO J 41:e109990. doi:10.15252/embj.2021109990 35698912 PMC9475539

[27] Zuo J, Yin H, Hu J, Miao J, Chen Z, Qi K, Wang Z, Gong J, Phouthapane V, Jiang W, Mi R, Huang Y, Wang C, Han X. 2019. Lsr operon is associated with AI-2 transfer and pathogenicity in avian pathogenic Escherichia coli. Vet Res 50:109. doi:10.1186/s13567-019-0725-0 31831050 PMC6909531

[28] Marques JC, Oh IK, Ly DC, Lamosa P, Ventura MR, Miller ST, Xavier KB. 2014. LsrF, a coenzyme A-dependent thiolase, catalyzes the terminal step in processing the quorum sensing signal autoinducer-2. Proc Natl Acad Sci USA 111:14235–14240. doi:10.1073/pnas.1408691111 25225400 PMC4191781

[29] Feizi H, Alizadeh M, Azimi H, Khodadadi E, Kamounah FS, Ganbarov K, Ghotaslou R, Rezaee MA, Kafil HS. 2024. Induction of proteome changes involved in the cloning of mcr-1 and mcr-2 genes in Escherichia coli DH5-α strain to evaluate colistin resistance. J Glob Antimicrob Resist 36:151–159. doi:10.1016/j.jgar.2023.12.018 38154746

[30] De Araujo C, Balestrino D, Roth L, Charbonnel N, Forestier C. 2010. Quorum sensing affects biofilm formation through lipopolysaccharide synthesis in Klebsiella pneumoniae. Res Microbiol 161:595–603. doi:10.1016/j.resmic.2010.05.014 20600864

[31] Balestrino D, Haagensen JAJ, Rich C, Forestier C. 2005. Characterization of type 2 quorum sensing in Klebsiella pneumoniae and relationship with biofilm formation. J Bacteriol 187:2870–2880. doi:10.1128/JB.187.8.2870-2880.2005 15805533 PMC1070389

[32] Schubert S, Fischer D, Heesemann J. 1999. Ferric enterochelin transport in Yersinia enterocolitica: molecular and evolutionary aspects. J Bacteriol 181:6387–6395. doi:10.1128/JB.181.20.6387-6395.1999 10515929 PMC103774

[33] Chenault SS, Earhart CF. 1991. Organization of genes encoding membrane proteins of the Escherichia coli ferrienterobactin permease. Mol Microbiol 5:1405–1413. doi:10.1111/j.1365-2958.1991.tb00787.x 1787794

[34] Hantke K. 1990. Dihydroxybenzolyserine—a siderophore for E. coli. FEMS Microbiol Lett 55:5–8. doi:10.1016/0378-1097(90)90158-m 2139424

[35] Shea CM, McIntosh MA. 1991. Nucleotide sequence and genetic organization of the ferric enterobactin transport system: homology to other peripiasmic binding protein-dependent systems in Escherichia coli. Mol Microbiol 5:1415–1428. doi:10.1111/j.1365-2958.1991.tb00788.x 1838574

[36] Chu BCH, Otten R, Krewulak KD, Mulder FAA, Vogel HJ. 2014. The solution structure, binding properties, and dynamics of the bacterial siderophore-binding protein FepB. J Biol Chem 289:29219–29234. doi:10.1074/jbc.M114.564021 25173704 PMC4200274

[37] Futai M, Kimura H. 1977. Inducible membrane-bound L-lactate dehydrogenase from Escherichia coli. Purification and properties. J Biol Chem 252:5820–5827. doi:10.1016/S0021-9258(17)40096-2 18473

[38] Dong JM, Taylor JS, Latour DJ, Iuchi S, Lin EC. 1993. Three overlapping lct genes involved in L-lactate utilization by Escherichia coli. J Bacteriol 175:6671–6678. doi:10.1128/jb.175.20.6671-6678.1993 8407843 PMC206779

[39] Nishimura Y, Tan IKP, Ohgami Y, Kohgami K, Kamihara T. 1983. Induction of membrane-bound l-lactate dehydrogenase in Escherichia coli under conditions of nitrate respiration, fumarate reduction and trimethylamine-N-oxide reduction. FEMS Microbiol Lett 17:283–286. doi:10.1111/j.1574-6968.1983.tb00419.x

[40] Iuchi S, Aristarkhov A, Dong JM, Taylor JS, Lin EC. 1994. Effects of nitrate respiration on expression of the Arc-controlled operons encoding succinate dehydrogenase and flavin-linked L-lactate dehydrogenase. J Bacteriol 176:1695–1701. doi:10.1128/jb.176.6.1695-1701.1994 8132465 PMC205257

[41] Fu W, Yang F, Kang X, Zhang X, Li Y, Xia B, Jin C. 2007. First structure of the polymyxin resistance proteins. Biochem Biophys Res Commun 361:1033–1037. doi:10.1016/j.bbrc.2007.07.144 17686460

[42] Gunn JS. 2008. The Salmonella PmrAB regulon: lipopolysaccharide modifications, antimicrobial peptide resistance and more. Trends Microbiol 16:284–290. doi:10.1016/j.tim.2008.03.007 18467098

[43] Barchiesi J, Espariz M, Checa SK, Soncini FC. 2009. Downregulation of RpoN-controlled genes protects Salmonella cells from killing by the cationic antimicrobial peptide polymyxin B. FEMS Microbiol Lett 291:73–79. doi:10.1111/j.1574-6968.2008.01437.x 19076233

[44] Pilonieta MC, Erickson KD, Ernst RK, Detweiler CS. 2009. A protein important for antimicrobial peptide resistance, YdeI/OmdA, is in the periplasm and interacts with OmpD/NmpC. J Bacteriol 191:7243–7252. doi:10.1128/JB.00688-09 19767429 PMC2786563

[45] Nummila K, Kilpeläinen I, Zähringer U, Vaara M, Helander IM. 1995. Lipopolysaccharides of polymyxin B-resistant mutants of Escherichia coli are extensively substituted by 2-aminoethyl pyrophosphate and contain aminoarabinose in lipid A. Mol Microbiol 16:271–278. doi:10.1111/j.1365-2958.1995.tb02299.x 7565089

[46] Trent MS, Ribeiro AA, Doerrler WT, Lin S, Cotter RJ, Raetz CRH. 2001. Accumulation of a polyisoprene-linked amino sugar in polymyxin-resistant Salmonella typhimurium and Escherichia coli: structural characterization and transfer to lipid a in the periplasm. J Biol Chem 276:43132–43144. doi:10.1074/jbc.M106962200 11535605

[47] Kline T, Trent MS, Stead CM, Lee MS, Sousa MC, Felise HB, Nguyen HV, Miller SI. 2008. Synthesis of and evaluation of lipid A modification by 4-substituted 4-deoxy arabinose analogs as potential inhibitors of bacterial polymyxin resistance. Bioorg Med Chem Lett 18:1507–1510. doi:10.1016/j.bmcl.2007.12.061 18187325 PMC2516481

[48] Low H, Mukhamedova N, Capettini LDSA, Xia Y, Carmichael I, Cody SH, Huynh K, Ditiatkovski M, Ohkawa R, Bukrinsky M, Meikle PJ, Choi S-H, Field S, Miller YI, Sviridov D. 2020. Cholesterol efflux-independent modification of lipid rafts by AIBP (apolipoprotein A-I binding protein). Arterioscler Thromb Vasc Biol 40:2346–2359. doi:10.1161/ATVBAHA.120.315037 32787522 PMC7530101

[49] Chen Z, Liu Y, Jiang L, Zhang C, Qian X, Gu J, Song Z. 2024. Bacterial outer membrane vesicles increase polymyxin resistance in Pseudomonas aeruginosa while inhibiting its quorum sensing. J Hazard Mater 478:135588. doi:10.1016/j.jhazmat.2024.135588 39181004

[50] Payne SM, Neilands I. 1988. Iron and virulence in the family Enterobacteriaceae. Crit Rev Microbiol 16:81–111. doi:10.3109/10408418809104468 3067977

[51] Berner I, Greiner M, Metzger J, Jung G, Winkelmann G. 1991. Identification of enterobactin and linear dihydroxybenzoylserine compounds by HPLC and ion spray mass spectrometry (LC/MS and MS/MS). Biol Met 4:113–118. doi:10.1007/BF01135388 1831634

[52] Khan A, Singh P, Srivastava A. 2018. Synthesis, nature and utility of universal iron chelator – Siderophore: a review. Microbiol Res 212–213:103–111. doi:10.1016/j.micres.2017.10.012 29103733

[53] Raymond KN, Dertz EA, Kim SS. 2003. Enterobactin: an archetype for microbial iron transport. Proc Natl Acad Sci USA 100:3584–3588. doi:10.1073/pnas.0630018100 12655062 PMC152965

[54] Gehring AM, Bradley KA, Walsh CT. 1997. Enterobactin biosynthesis in Escherichia coli: isochorismate lyase (EntB) is a bifunctional enzyme that is phosphopantetheinylated by EntD and then acylated by EntE using ATP and 2,3-dihydroxybenzoate. Biochemistry 36:8495–8503. doi:10.1021/bi970453p 9214294

[55] Gadar K, de Dios R, Kadeřábková N, Prescott TAK, Mavridou DAI, McCarthy RR. 2023. Disrupting iron homeostasis can potentiate colistin activity and overcome colistin resistance mechanisms in Gram-negative bacteria. Commun Biol 6:937. doi:10.1038/s42003-023-05302-2 37704838 PMC10499790

[56] Pragasam AK, Shankar C, Veeraraghavan B, Biswas I, Nabarro LEB, Inbanathan FY, George B, Verghese S. 2016. Molecular mechanisms of colistin resistance in Klebsiella pneumoniae causing bacteremia from India—a first report. Front Microbiol 7:2135. doi:10.3389/fmicb.2016.02135 28119670 PMC5220082

[57] Kajiwara Y, Santander PJ, Roessner CA, Pérez LM, Scott AI. 2006. Genetically engineered synthesis and structural characterization of cobalt−precorrin 5A and −5B, two new intermediates on the anaerobic pathway to vitamin B_12_: definition of the roles of the CbiF and CbiG enzymes. J Am Chem Soc 128:9971–9978. doi:10.1021/ja062940a 16866557

[58] Moore SJ, Lawrence AD, Biedendieck R, Deery E, Frank S, Howard MJ, Rigby SEJ, Warren MJ. 2013. Elucidation of the anaerobic pathway for the corrin component of cobalamin (vitamin B_12_). Proc Natl Acad Sci USA 110:14906–14911. doi:10.1073/pnas.1308098110 23922391 PMC3773766

[59] Aulin LBS, Koumans CIM, Haakman Y, van Os W, Kraakman MEM, Gooskens J, Smits WK, Liakopoulos A, van Hasselt JGC. 2021. Distinct evolution of colistin resistance associated with experimental resistance evolution models in Klebsiella pneumoniae. J Antimicrob Chemother 76:533–535. doi:10.1093/jac/dkaa450 33150358 PMC7816166

[60] Janssen AB, Doorduijn DJ, Mills G, Rogers MRC, Bonten MJM, Rooijakkers SHM, Willems RJL, Bengoechea JA, van Schaik W. 2020. Evolution of colistin resistance in the Klebsiella pneumoniae complex follows multiple evolutionary trajectories with variable effects on fitness and virulence characteristics. Antimicrob Agents Chemother 65:e01958-20. doi:10.1128/AAC.01958-20 33139278 PMC7927857

[61] Grandclément C, Tannières M, Moréra S, Dessaux Y, Faure D. 2016. Quorum quenching: role in nature and applied developments. FEMS Microbiol Rev 40:86–116. doi:10.1093/femsre/fuv038 26432822

[62] Kyung SM, Lee JH, Lee E-S, Hwang C-Y, Yoo HS. 2023. Whole genome structure and resistance genes in carbapenemase-producing multidrug resistant ST378 Klebsiella pneumoniae. BMC Microbiol 23:323. doi:10.1186/s12866-023-03074-7 37924028 PMC10623767

[63] CLSI. 2017. Performance standards for antimicrobial susceptibility testing. Clinical and Laboratory Standards Institute, Wayne, PA.

[64] Friedman L, Alder JD, Silverman JA. 2006. Genetic changes that correlate with reduced susceptibility to daptomycin in Staphylococcus aureus. Antimicrob Agents Chemother 50:2137–2145. doi:10.1128/AAC.00039-06 16723576 PMC1479123

[65] Bray NL, Pimentel H, Melsted P, Pachter L. 2016. Near-optimal probabilistic RNA-seq quantification. Nat Biotechnol 34:525–527. doi:10.1038/nbt.3519 27043002

[66] Li B, Dewey CN. 2011. RSEM: accurate transcript quantification from RNA-Seq data with or without a reference genome. BMC Bioinformatics 12:1–16. doi:10.1186/1471-2105-12-323 21816040 PMC3163565

[67] McCarthy DJ, Chen Y, Smyth GK. 2012. Differential expression analysis of multifactor RNA-Seq experiments with respect to biological variation. Nucleic Acids Res 40:4288–4297. doi:10.1093/nar/gks042 22287627 PMC3378882

[68] Love MI, Huber W, Anders S. 2014. Moderated estimation of fold change and dispersion for RNA-seq data with DESeq2. Genome Biol 15:550. doi:10.1186/s13059-014-0550-8 25516281 PMC4302049

[69] Keseler IM, Gama-Castro S, Mackie A, Billington R, Bonavides-Martínez C, Caspi R, Kothari A, Krummenacker M, Midford PE, Muñiz-Rascado L, Ong WK, Paley S, Santos-Zavaleta A, Subhraveti P, Tierrafría VH, Wolfe AJ, Collado-Vides J, Paulsen IT, Karp PD. 2021. The EcoCyc database in 2021. Front Microbiol 12:711077. doi:10.3389/fmicb.2021.711077 34394059 PMC8357350

[70] Buchfink B, Xie C, Huson DH. 2015. Fast and sensitive protein alignment using DIAMOND. Nat Methods 12:59–60. doi:10.1038/nmeth.3176 25402007

[71] Huang DW, Sherman BT, Lempicki RA. 2009. Systematic and integrative analysis of large gene lists using DAVID bioinformatics resources. Nat Protoc 4:44–57. doi:10.1038/nprot.2008.211 19131956

[72] Sherman BT, Panzade G, Imamichi T, Chang W. 2024. DAVID Ortholog: an integrative tool to enhance functional analysis through orthologs. Bioinformatics 40:btae615. doi:10.1093/bioinformatics/btae615 39412445 PMC11520416

[73] Livak KJ, Schmittgen TD. 2001. Analysis of relative gene expression data using real-time quantitative PCR and the 2^−ΔΔC_T_^ method. Methods 25:402–408. doi:10.1006/meth.2001.1262 11846609

[74] McKenna A, Hanna M, Banks E, Sivachenko A, Cibulskis K, Kernytsky A, Garimella K, Altshuler D, Gabriel S, Daly M, DePristo MA. 2010. The Genome Analysis Toolkit: a MapReduce framework for analyzing next-generation DNA sequencing data. Genome Res 20:1297–1303. doi:10.1101/gr.107524.110 20644199 PMC2928508

[75] Garrison E, Marth G. 2016. Haplotype-based variant detection from short-read sequencing. arXiv. doi:10.48550/arXiv.1207.3907

[76] Li H, Handsaker B, Wysoker A, Fennell T, Ruan J, Homer N, Marth G, Abecasis G, Durbin R, 1000 Genome Project Data Processing Subgroup. 2009. The sequence alignment/map format and SAMtools. Bioinformatics 25:2078–2079. doi:10.1093/bioinformatics/btp352 19505943 PMC2723002

[77] Li H. 2011. A statistical framework for SNP calling, mutation discovery, association mapping and population genetical parameter estimation from sequencing data. Bioinformatics 27:2987–2993. doi:10.1093/bioinformatics/btr509 21903627 PMC3198575

[78] Kim S, Scheffler K, Halpern AL, Bekritsky MA, Noh E, Källberg M, Chen X, Kim Y, Beyter D, Krusche P, Saunders CT. 2018. Strelka2: fast and accurate calling of germline and somatic variants. Nat Methods 15:591–594. doi:10.1038/s41592-018-0051-x 30013048

[79] Niu B, Ye K, Zhang Q, Lu C, Xie M, McLellan MD, Wendl MC, Ding L. 2014. MSIsensor: microsatellite instability detection using paired tumor-normal sequence data. Bioinformatics 30:1015–1016. doi:10.1093/bioinformatics/btt755 24371154 PMC3967115

[80] Boeva V, Popova T, Bleakley K, Chiche P, Cappo J, Schleiermacher G, Janoueix-Lerosey I, Delattre O, Barillot E. 2012. Control-FREEC: a tool for assessing copy number and allelic content using next-generation sequencing data. Bioinformatics 28:423–425. doi:10.1093/bioinformatics/btr670 22155870 PMC3268243

[81] Chen X, Schulz-Trieglaff O, Shaw R, Barnes B, Schlesinger F, Källberg M, Cox AJ, Kruglyak S, Saunders CT. 2016. Manta: rapid detection of structural variants and indels for germline and cancer sequencing applications. Bioinformatics 32:1220–1222. doi:10.1093/bioinformatics/btv710 26647377

[82] Van Loo P, Nordgard SH, Lingjærde OC, Russnes HG, Rye IH, Sun W, Weigman VJ, Marynen P, Zetterberg A, Naume B, Perou CM, Børresen-Dale A-L, Kristensen VN. 2010. Allele-specific copy number analysis of tumors. Proc Natl Acad Sci USA 107:16910–16915. doi:10.1073/pnas.1009843107 20837533 PMC2947907

[83] Cingolani P, Platts A, Wang LL, Coon M, Nguyen T, Wang L, Land SJ, Lu X, Ruden DM. 2012. A program for annotating and predicting the effects of single nucleotide polymorphisms, SnpEff: SNPs in the genome of Drosophila melanogaster strain w^1118^; iso-2; iso-3. Fly (Austin) 6:80–92. doi:10.4161/fly.19695 22728672 PMC3679285

[84] Andrews S. 2010. FastQC: a quality control tool for high throughput sequence data. Available from: http://www.bioinformatics.babraham.ac.uk/projects/fastqc

[85] Ewels P, Magnusson M, Lundin S, Käller M. 2016. MultiQC: summarize analysis results for multiple tools and samples in a single report. Bioinformatics 32:3047–3048. doi:10.1093/bioinformatics/btw354 27312411 PMC5039924

[86] Danecek P, Bonfield JK, Liddle J, Marshall J, Ohan V, Pollard MO, Whitwham A, Keane T, McCarthy SA, Davies RM, Li H. 2021. Twelve years of SAMtools and BCFtools. Gigascience 10:giab008. doi:10.1093/gigascience/giab008 33590861 PMC7931819

[87] Talevich E, Shain AH, Botton T, Bastian BC. 2016. CNVkit: genome-wide copy number detection and visualization from targeted DNA sequencing. PLoS Comput Biol 12:e1004873. doi:10.1371/journal.pcbi.1004873 27100738 PMC4839673

[88] Okonechnikov K, Conesa A, García-Alcalde F. 2016. Qualimap 2: advanced multi-sample quality control for high-throughput sequencing data. Bioinformatics 32:292–294. doi:10.1093/bioinformatics/btv566 26428292 PMC4708105

[89] Danecek P, Auton A, Abecasis G, Albers CA, Banks E, DePristo MA, Handsaker RE, Lunter G, Marth GT, Sherry ST, McVean G, Durbin R, 1000 Genomes Project Analysis Group. 2011. The variant call format and VCFtools. Bioinformatics 27:2156–2158. doi:10.1093/bioinformatics/btr330 21653522 PMC3137218

[90] Stothard P, Wishart DS. 2005. Circular genome visualization and exploration using CGView. Bioinformatics 21:537–539. doi:10.1093/bioinformatics/bti054 15479716

