# Supplementary material for: Delineating molecular mechanisms on the acquisition of in vitro-adapted colistin-resistant Klebsiella pneumoniae by transcriptomic analysis
Source: Microbiol Spectr. 2025 Oct 21;13(12):e03428-24. doi: 10.1128/spectrum.03428-24 (PMC12671167; doi:10.1128/spectrum.03428-24)
Supplement: Fig. S9 — Original data complementing Fig. 2B. [file spectrum.03428-24-s0001.pdf]

Supplmentary Figure. Original images of the colonies inserted in the Figure 2B.

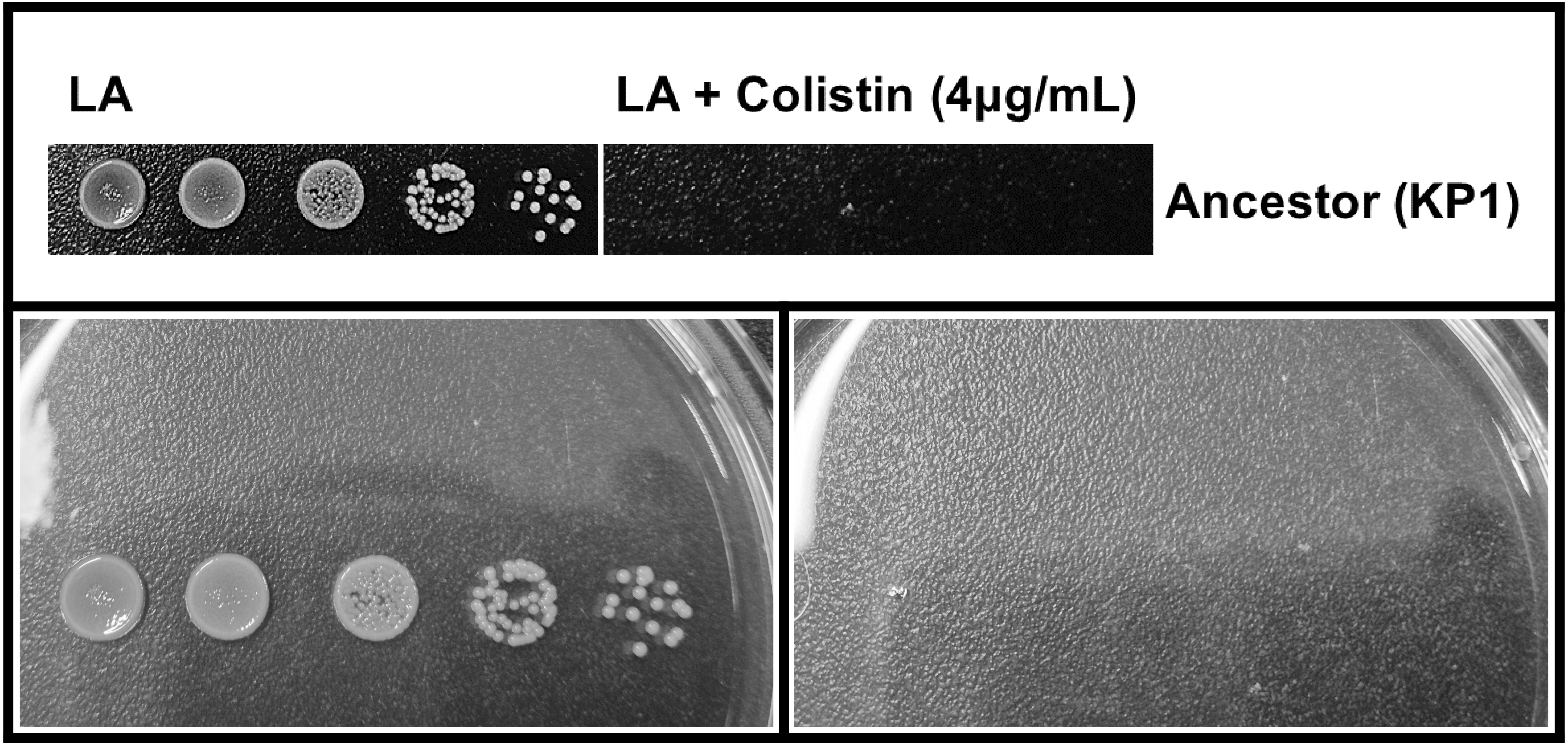

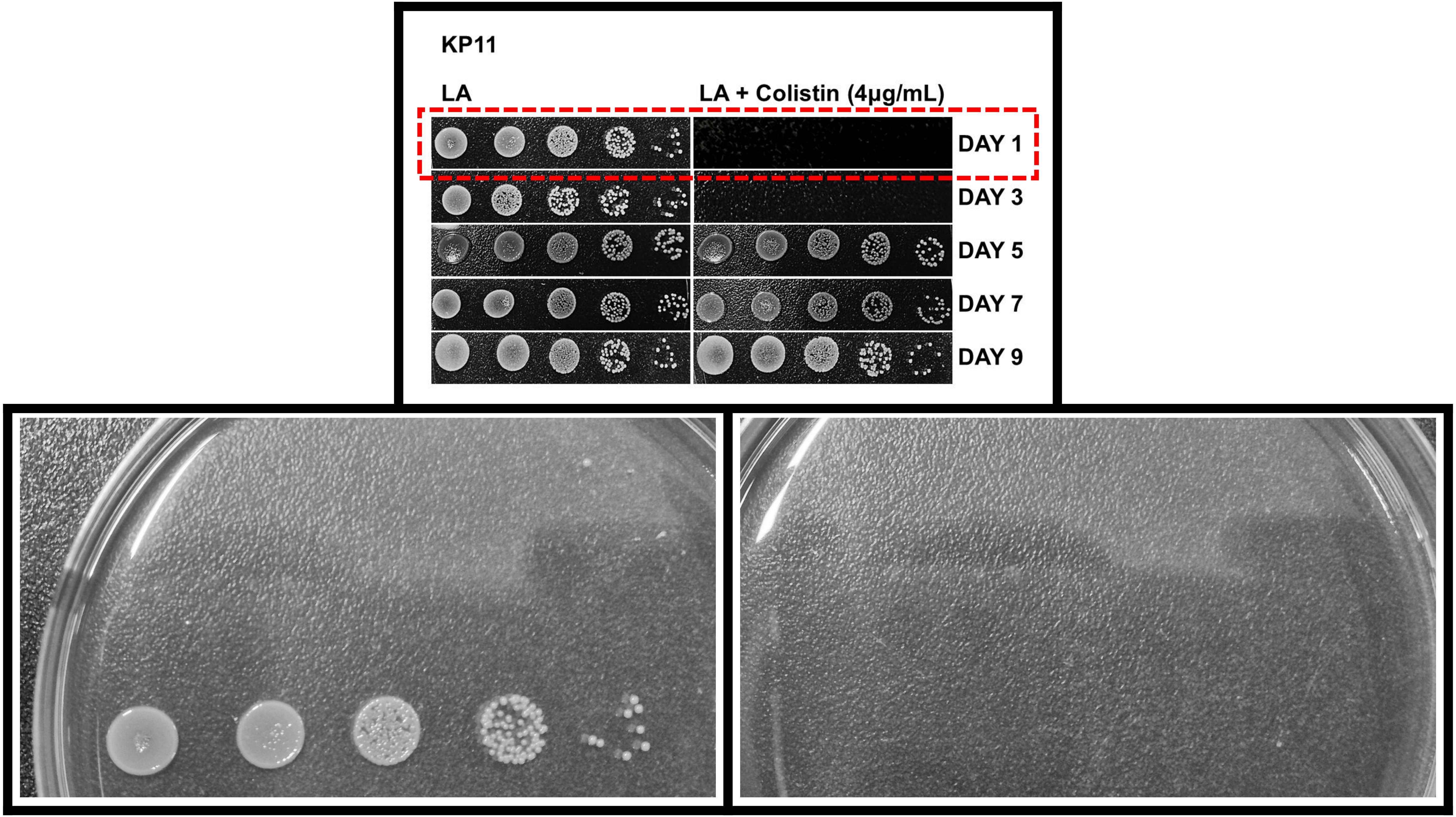
KP11
LA
LA + Colistin (4μg/mL)
DAY 1
DAY 3
DAY 5
DAY 7
DAY 9

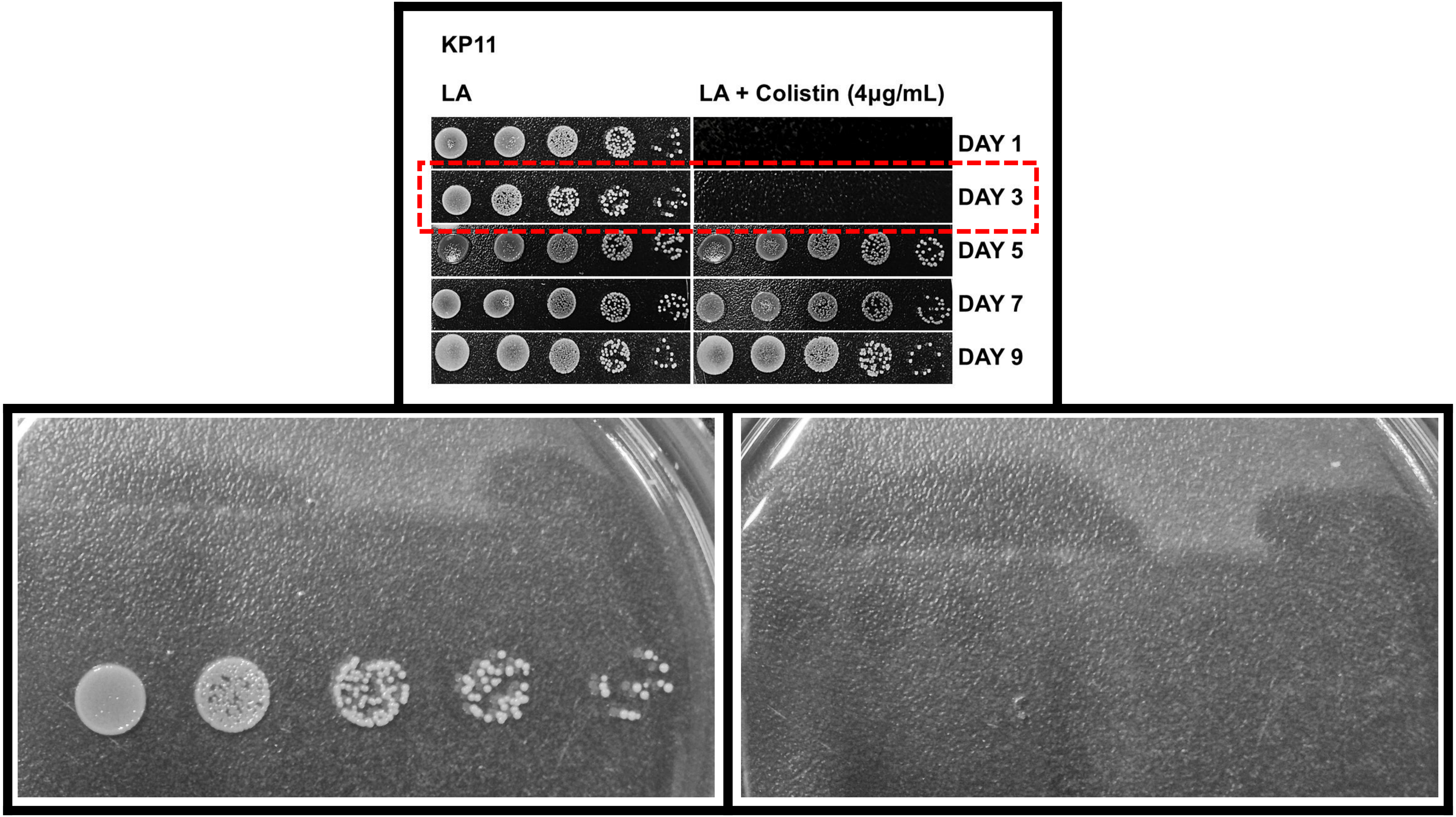
KP11
LA
LA + Colistin (4μg/mL)
DAY 1
DAY 3
DAY 5
DAY 7
DAY 9

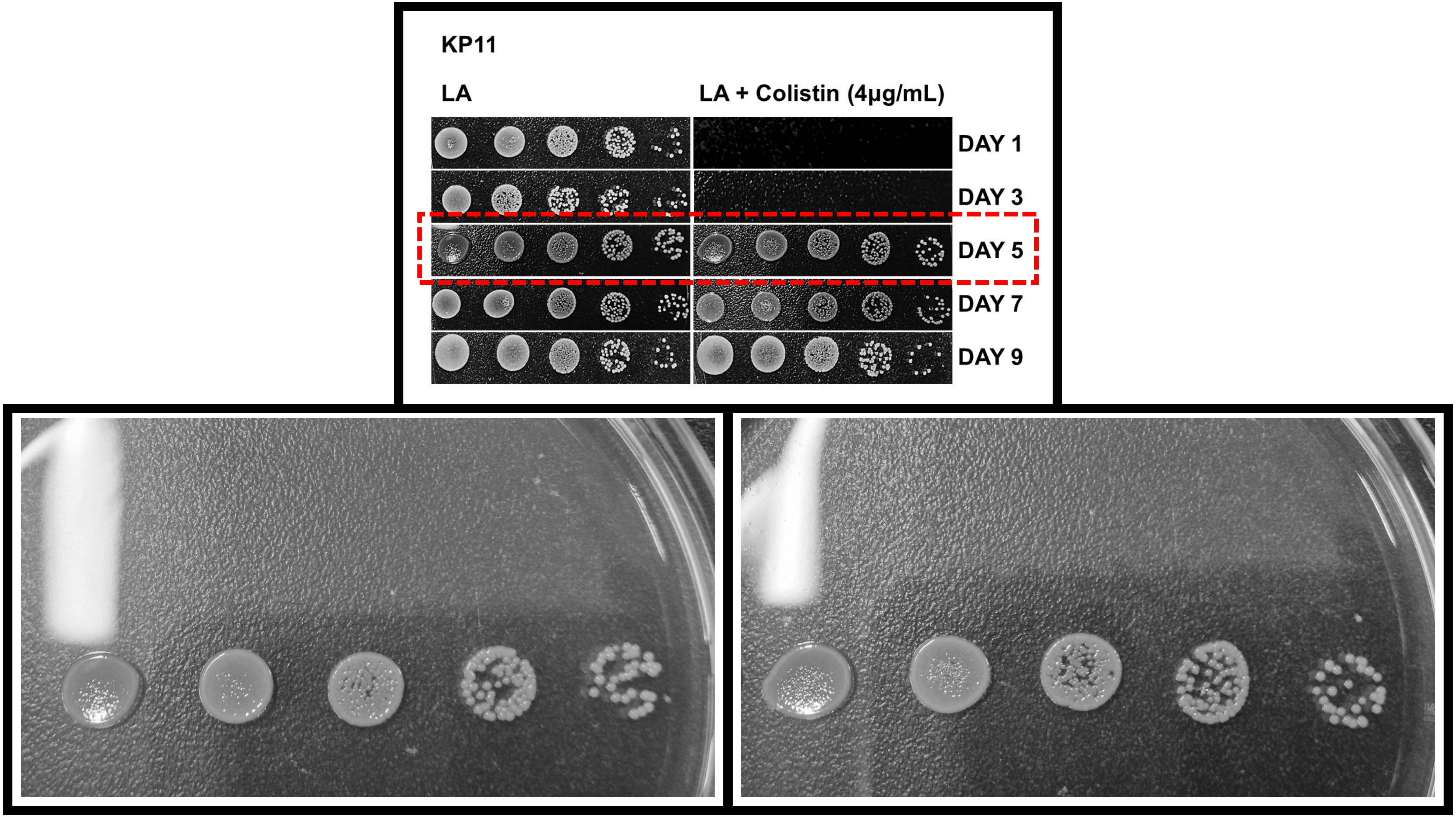
KP11
LA
LA + Colistin (4μg/mL)
DAY 1
DAY 3
DAY 5
DAY 7
DAY 9

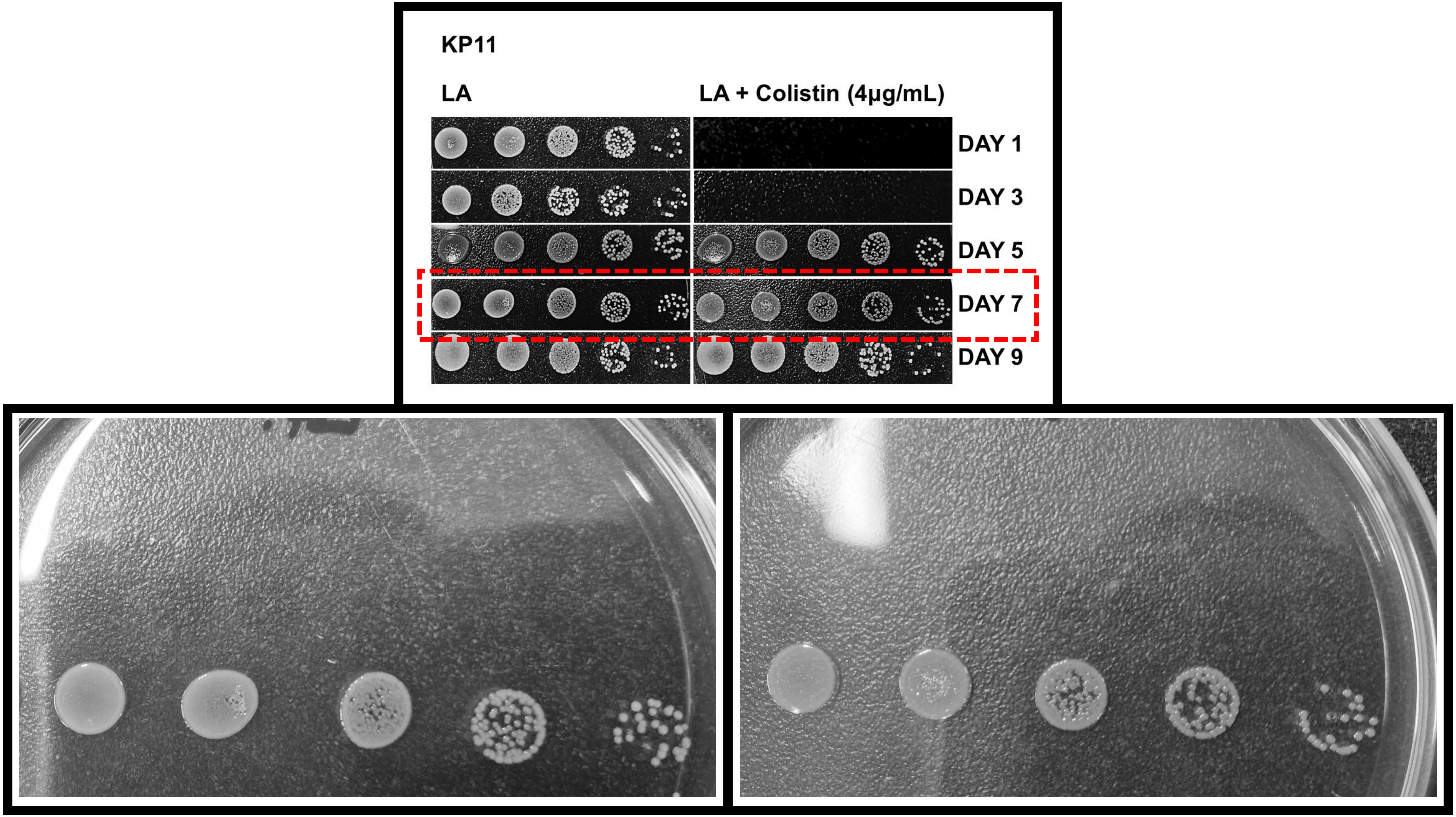
KP11
LA
LA + Colistin (4μg/mL)
DAY 1
DAY 3
DAY 5
DAY 7
DAY 9

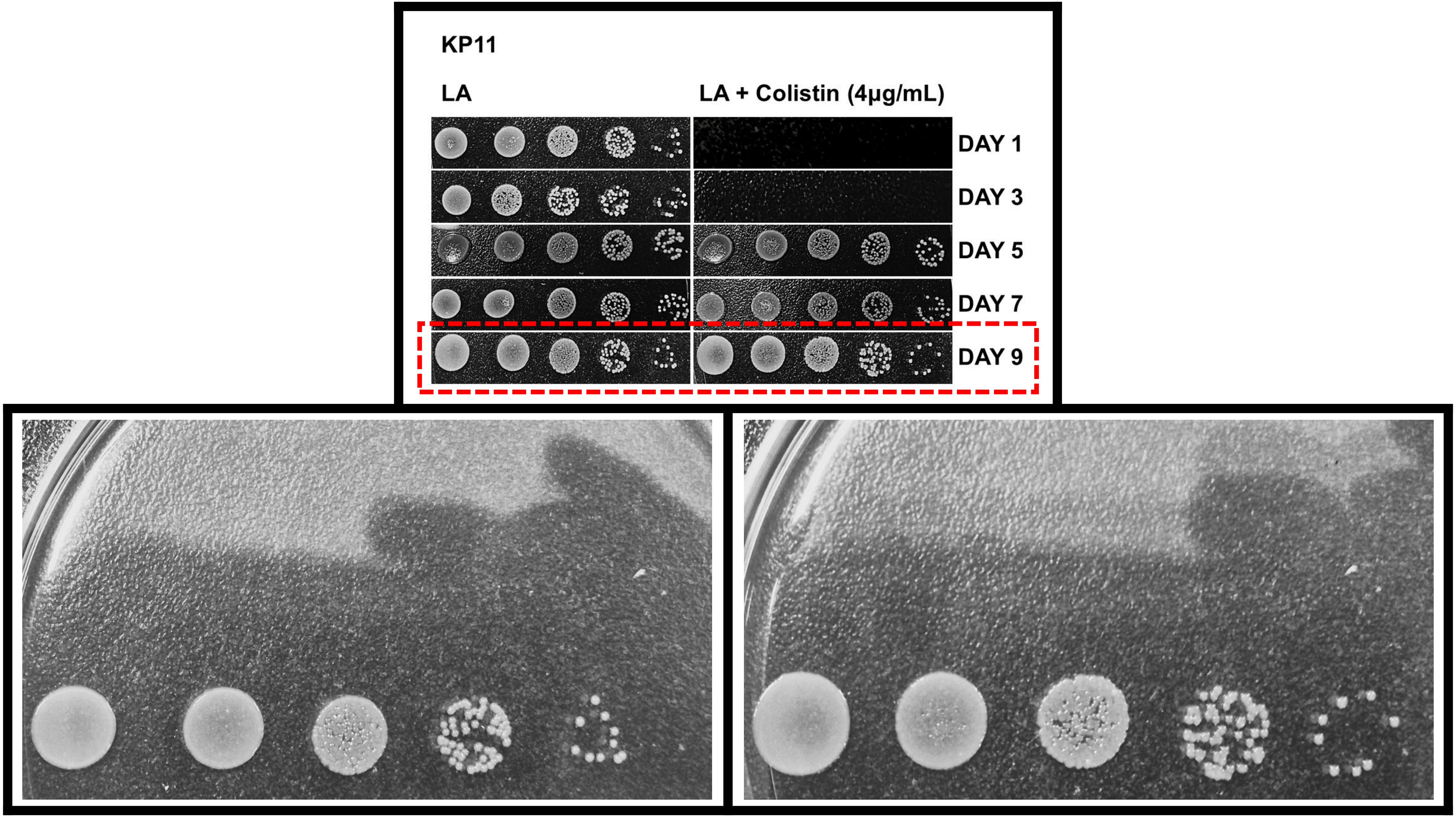
KP11
LA
LA + Colistin (4μg/mL)
DAY 1
DAY 3
DAY 5
DAY 7
DAY 9

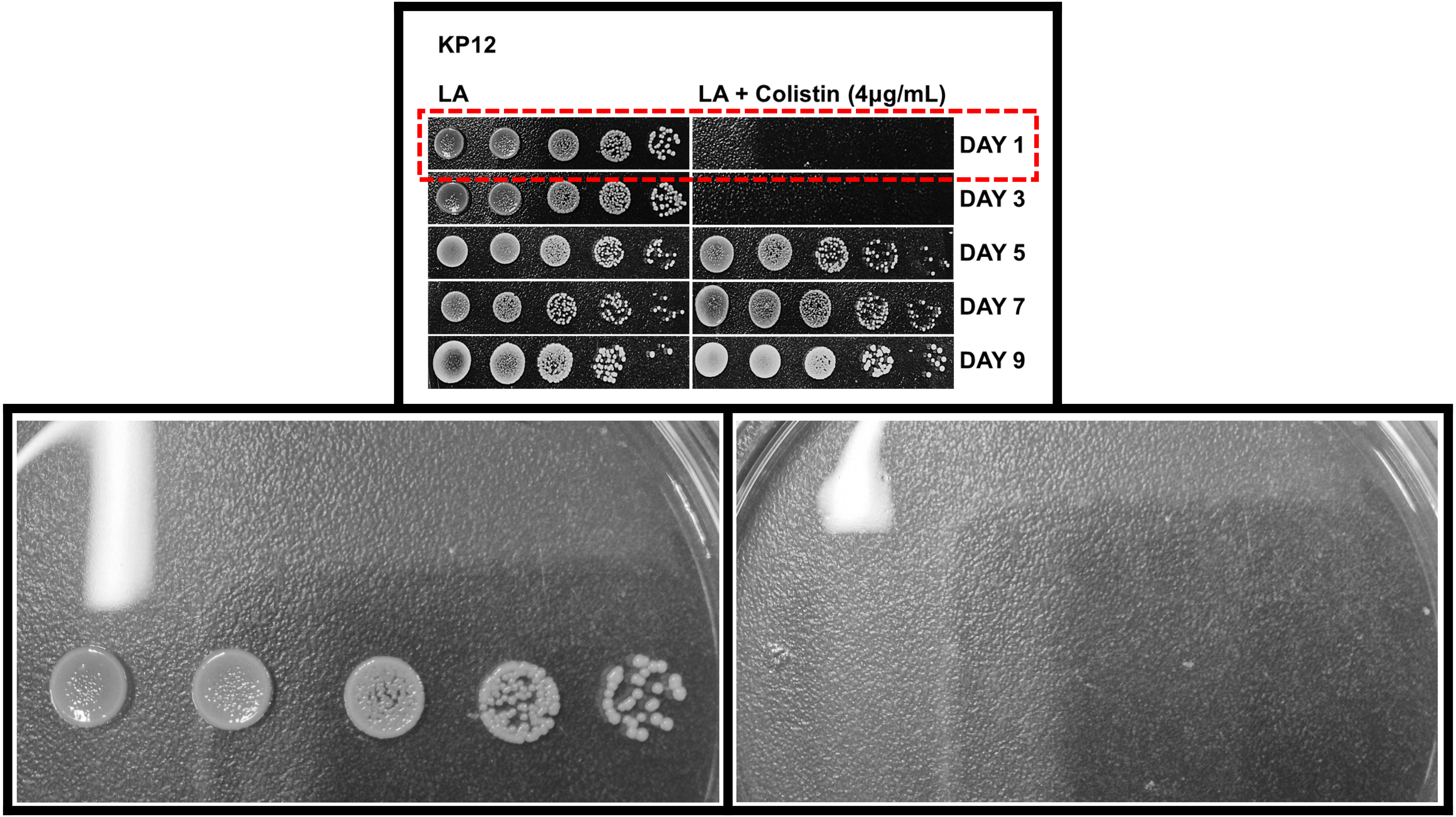
KP12
LA
LA + Colistin (4μg/mL)
DAY 1
DAY 3
DAY 5
DAY 7
DAY 9

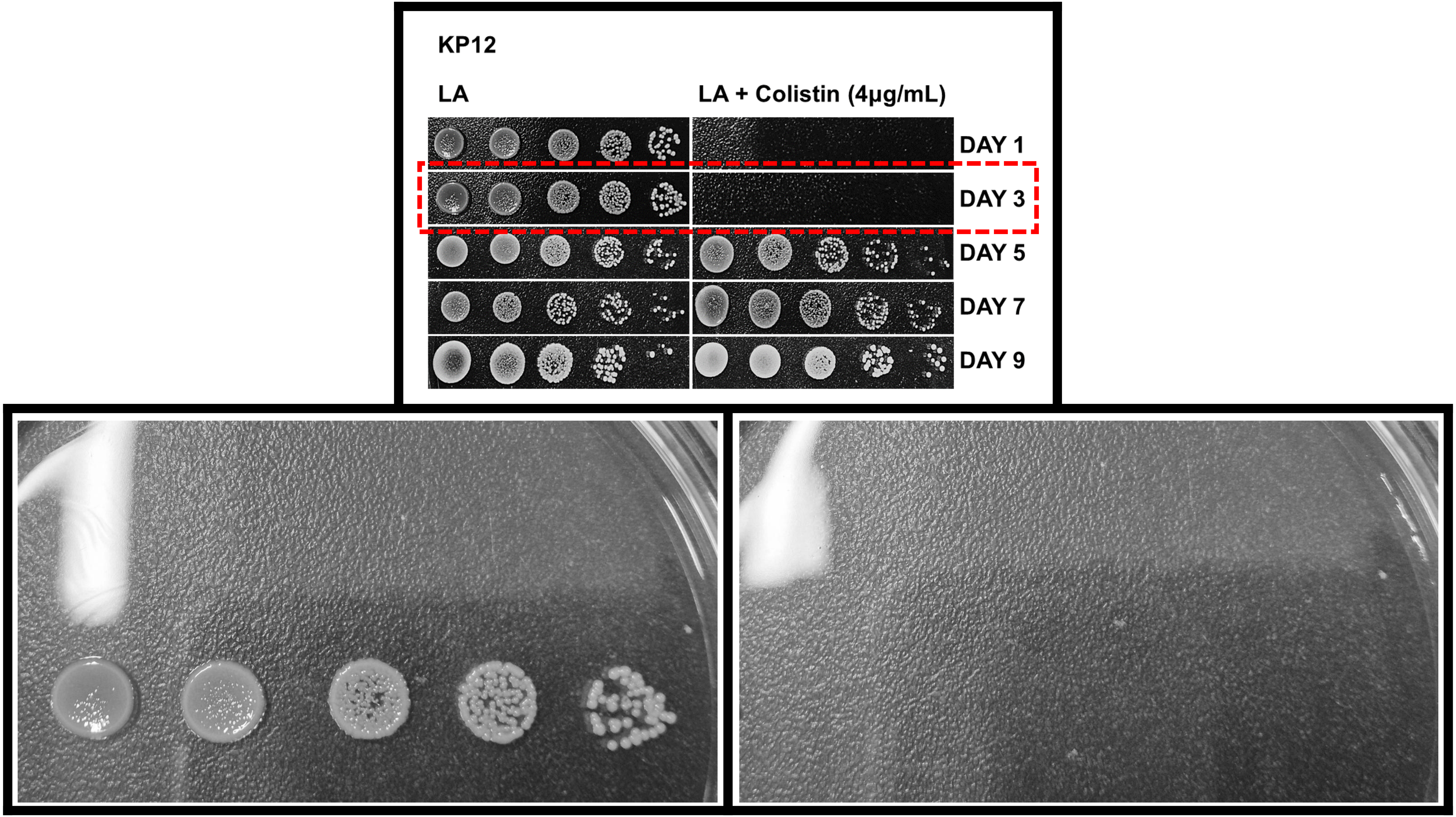
KP12
LA
LA + Colistin (4µg/mL)
DAY 1
DAY 3
DAY 5
DAY 7
DAY 9

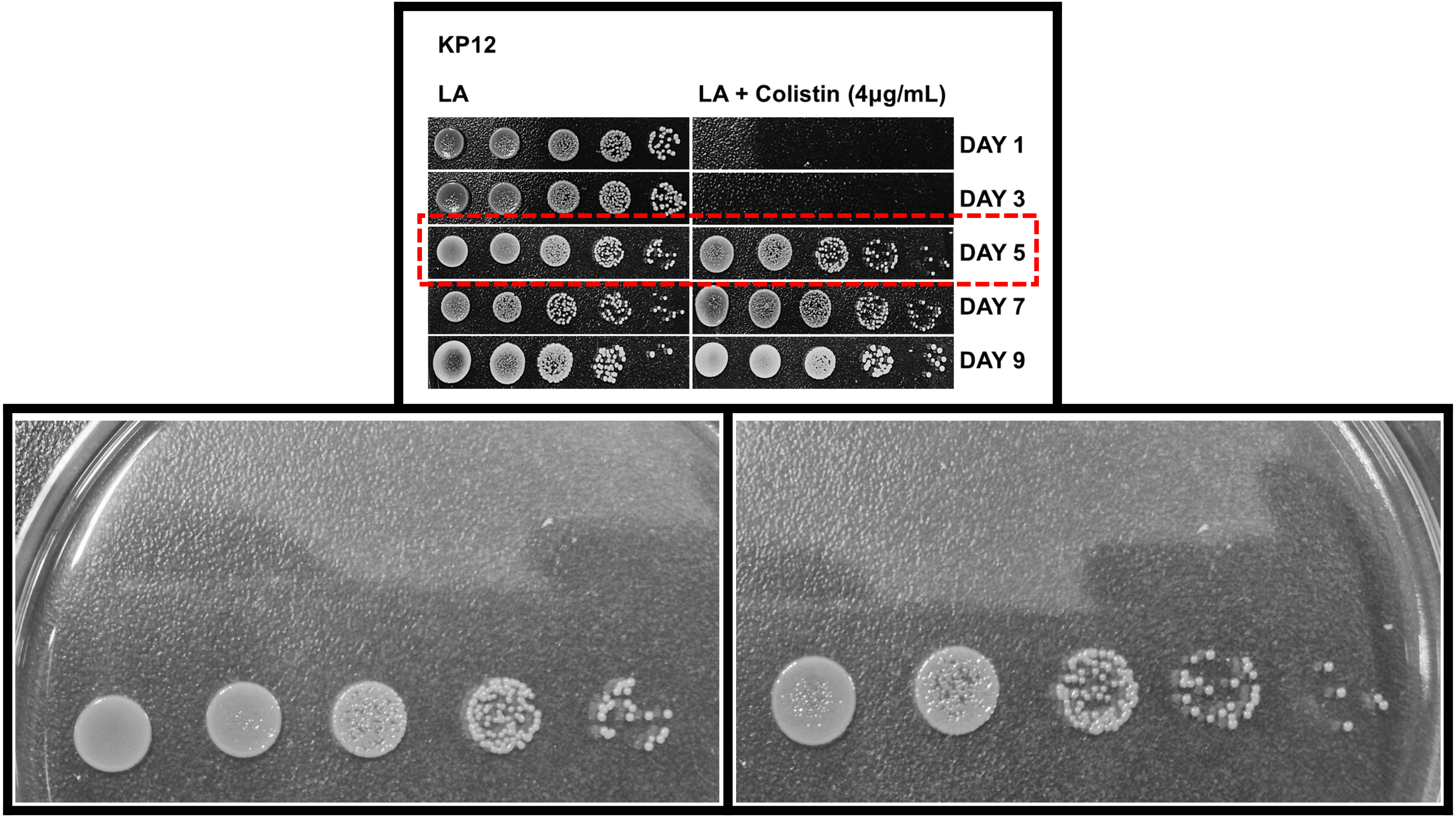
KP12
LA
LA + Colistin (4μg/mL)
DAY 1
DAY 3
DAY 5
DAY 7
DAY 9

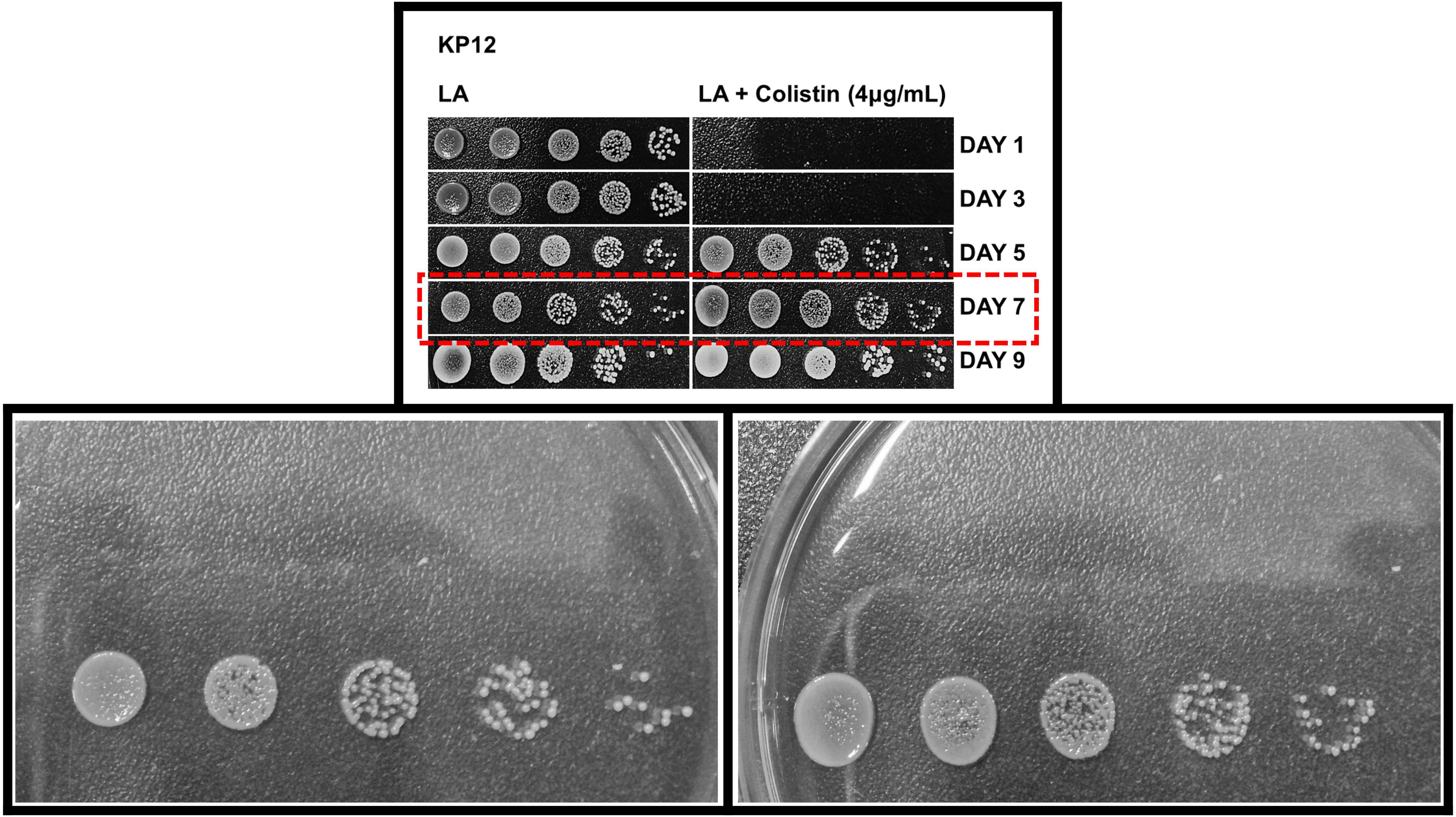
KP12
LA
LA + Colistin (4μg/mL)
DAY 1
DAY 3
DAY 5
DAY 7
DAY 9

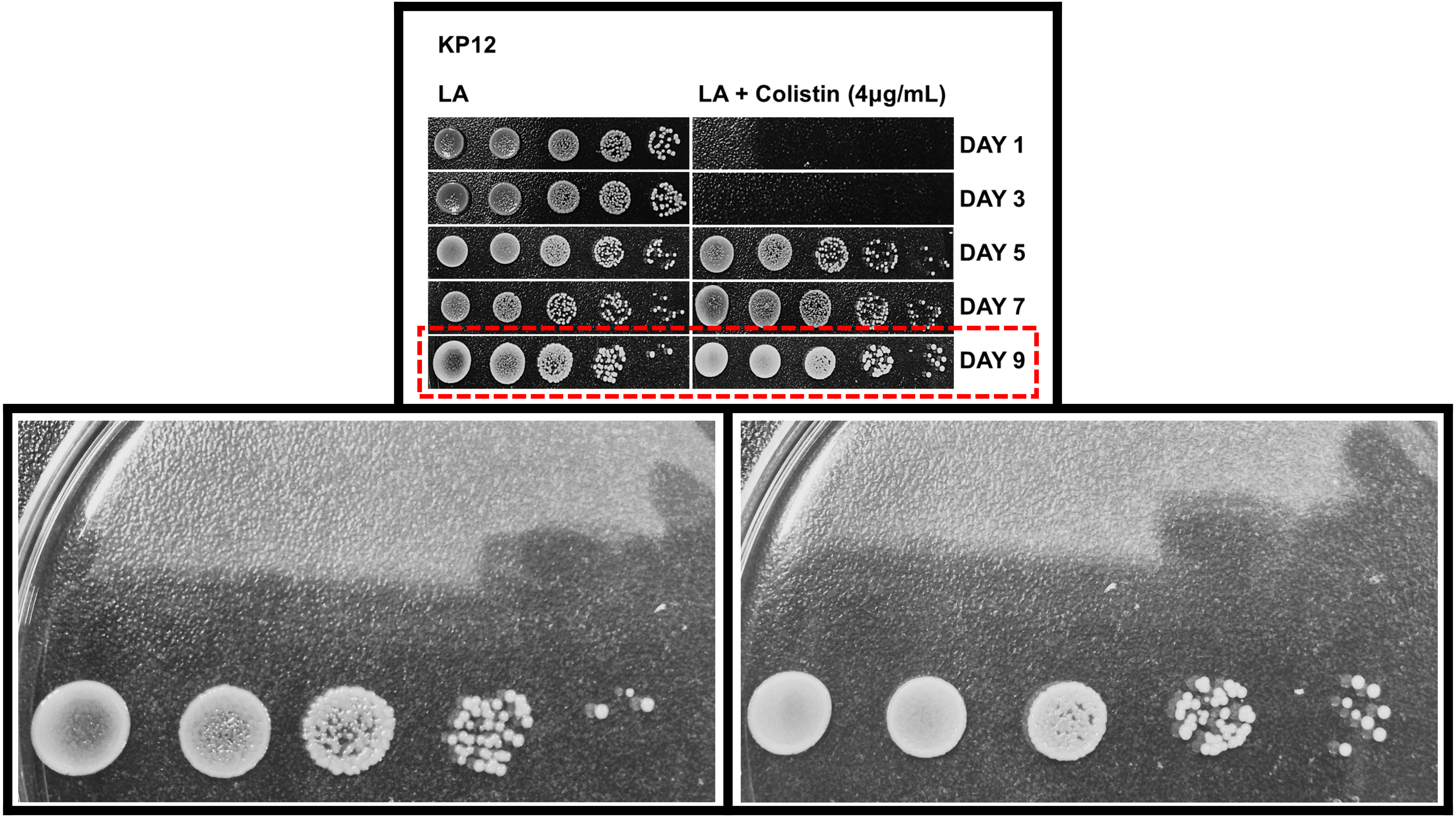
KP12
LA
LA + Colistin (4µg/mL)
DAY 1
DAY 3
DAY 5
DAY 7
DAY 9

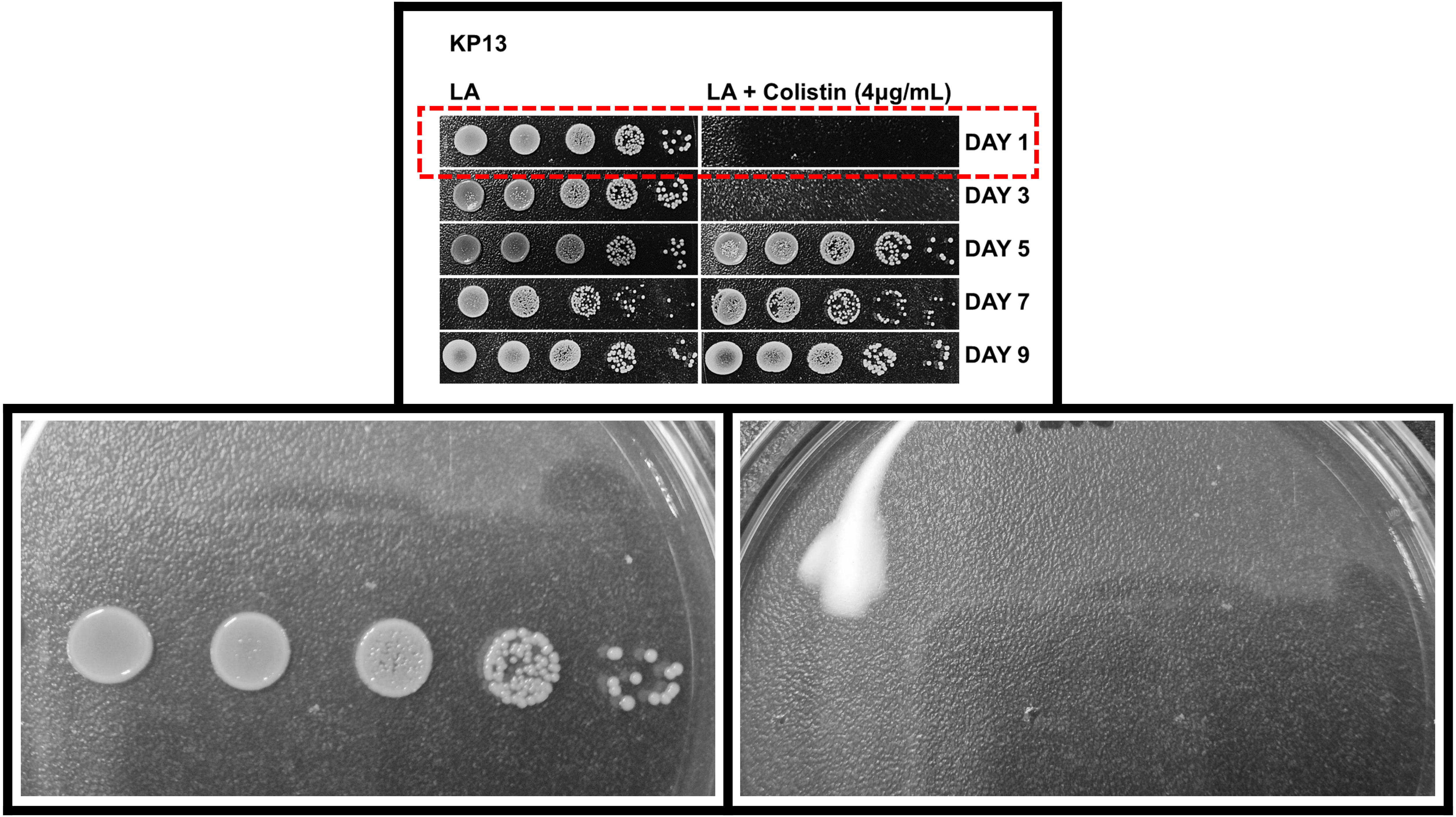
KP13
LA
LA + Colistin (4μg/mL)
DAY 1
DAY 3
DAY 5
DAY 7
DAY 9

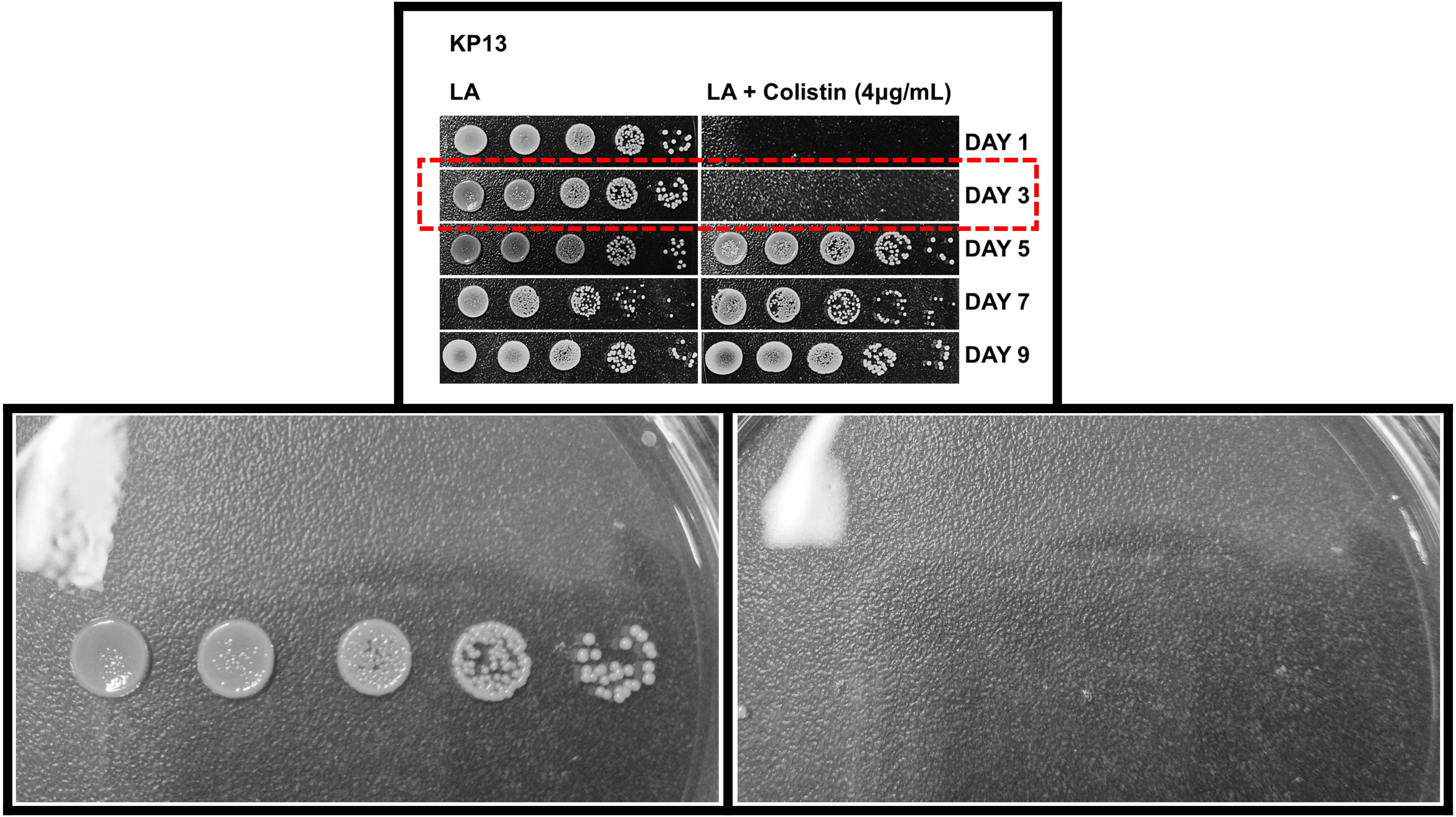
KP13
LA
LA + Colistin (4µg/mL)
DAY 1
DAY 3
DAY 5
DAY 7
DAY 9

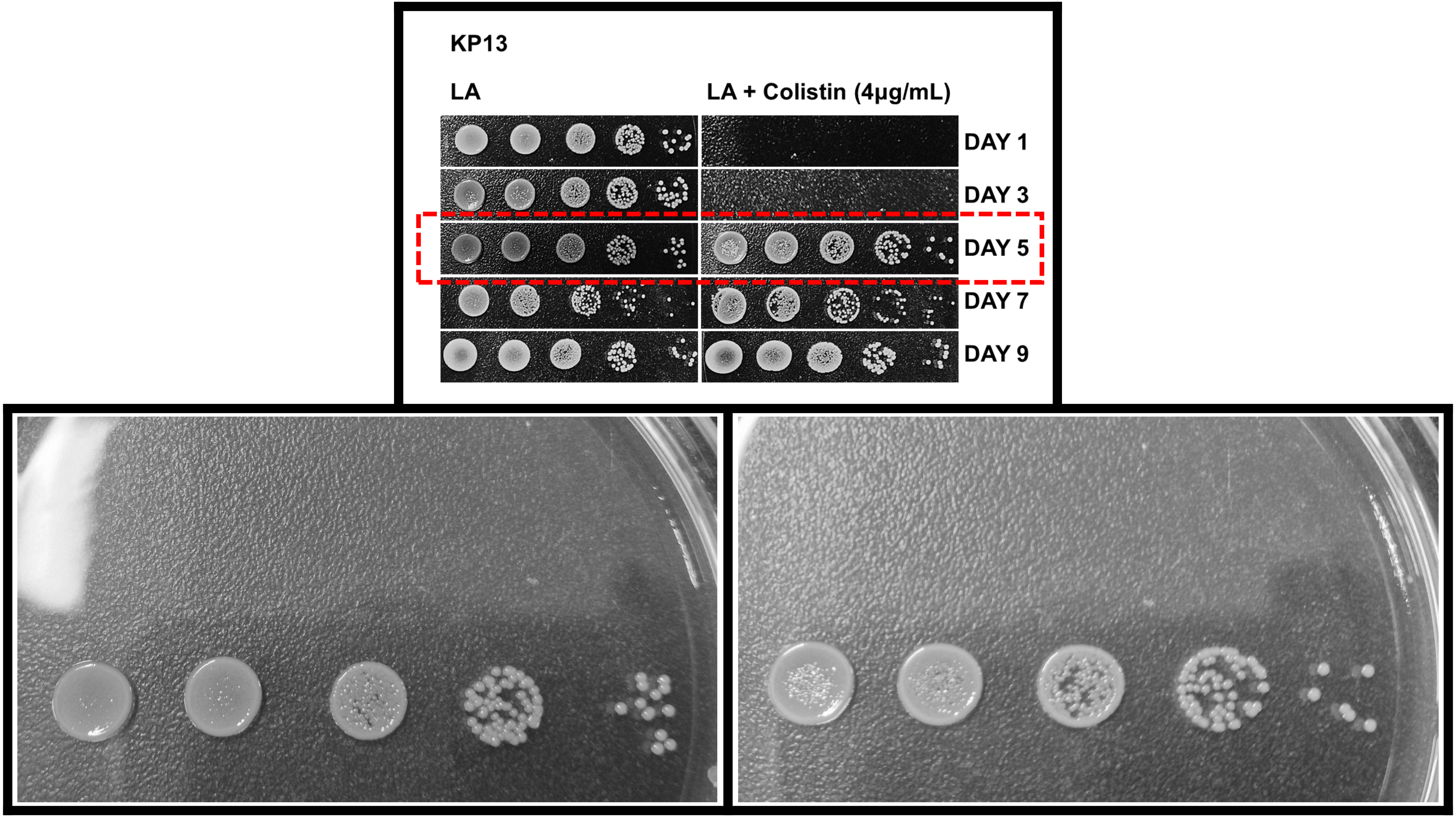
KP13
LA
LA + Colistin (4μg/mL)
DAY 1
DAY 3
DAY 5
DAY 7
DAY 9

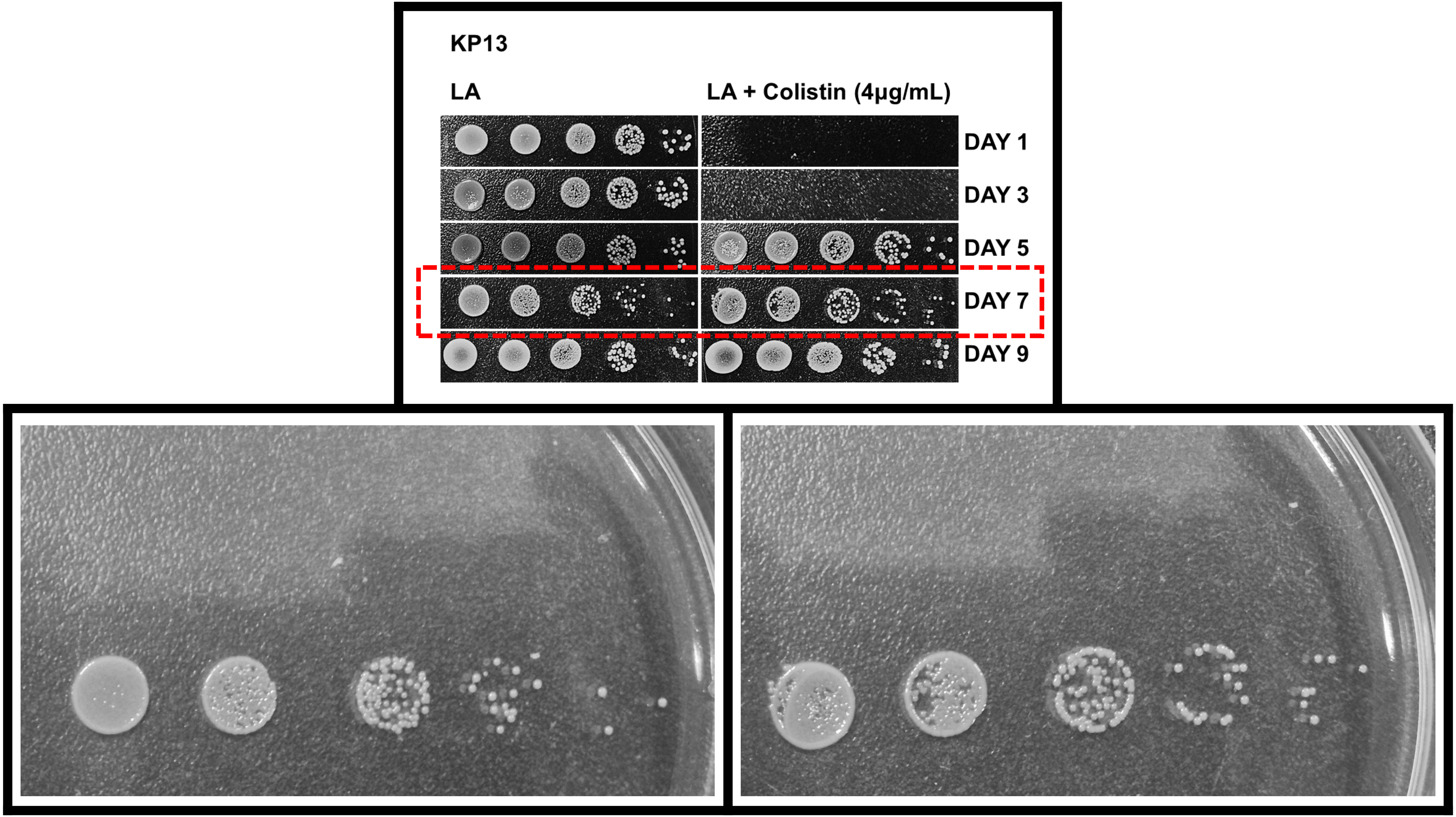
KP13
LA
LA + Colistin (4μg/mL)
DAY 1
DAY 3
DAY 5
DAY 7
DAY 9

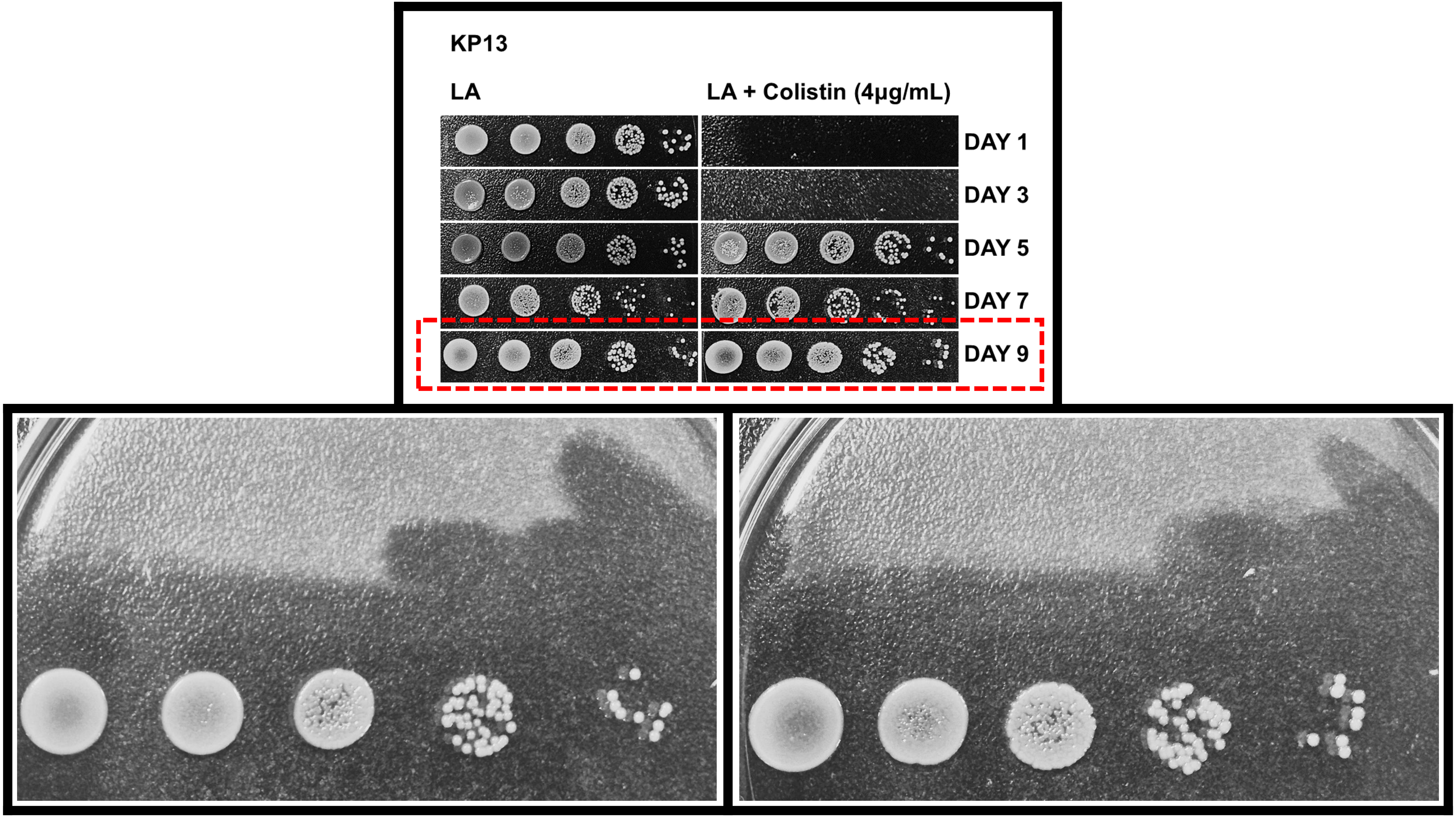
KP13
LA
LA + Colistin (4µg/mL)
DAY 1
DAY 3
DAY 5
DAY 7
DAY 9

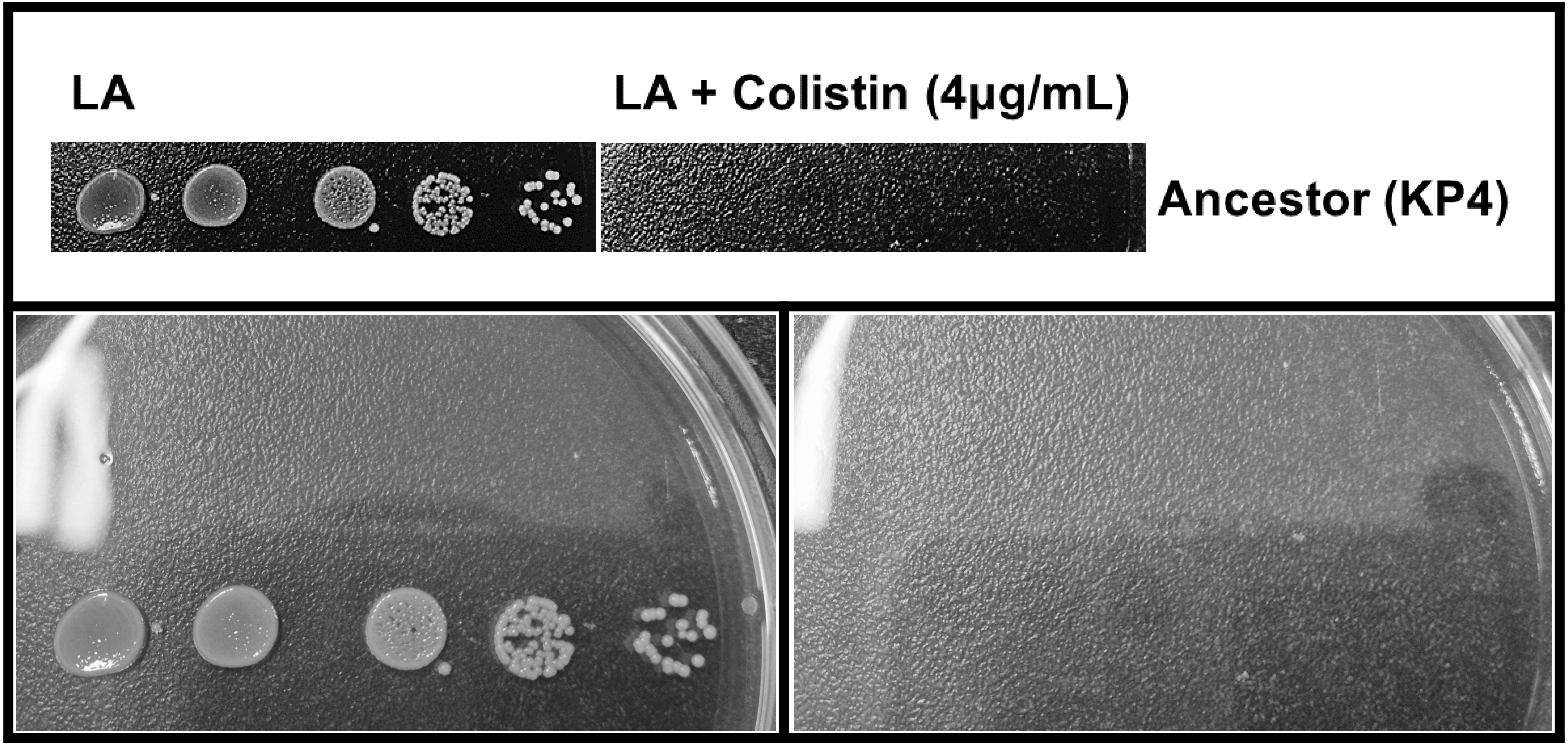
LA
LA + Colistin (4μg/mL)
Ancestor (KP4)

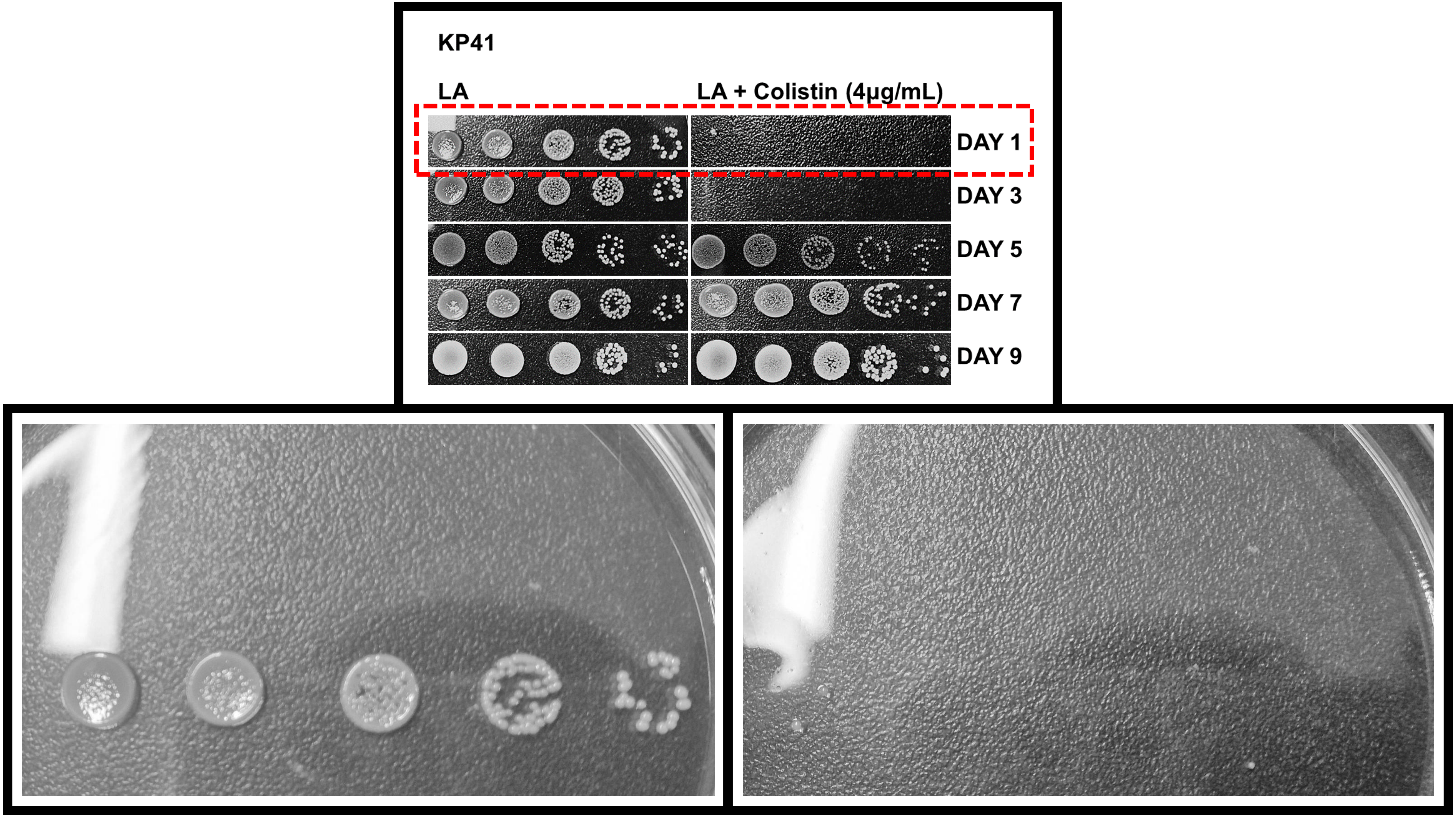
KP41
LA
LA + Colistin (4μg/mL)
DAY 1
DAY 3
DAY 5
DAY 7
DAY 9

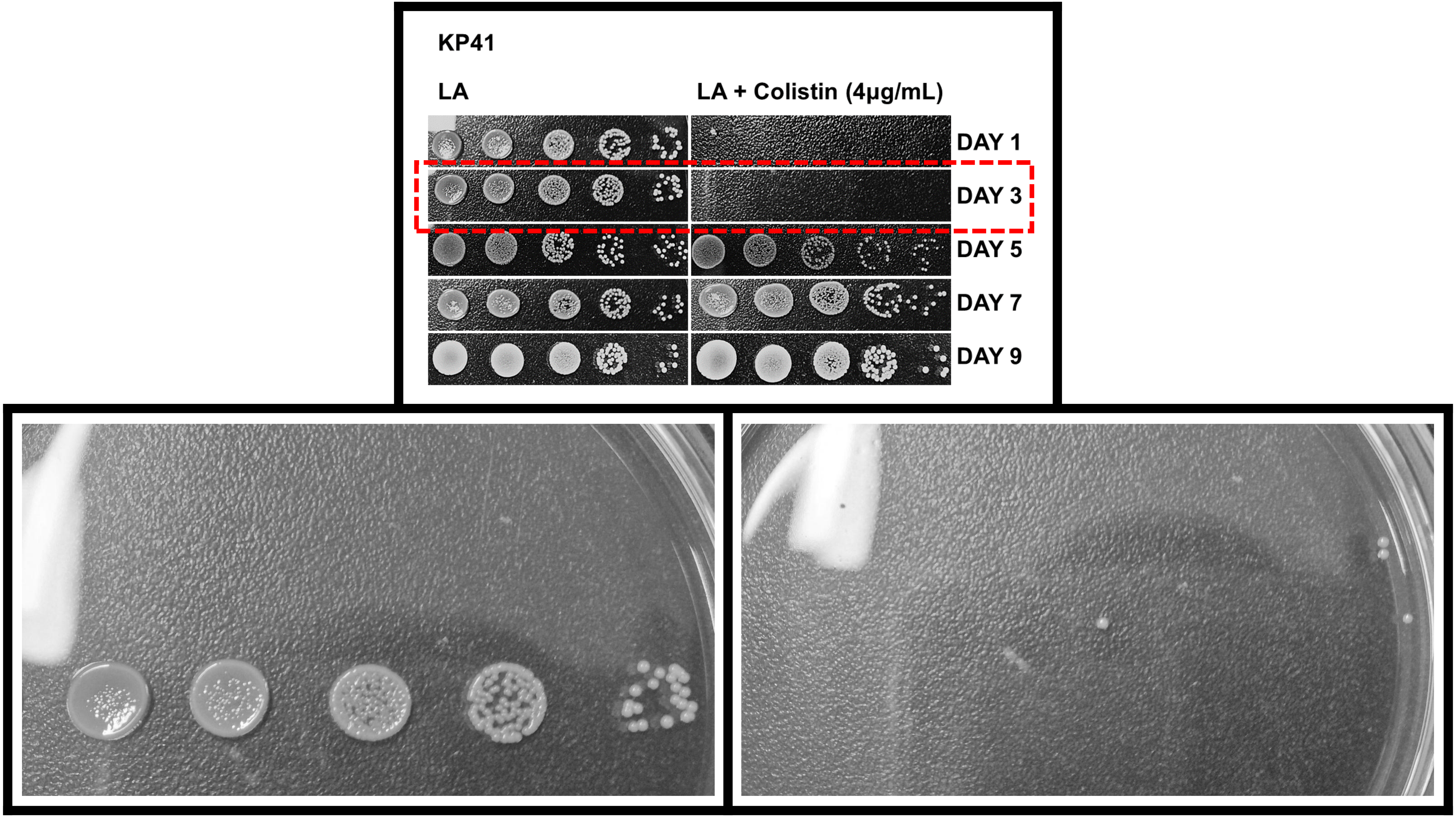
KP41
LA
LA + Colistin (4μg/mL)
DAY 1
DAY 3
DAY 5
DAY 7
DAY 9

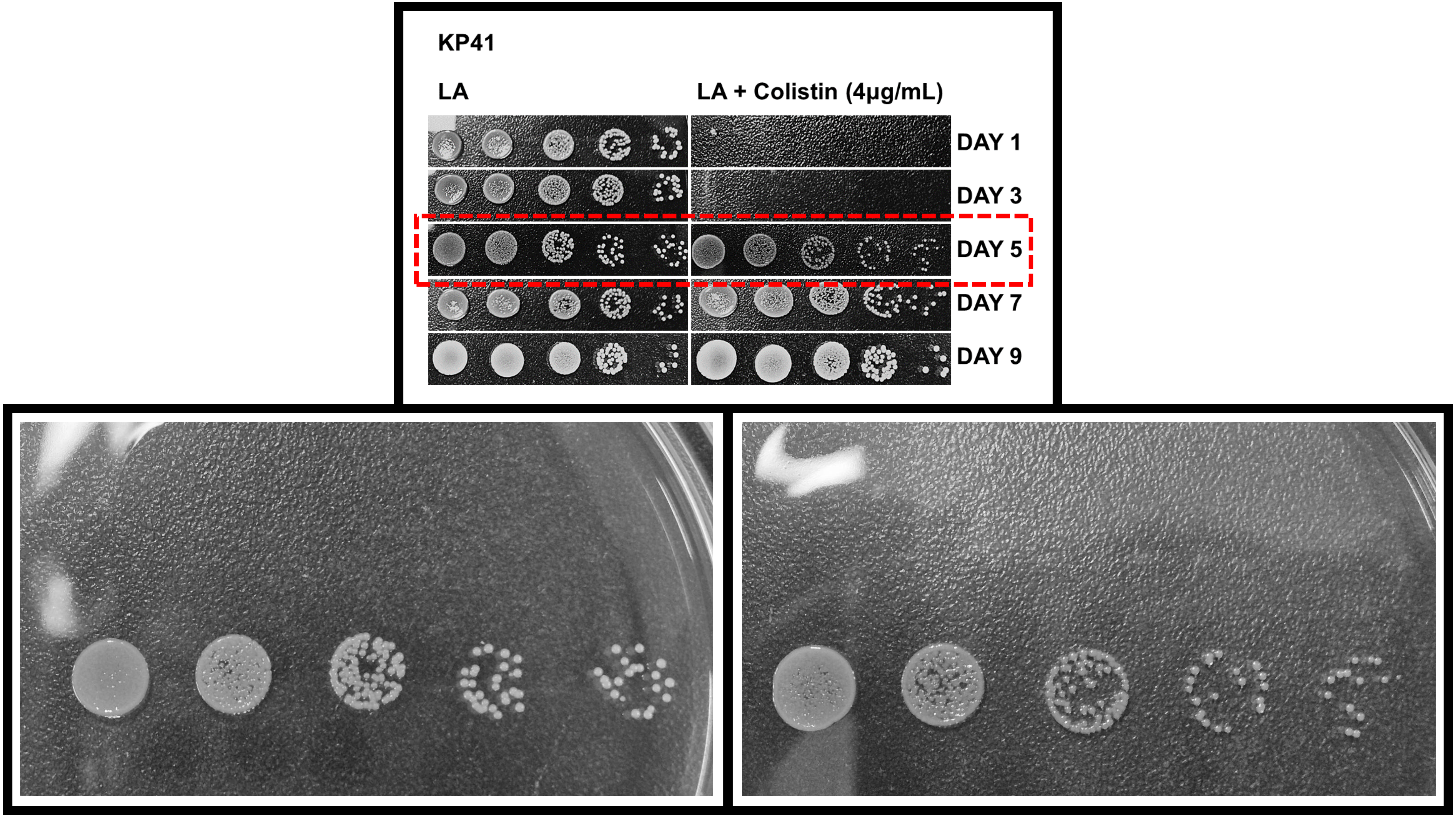
KP41
LA
LA + Colistin (4μg/mL)
DAY 1
DAY 3
DAY 5
DAY 7
DAY 9

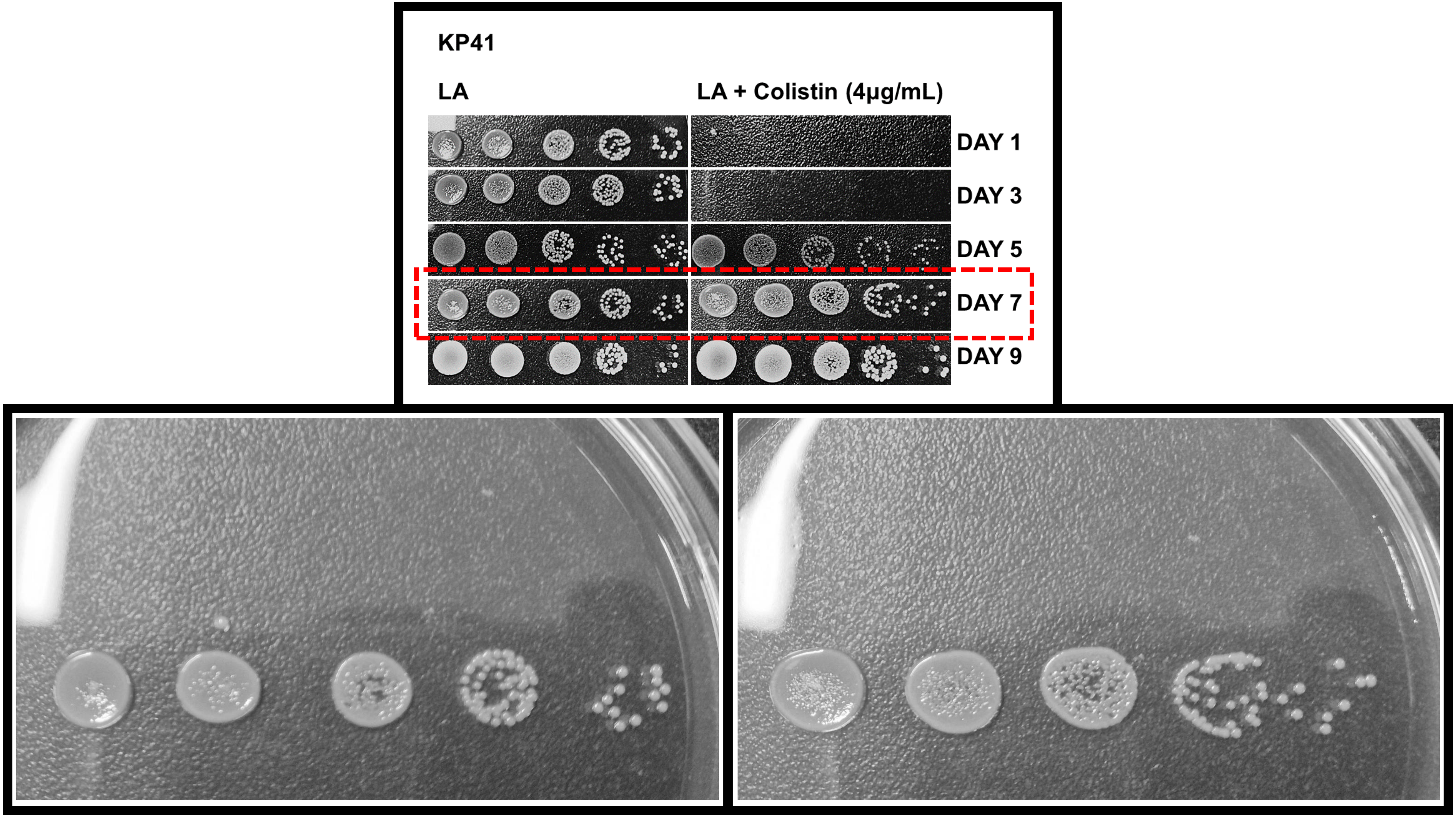
KP41
LA
LA + Colistin (4μg/mL)
DAY 1
DAY 3
DAY 5
DAY 7
DAY 9

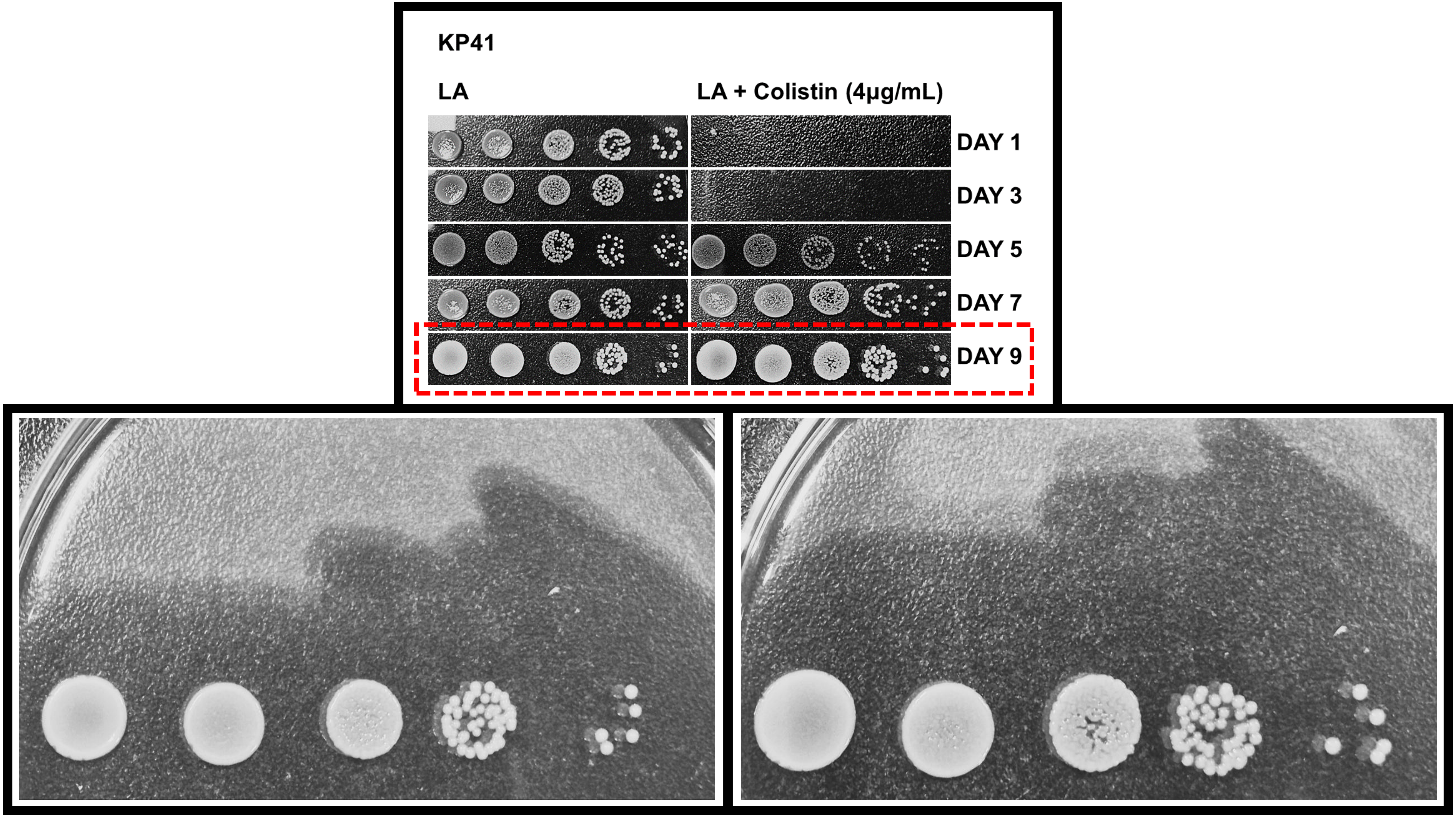

KP41
LA
LA + Colistin (4μg/mL)
DAY 1
DAY 3
DAY 5
DAY 7
DAY 9

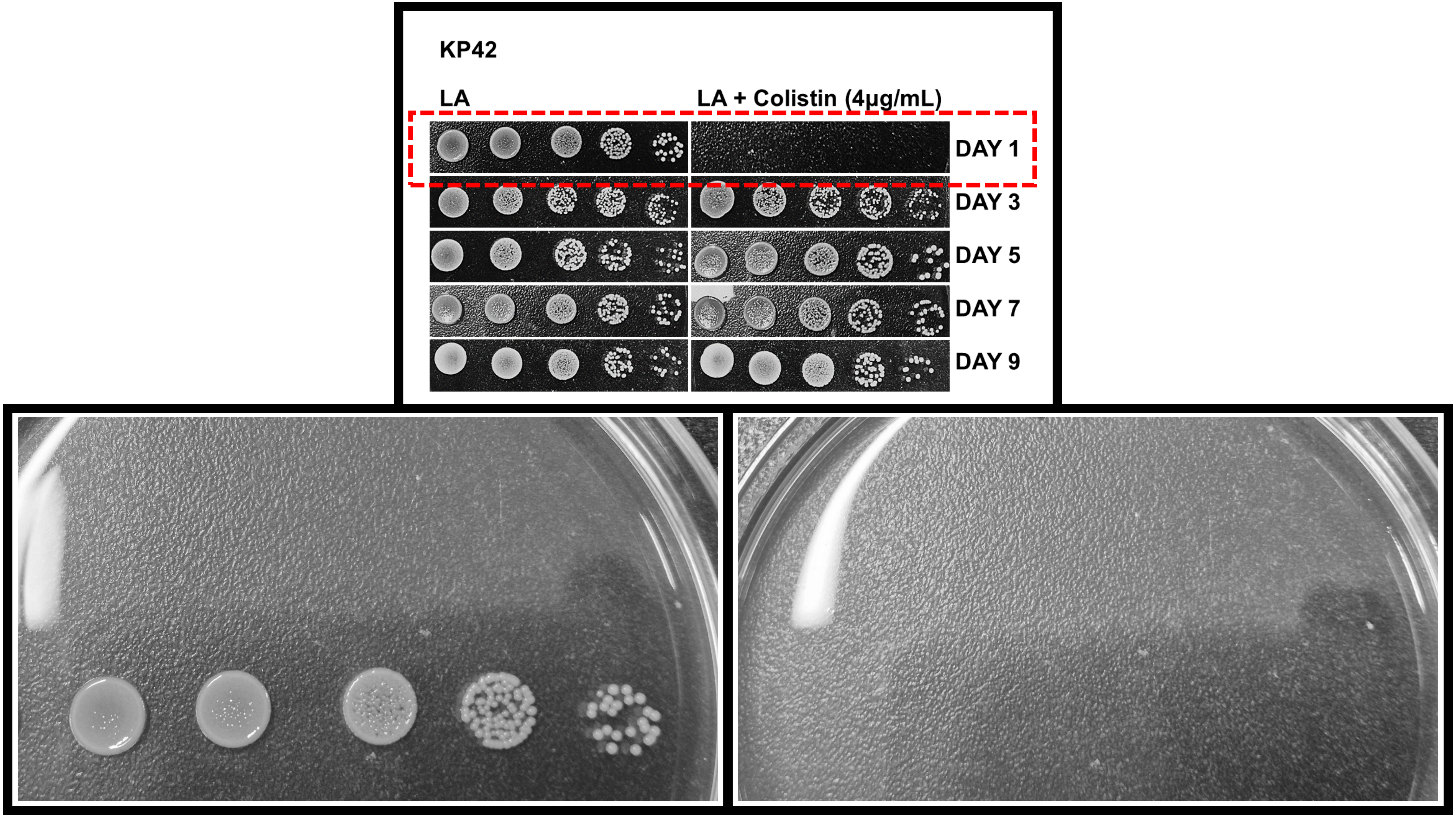
KP42
LA
LA + Colistin (4μg/mL)
DAY 1
DAY 3
DAY 5
DAY 7
DAY 9

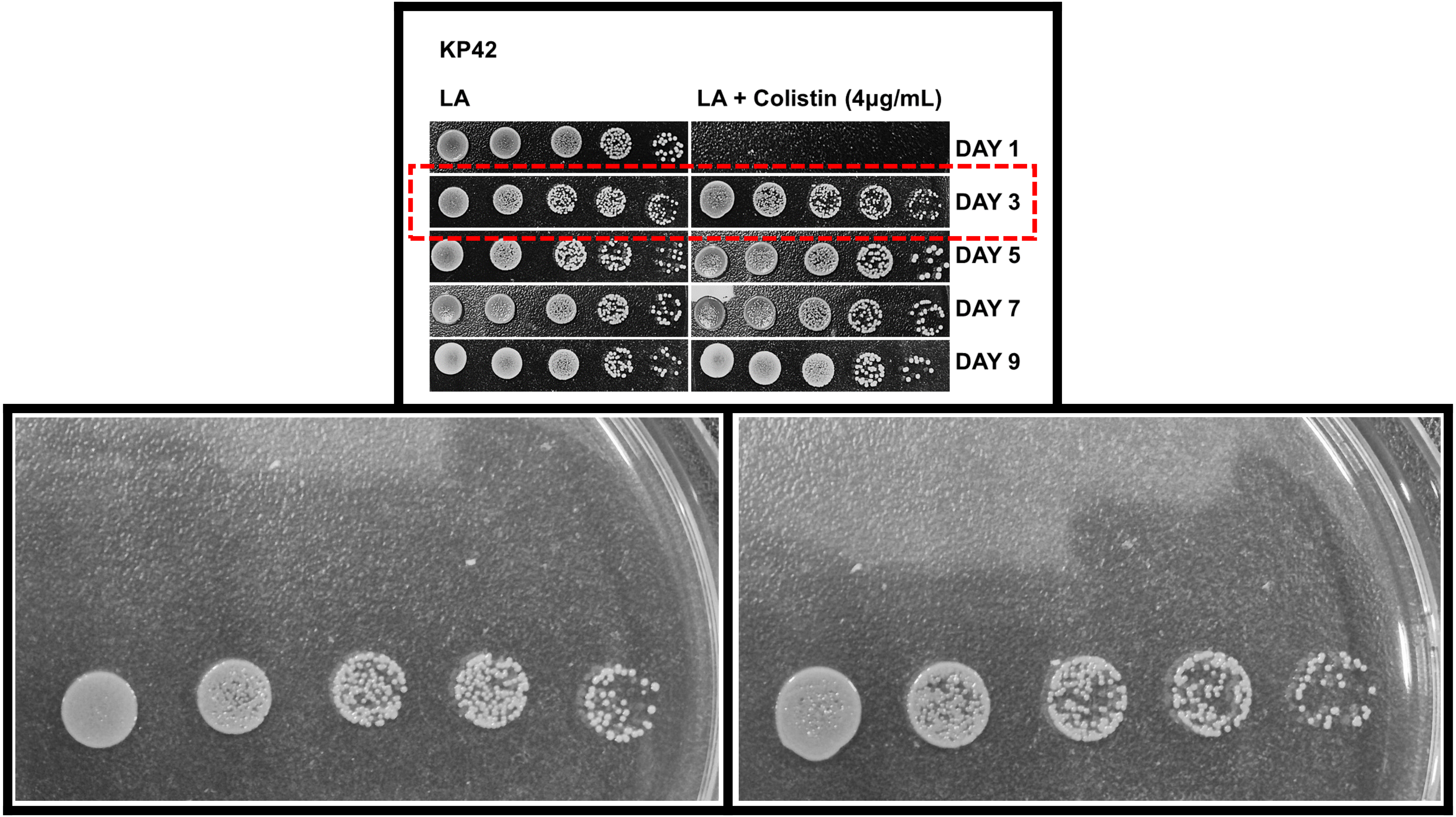

KP42
LA
LA + Colistin (4μg/mL)
DAY 1
DAY 3
DAY 5
DAY 7
DAY 9

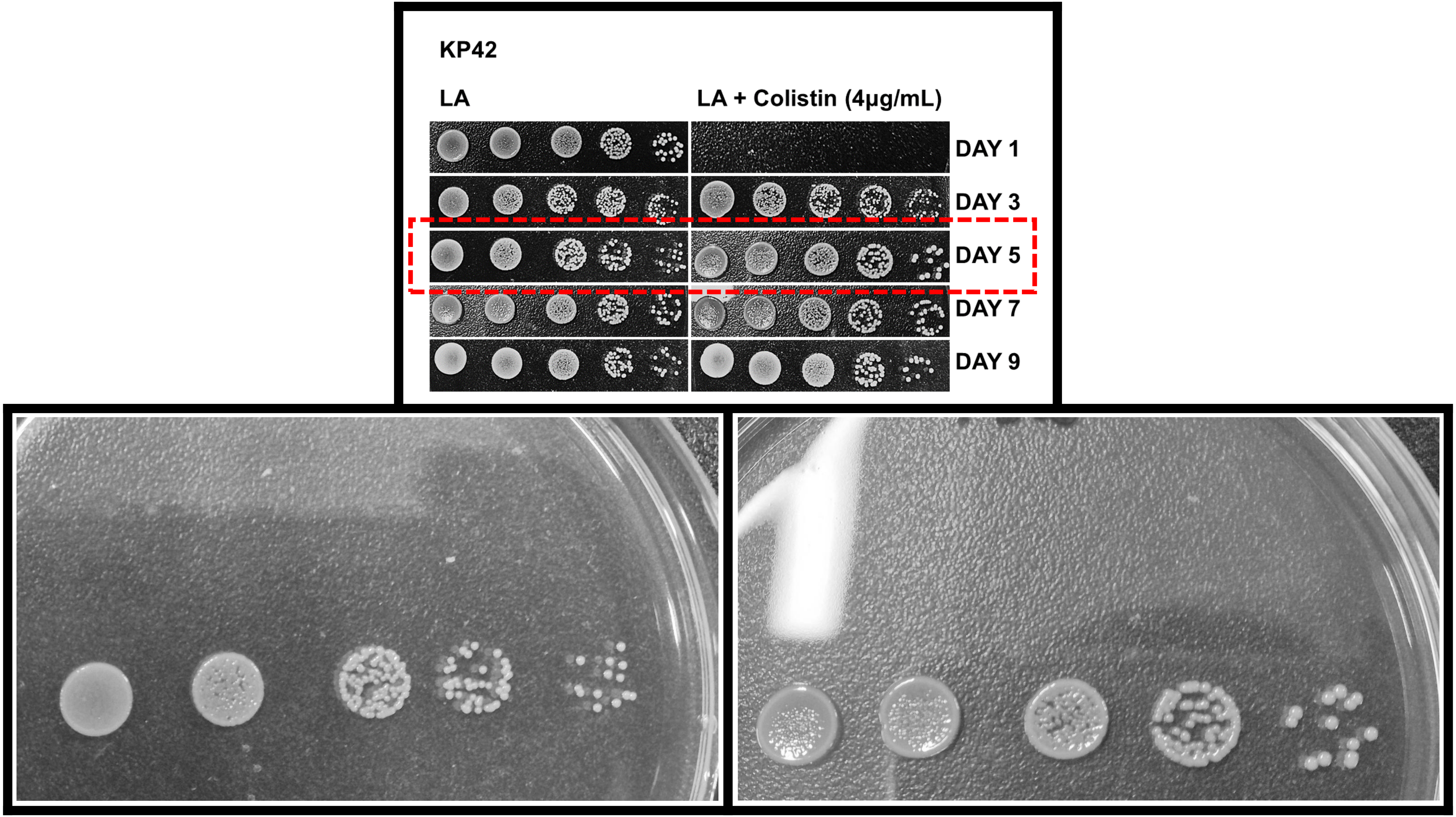

KP42
LA
LA + Colistin (4μg/mL)
DAY 1
DAY 3
DAY 5
DAY 7
DAY 9

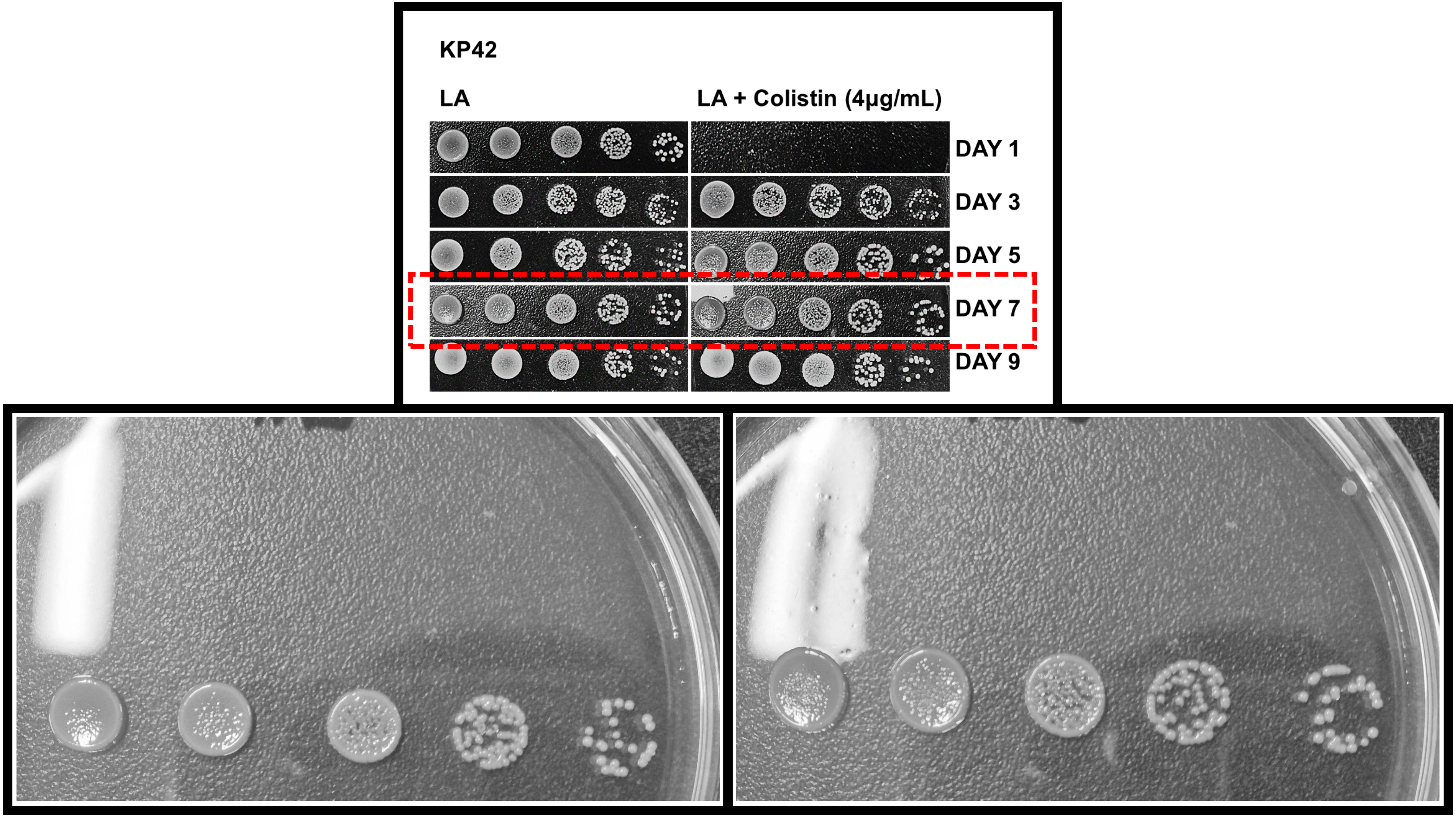

KP42
LA
LA + Colistin (4µg/mL)
DAY 1
DAY 3
DAY 5
DAY 7
DAY 9

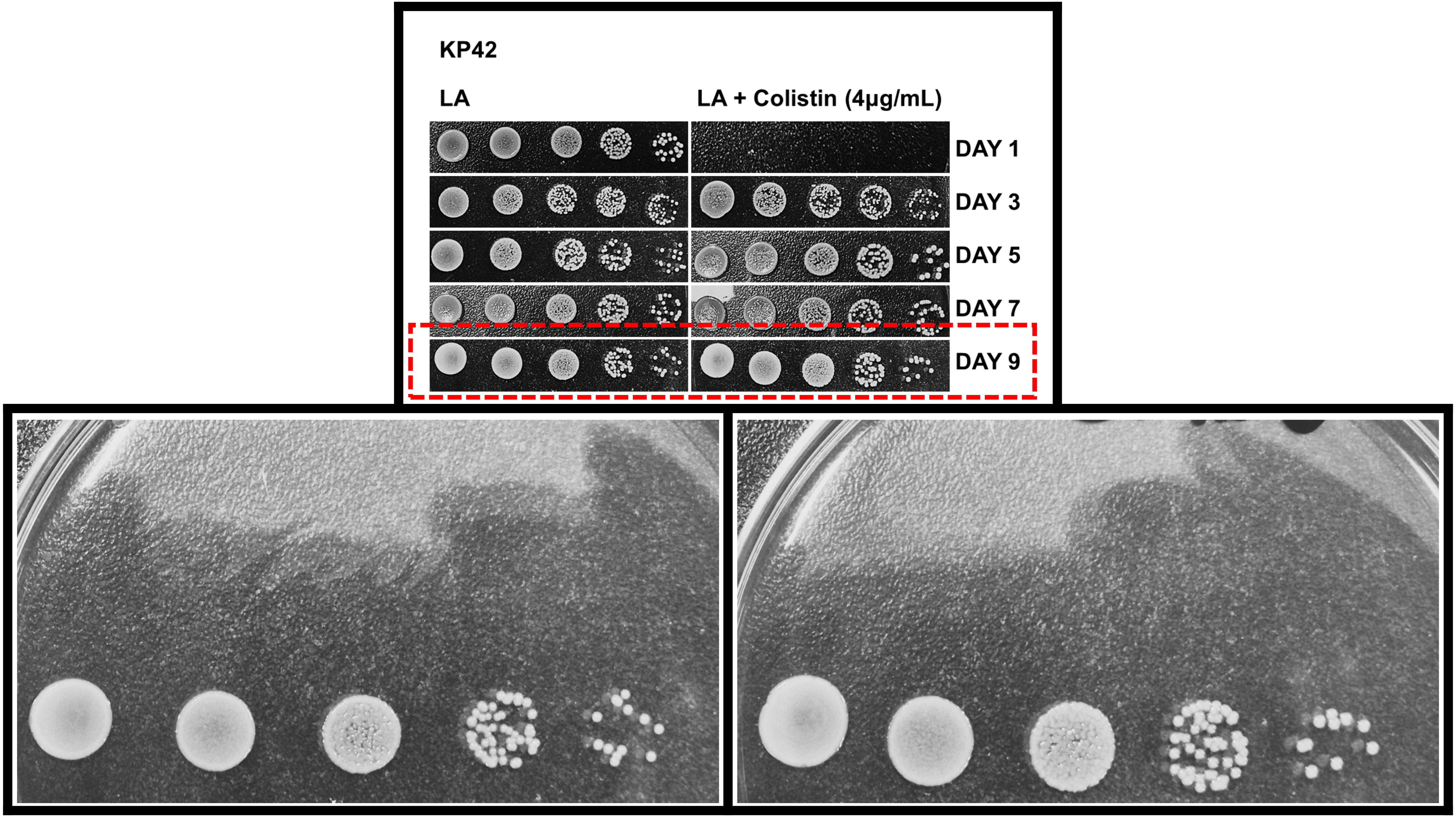
KP42
LA
LA + Colistin (4μg/mL)
DAY 1
DAY 3
DAY 5
DAY 7
DAY 9

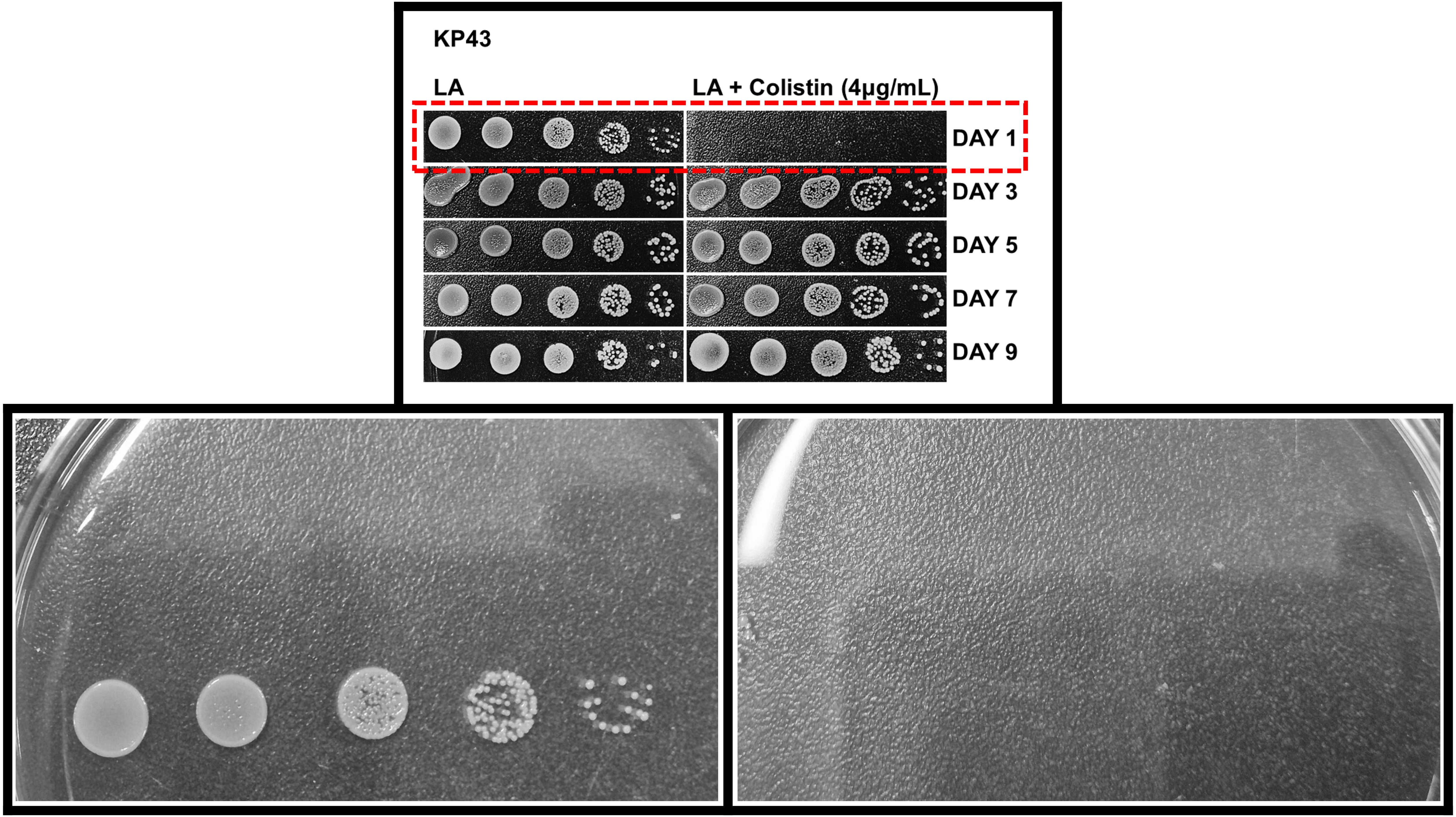
KP43
LA
LA + Colistin (4μg/mL)
DAY 1
DAY 3
DAY 5
DAY 7
DAY 9

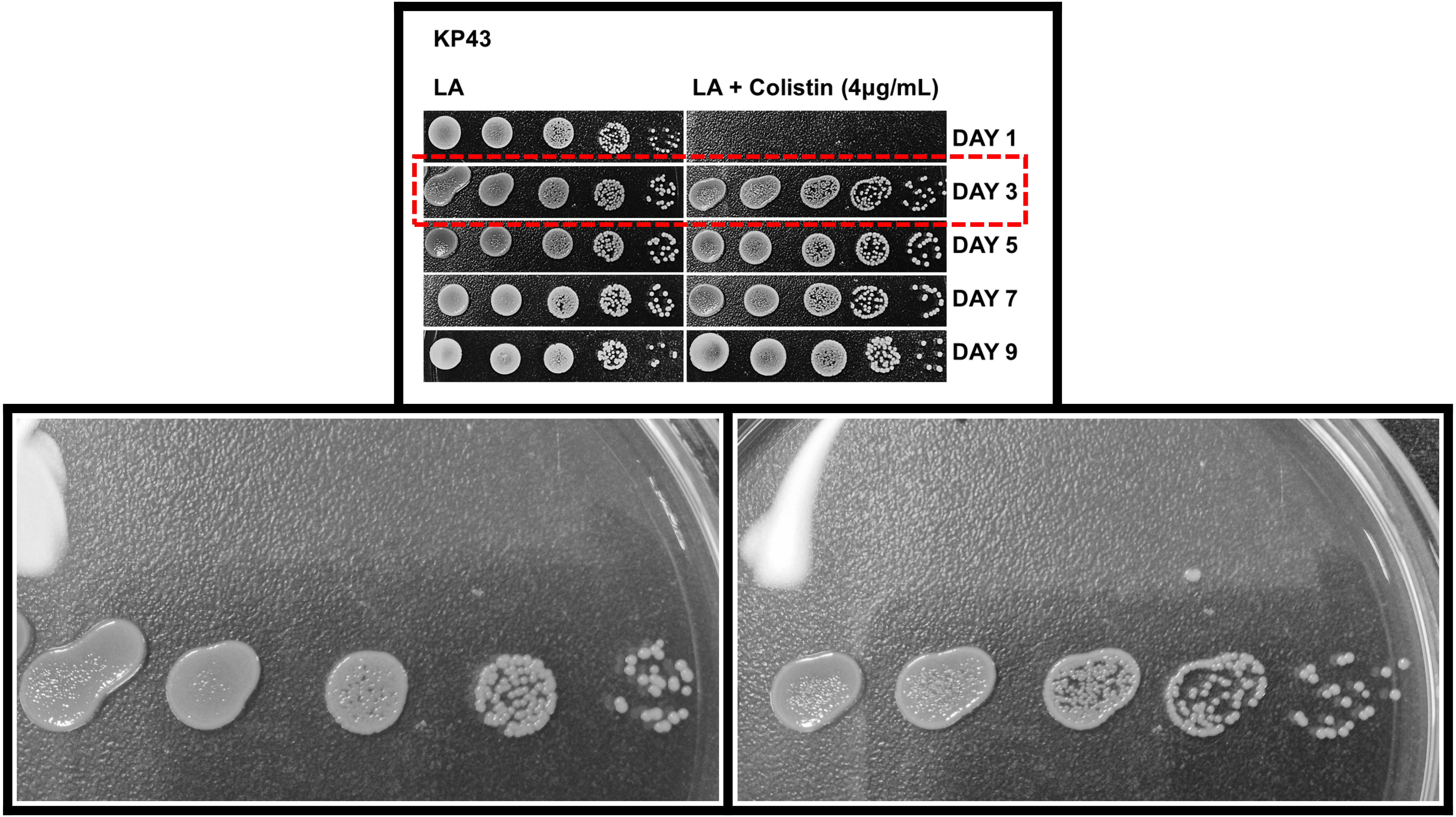
KP43
LA
LA + Colistin (4μg/mL)
DAY 1
DAY 3
DAY 5
DAY 7
DAY 9

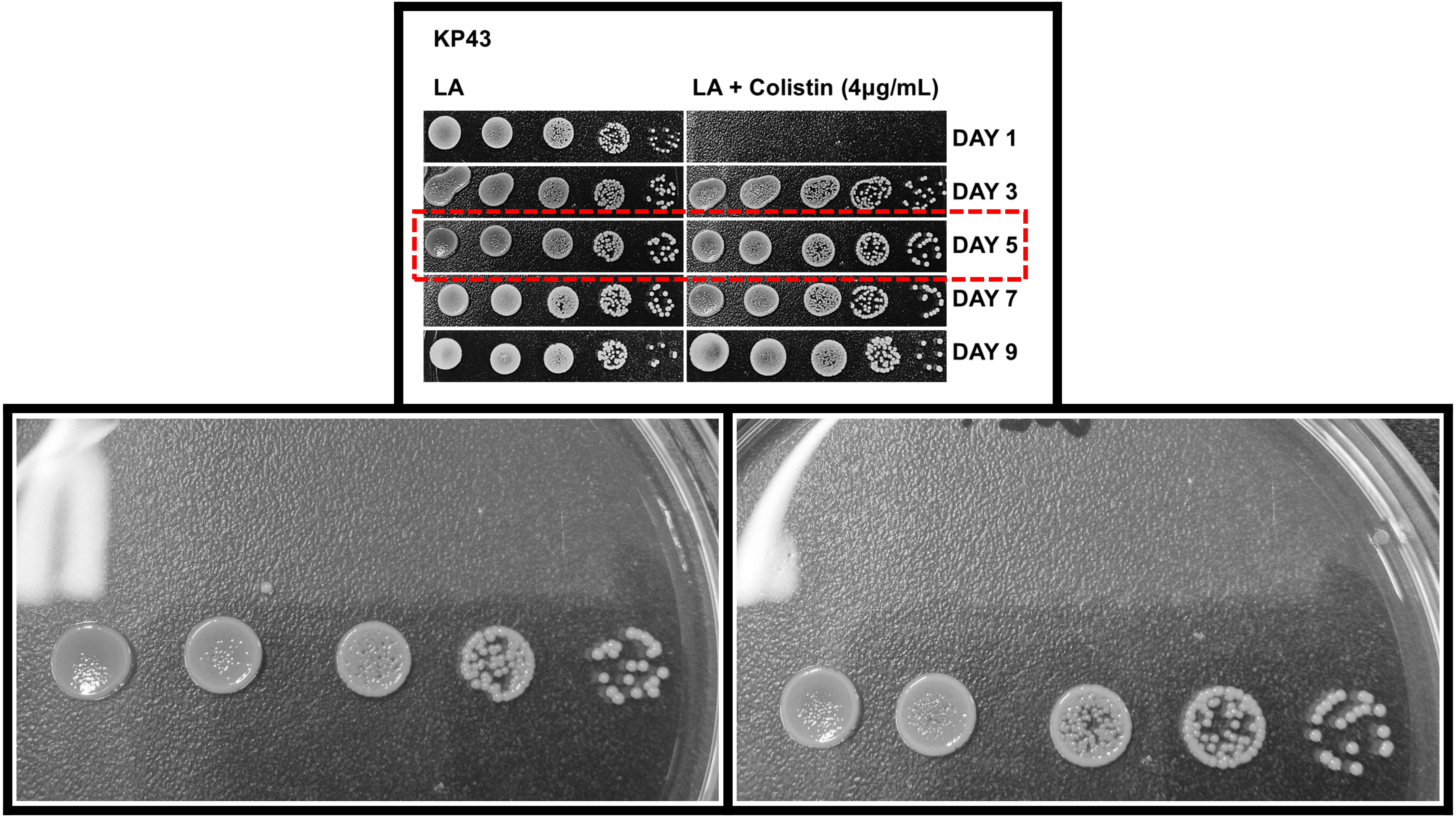
KP43
LA
LA + Colistin (4μg/mL)
DAY 1
DAY 3
DAY 5
DAY 7
DAY 9

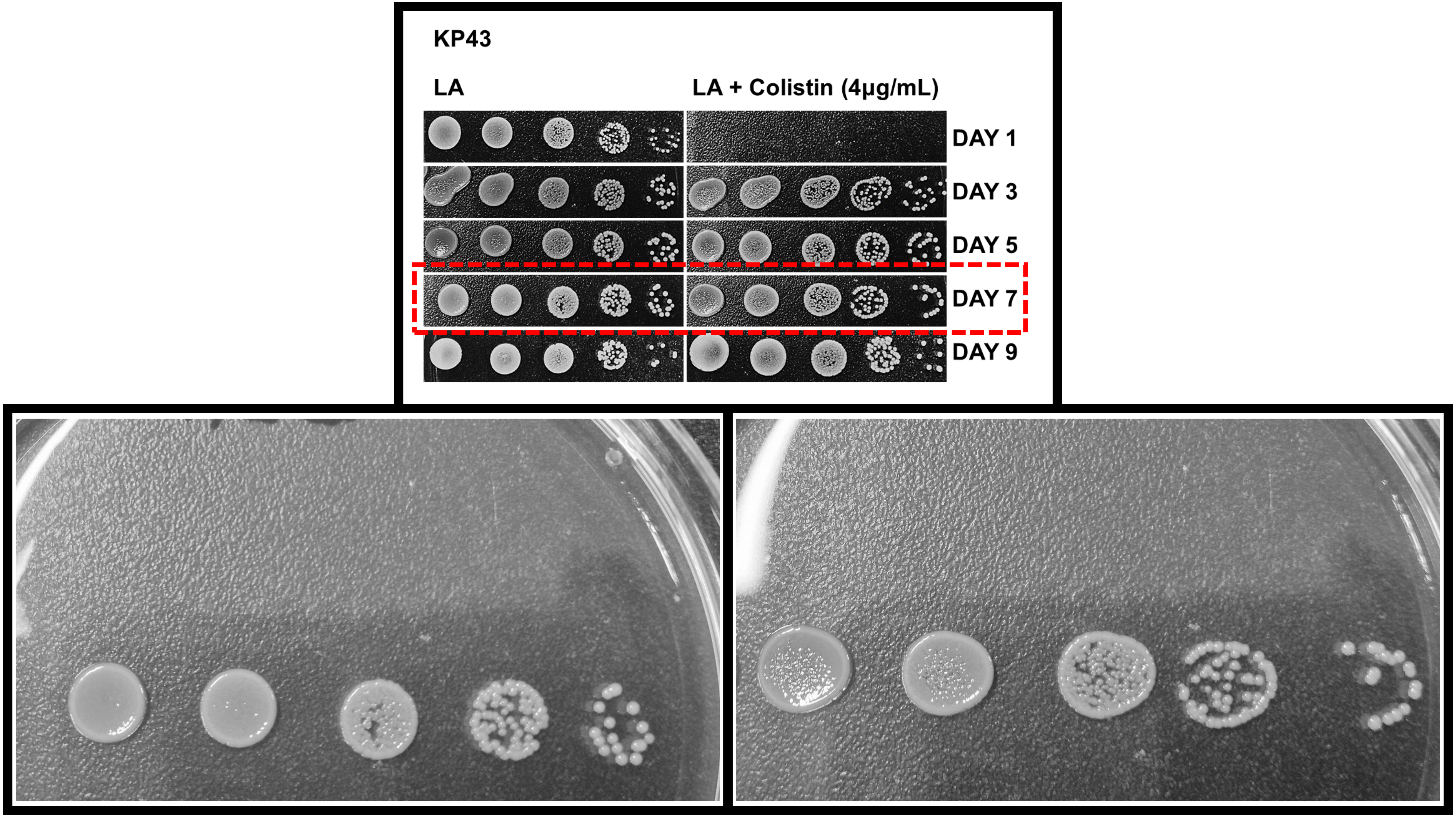
KP43
LA
LA + Colistin (4μg/mL)
DAY 1
DAY 3
DAY 5
DAY 7
DAY 9

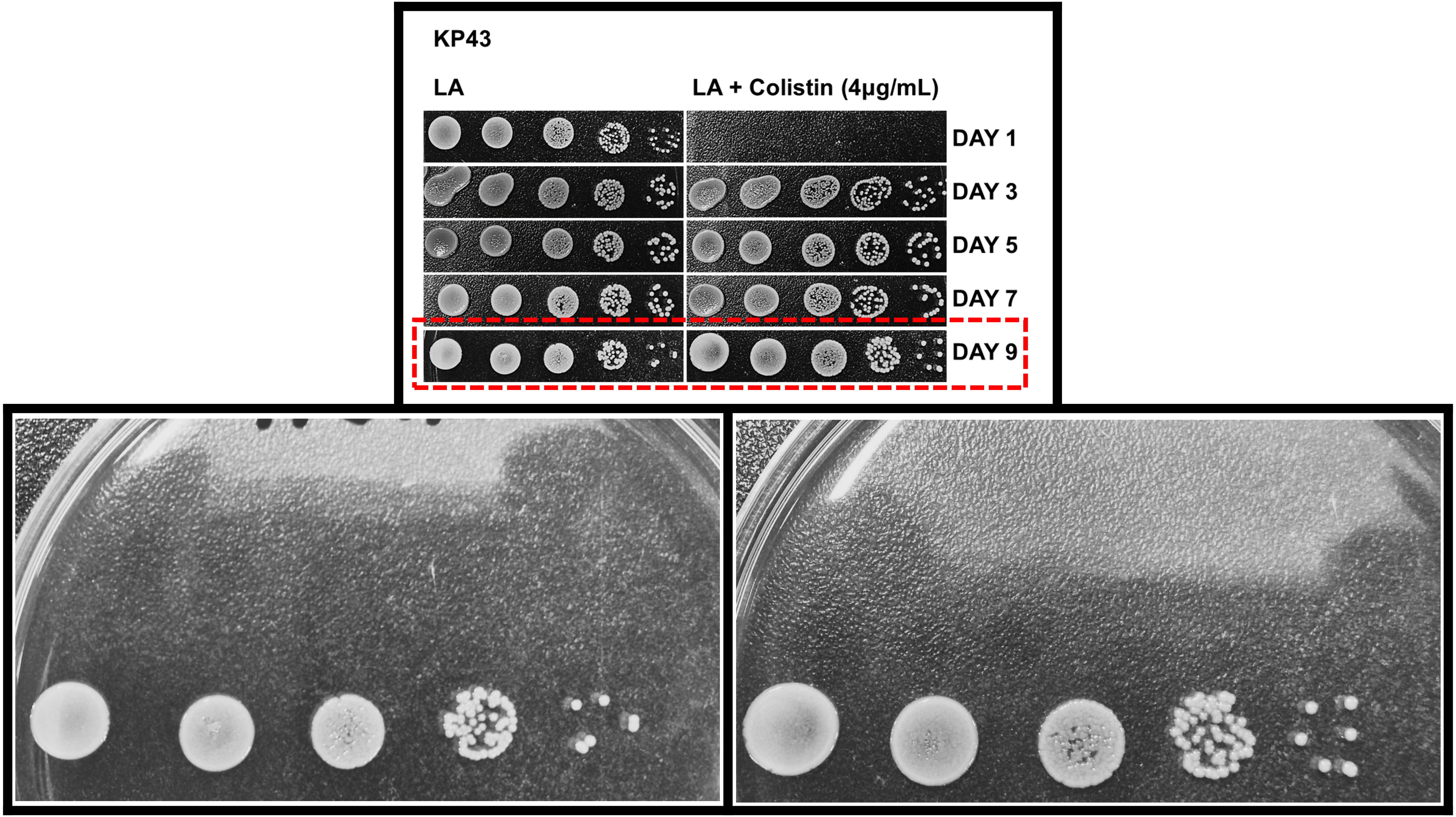
KP43
LA
LA + Colistin (4μg/mL)
DAY 1
DAY 3
DAY 5
DAY 7
DAY 9
