# Supplementary material for: Delineating molecular mechanisms on the acquisition of in vitro-adapted colistin-resistant Klebsiella pneumoniae by transcriptomic analysis
Source: Microbiol Spectr. 2025 Oct 21;13(12):e03428-24. doi: 10.1128/spectrum.03428-24 (PMC12671167; doi:10.1128/spectrum.03428-24)
Supplement: Supplemental material — Supplemental figures and tables. [file spectrum.03428-24-s0002.pdf]

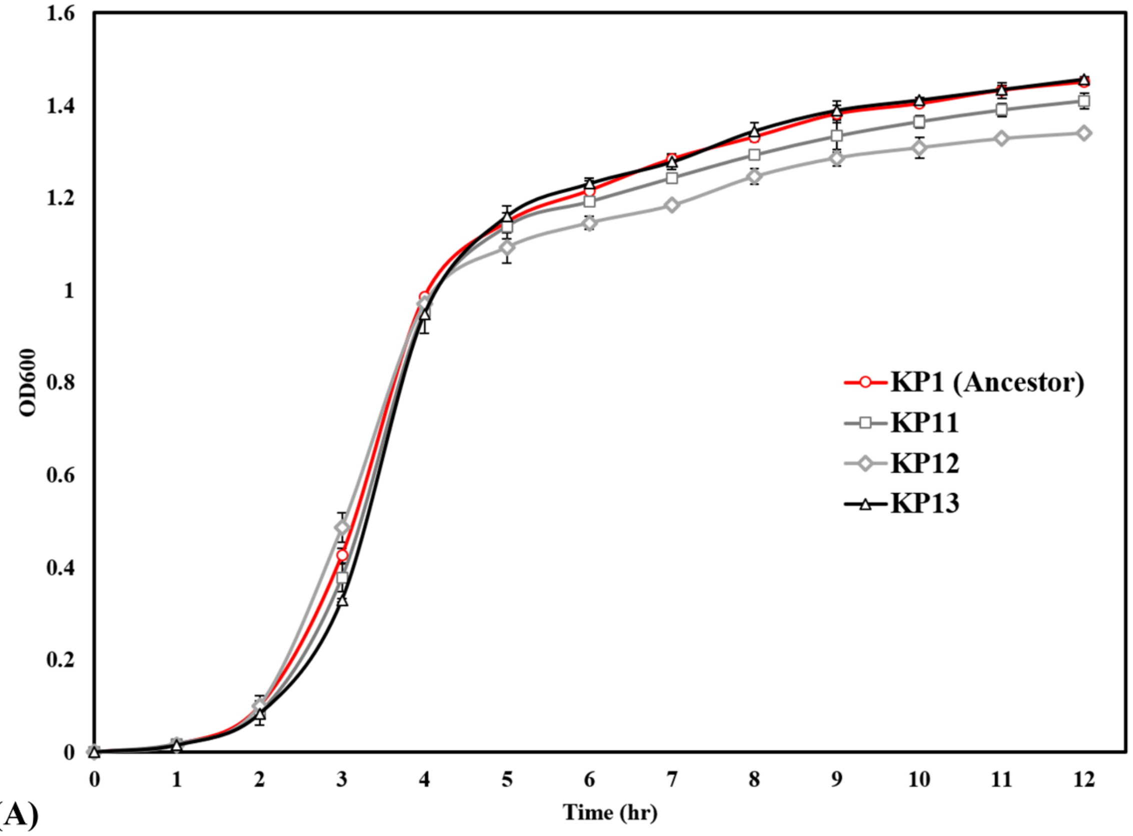

OD600
Time (hr)
KP1 (Ancestor)
KP11
KP12
KP13

(A)

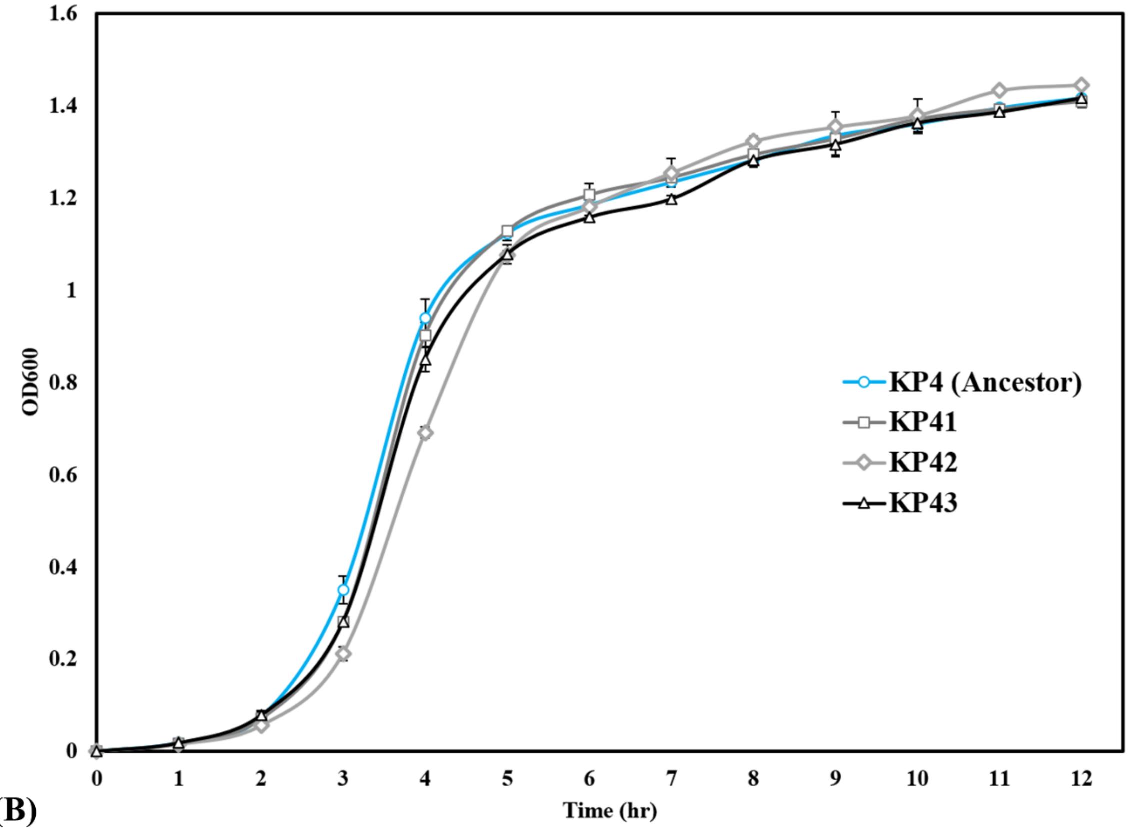

OD600
Time (hr)
KP4 (Ancestor)
KP41
KP42
KP43

(B)

Figure S1. Growth rate comparison of subjected strains. Growth rates of adapted strains were compared through growth curve analysis. Three biological replicates were performed for each strain by inoculating cultures into fresh Luria-Bertani (LB) broth, with OD_{600} measured hourly over 12 hours. In (A), KP1-adapted strains were compared to their ancestor, while KP4 descendants were compared to their ancestor in (B).

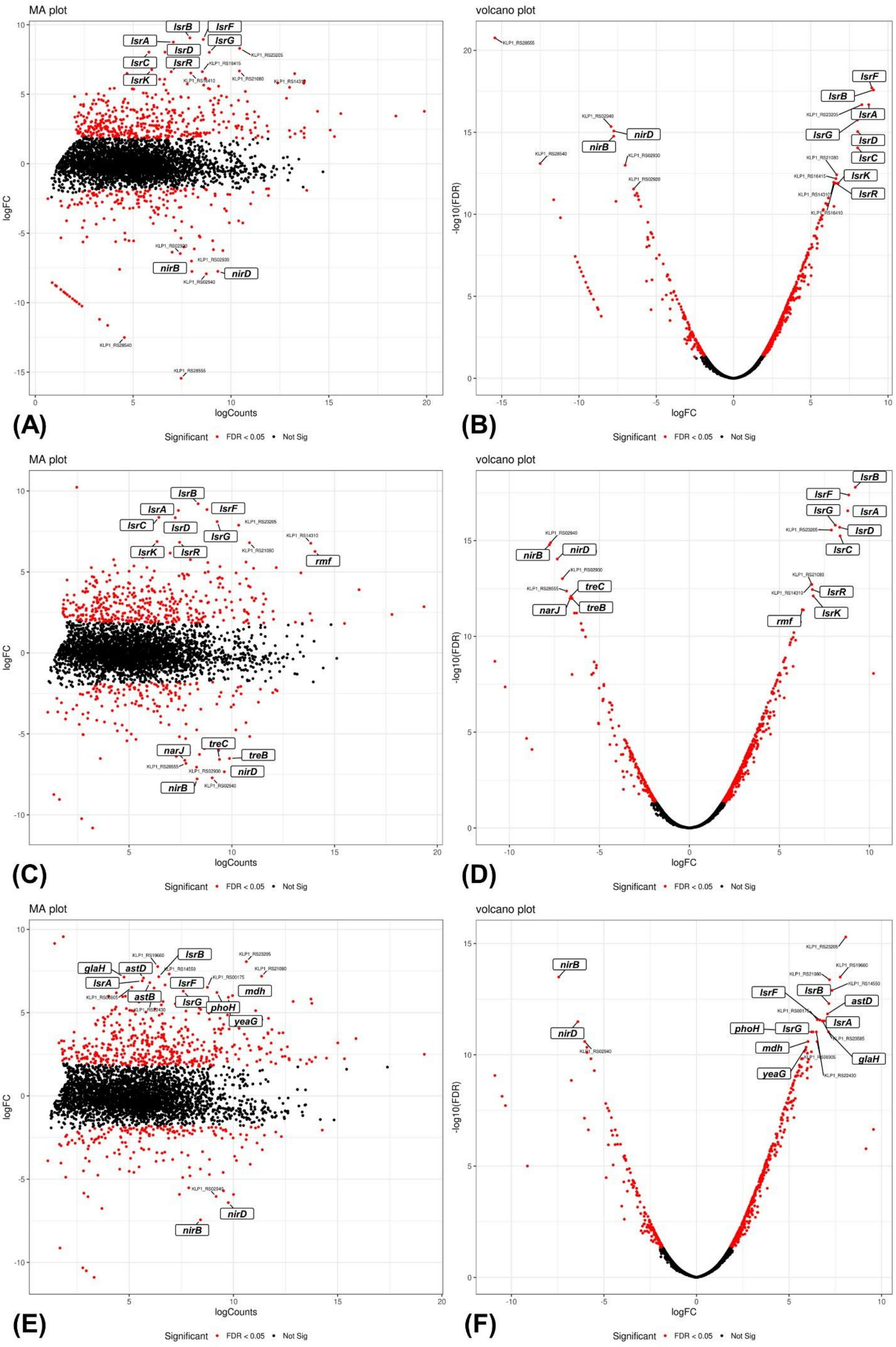
MA plot
volcano plot
logFC
logCounts
-log10(FDR)
lsrB
lsrF
lsrA
lsrG
lsrD
lsrC
lsrR
lsrK
KLP1_RS23205
KLP1_RS16415
KLP1_RS16410
KLP1_RS21080
KLP1_RS14310
KLP1_RS02920
KLP1_RS02930
KLP1_RS02940
KLP1_RS28540
KLP1_RS28555
nirB
nirD
rmf
treC
treB
narJ
glaH
astD
astB
mdh
phoH
yeaG
KLP1_RS19660
KLP1_RS14550
KLP1_RS00175
KLP1_RS06905
KLP1_RS22430
KLP1_RS23585
(A)
(B)
(C)
(D)
(E)
(F)
Significant
FDR < 0.05
Not Sig

Figure S2. The MA plots and volcano plots presenting significantly differentially expressed genes during day 1 of KP1 adaptation. Genes with a false discovery rate (FDR) < 0.05 were deemed significantly differentially expressed genes (FDR <0.05) and were subjected to statistical analysis using MA plots and volcano plots. The MA plots display the log-fold change (M-value) versus the mean expression intensity (A-value) for each gene, while the volcano plots illustrate the negative logarithm of the p-value versus the log-fold change (or effect size) for each feature. Transcriptomic plots representing KP11, KP12, and KP13 are shown in Figures A-B, C-D, and E-F, respectively. During the early adaptation phase, genes involved in the AI-2 degradation pathway, including *lsrABCD*, *lsrFG*, and *lsrK*, were significantly upregulated. In contrast, the nitrite reductase subunit genes *nirD* and *nirB* were consistently downregulated across all KP1-derived strains.

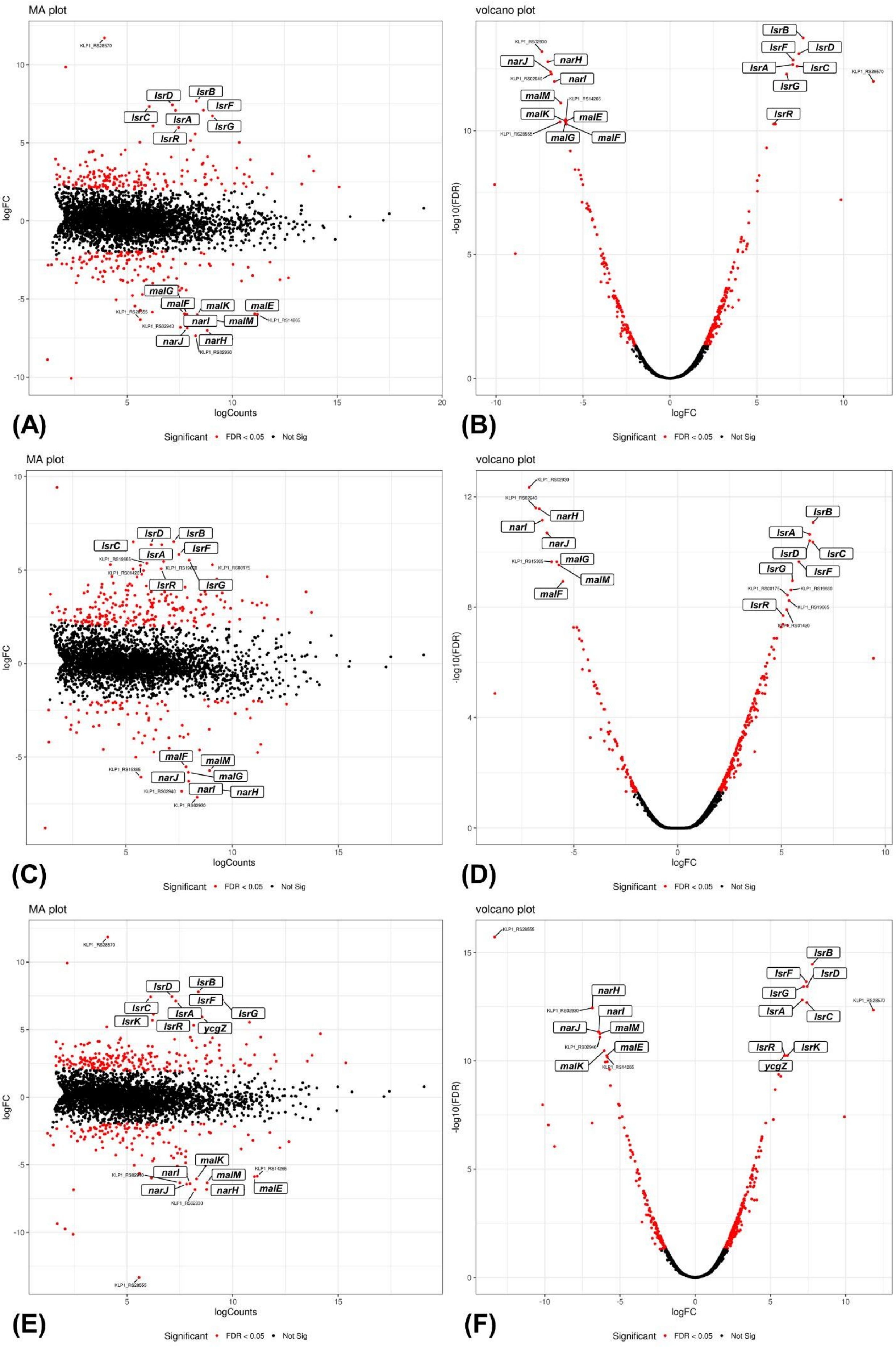
MA plot
volcano plot
logFC
logCounts
-log10(FDR)
Significant
FDR < 0.05
Not Sig
lsrB
lsrD
lsrF
lsrC
lsrA
lsrG
lsrR
lsrK
ycgZ
malG
malF
malK
malE
malM
narI
narJ
narH
KLP1_RS28570
KLP1_RS28555
KLP1_RS02940
KLP1_RS02930
KLP1_RS14265
KLP1_RS15365
KLP1_RS19665
KLP1_RS19660
KLP1_RS01420
KLP1_RS00175
(A)
(B)
(C)
(D)
(E)
(F)

Figure S3. The MA plots and volcano plots presenting significantly differentially expressed genes during day 1 of KP4 adaptation. Genes with a false discovery rate (FDR) < 0.05 were deemed significantly differentially expressed genes (FDR <0.05) and were subjected to statistical analysis using MA plots and volcano plots. The MA plots display the log-fold change (M-value) versus the mean expression intensity (A-value) for each gene, while the volcano plots illustrate the negative logarithm of the p-value versus the log-fold change (or effect size) for each feature. The characteristically differentiated genes were labeled in each figure. Transcriptomic plots representing KP41, KP42, and KP43 are shown in Figures A-B, C-D, and E-F, respectively. During the early adaptation phase, genes involved in the AI-2 degradation pathway, including *lsrABCD*, *lsrFG*, and *lsrK*, were significantly upregulated. In contrast, the nitrite reductase subunit genes *nirD* and *nirB* were consistently downregulated across all KP1-derived strains.

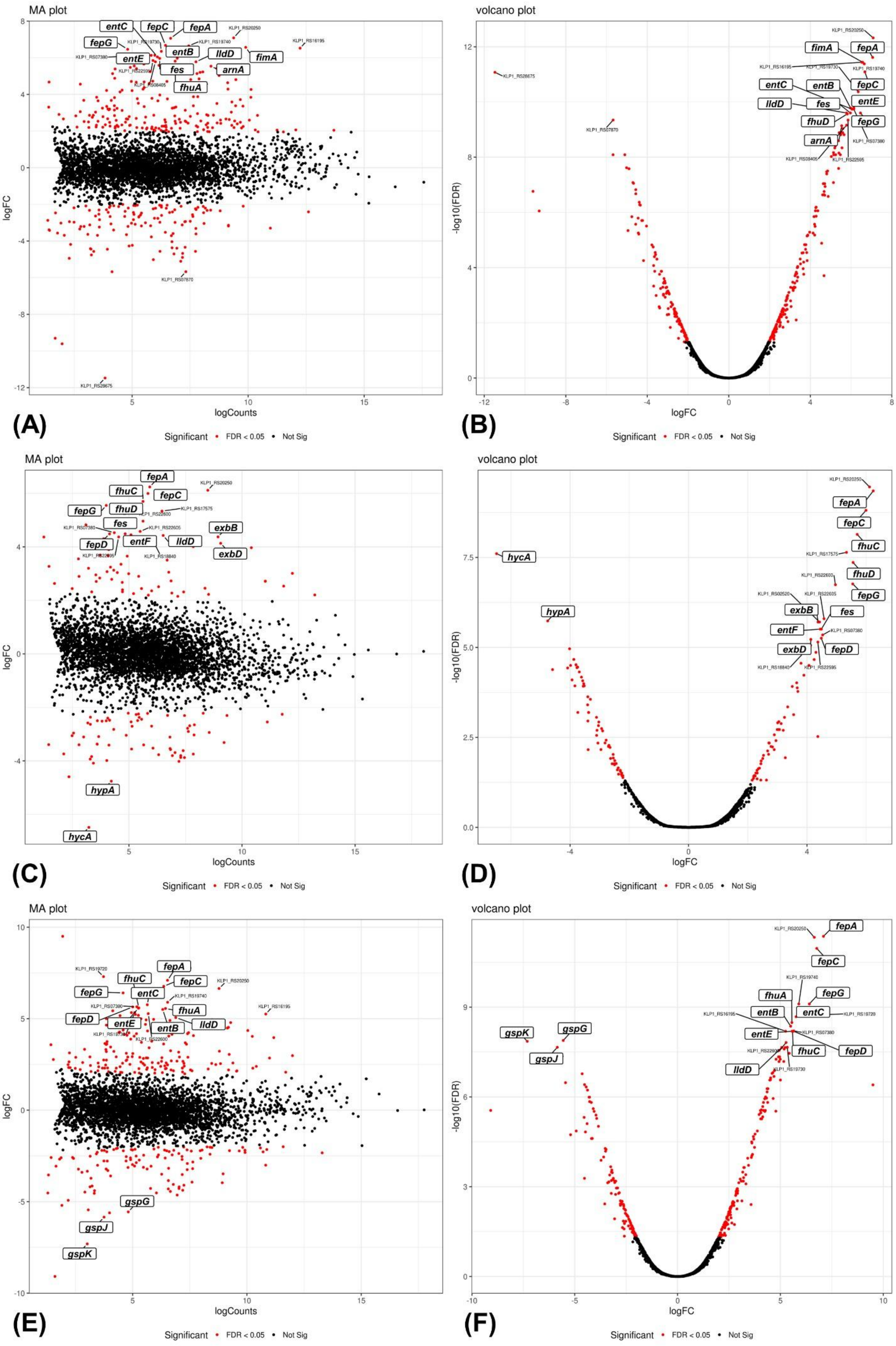

MA plot
logFC
logCounts
entC
fepC
fepA
fepG
entE
entB
lldD
fimA
fes
arnA
fhuA
KLP1_RS20250
KLP1_RS16195
KLP1_RS19740
KLP1_RS19730
KLP1_RS07380
KLP1_RS22595
KLP1_RS08405
KLP1_RS07870
KLP1_RS26675
(A)
Significant
FDR < 0.05
Not Sig
volcano plot
-log10(FDR)
logFC
KLP1_RS26675
KLP1_RS07870
fimA
fepA
fepC
entC
entB
fes
lldD
fhuD
entE
fepG
arnA
(B)
MA plot
fepA
fhuC
fepC
fepG
fhuD
fes
fepD
entF
lldD
exbB
exbD
hypA
hycA
KLP1_RS20250
KLP1_RS17575
KLP1_RS07380
KLP1_RS22605
KLP1_RS18840
(C)
volcano plot
hycA
hypA
fepA
fepC
fhuC
fhuD
fepG
exbB
fes
entF
exbD
fepD
KLP1_RS20250
KLP1_RS17575
KLP1_RS22600
KLP1_RS02520
KLP1_RS22605
KLP1_RS07380
KLP1_RS18840
KLP1_RS22595
(D)
MA plot
KLP1_RS19720
fhuC
fepA
fepC
fepG
entC
fepD
entE
entB
fhuA
lldD
gspG
gspJ
gspK
KLP1_RS20250
KLP1_RS19740
KLP1_RS07380
KLP1_RS16195
KLP1_RS22600
(E)
volcano plot
fepA
fepC
fhuA
fepG
entB
entC
entE
fhuC
fepD
lldD
gspK
gspG
gspJ
KLP1_RS20250
KLP1_RS19740
KLP1_RS16195
KLP1_RS19720
KLP1_RS07380
KLP1_RS19730
(F)

Figure S4. The MA plots and volcano plots presenting significantly differentially expressed genes during later days of KP1 adaptation. Genes with a false discovery rate (FDR) < 0.05 were deemed significantly differentially expressed genes (FDR <0.05) and were subjected to statistical analysis using MA plots and volcano plots. The MA plots display the log-fold change (M-value) versus the mean expression intensity (A-value) for each gene, while the volcano plots illustrate the negative logarithm of the p-value versus the log-fold change (or effect size) for each feature. Transcriptomic plots representing KP11, KP12, and KP13 are shown in Figures A-B, C-D, and E-F, respectively. In the later stages of adaptation, genes involved with the enterobactin biosynthesis pathway, the polymyxin resistance pathway, ferric enterobactin outer membrane transport complex gene *fepA*, and ferric enterochelin esterase harboring *fes* were confirmed to be upregulated in all strains.

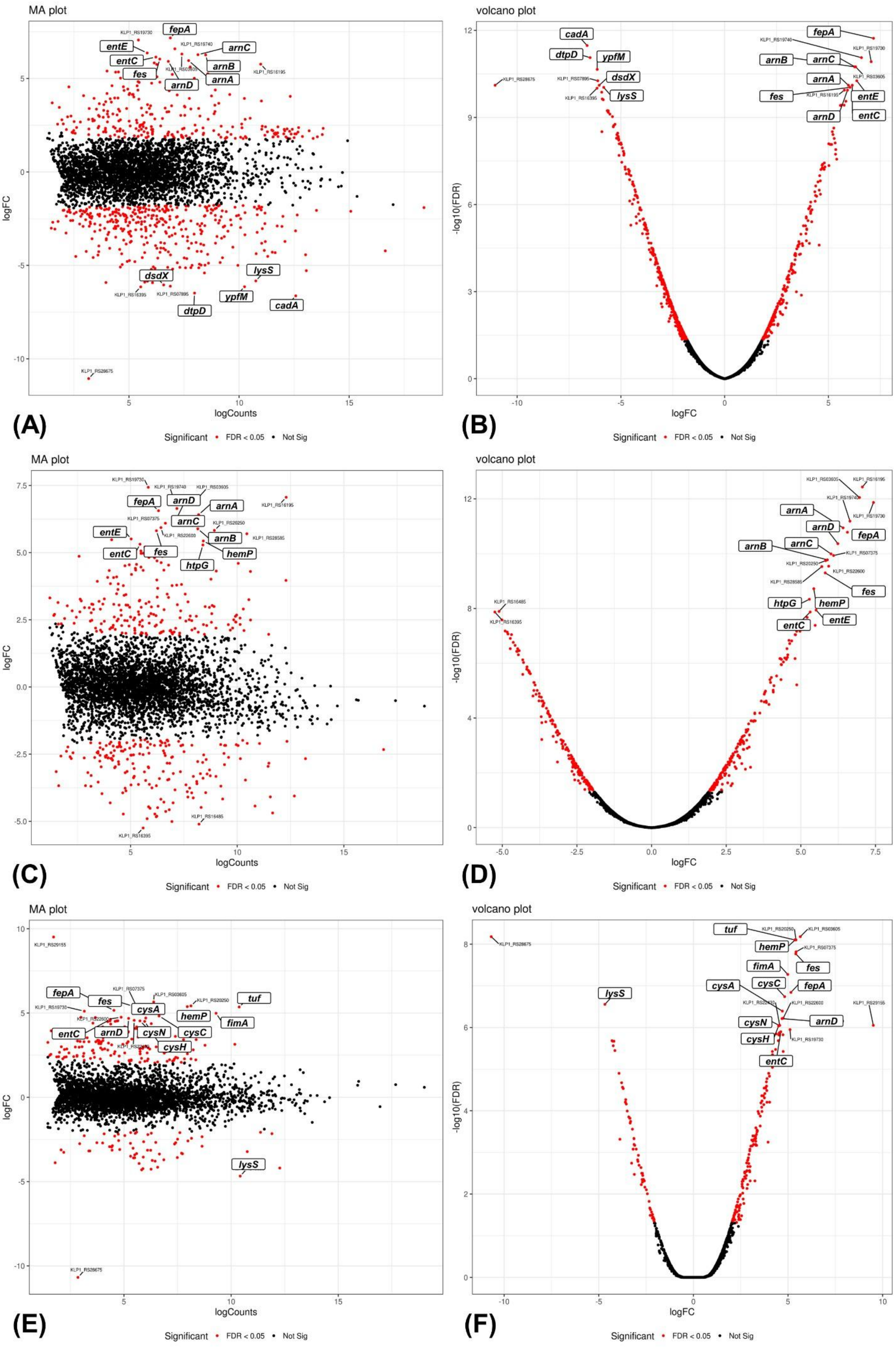

MA plot
fepA
entE
arnC
entC
fes
arnB
arnA
arnD
dsdX
lysS
ypfM
cadA
dtpD
logFC
logCounts
(A)
Significant • FDR < 0.05 • Not Sig
volcano plot
cadA
dtpD
ypfM
dsdX
lysS
fepA
arnB
arnC
arnA
fes
entE
arnD
entC
-log10(FDR)
logFC
(B)
Significant • FDR < 0.05 • Not Sig
MA plot
fepA
arnD
arnA
arnC
entE
arnB
entC
fes
hemP
htpG
logFC
logCounts
(C)
Significant • FDR < 0.05 • Not Sig
volcano plot
arnA
arnD
fepA
arnB
arnC
fes
htpG
hemP
entC
entE
-log10(FDR)
logFC
(D)
Significant • FDR < 0.05 • Not Sig
MA plot
fepA
fes
cysA
tuf
hemP
fimA
entC
arnD
cysN
cysC
cysH
lysS
logFC
logCounts
(E)
Significant • FDR < 0.05 • Not Sig
volcano plot
tuf
hemP
fimA
fes
cysA
cysC
fepA
lysS
cysN
arnD
cysH
entC
-log10(FDR)
logFC
(F)
Significant • FDR < 0.05 • Not Sig

Figure S5. The MA plots and volcano plots presenting significantly differentially expressed genes during later days of KP4 adaptation. Genes with a false discovery rate (FDR) < 0.05 were deemed significantly differentially expressed genes (FDR <0.05) and were subjected to statistical analysis using MA plots and volcano plots. The MA plots display the log-fold change (M-value) versus the mean expression intensity (A-value) for each gene, while the volcano plots illustrate the negative logarithm of the p-value versus the log-fold change (or effect size) for each feature. Transcriptomic plots representing KP11, KP12, and KP13 are shown in Figures A-B, C-D, and E-F, respectively. In the later stages of adaptation, genes involved with the enterobactin biosynthesis pathway, the polymyxin resistance pathway, ferric enterobactin outer membrane transport complex gene *fepA*, and ferric enterochelin esterase harboring *fes* were confirmed to be upregulated in all strains.

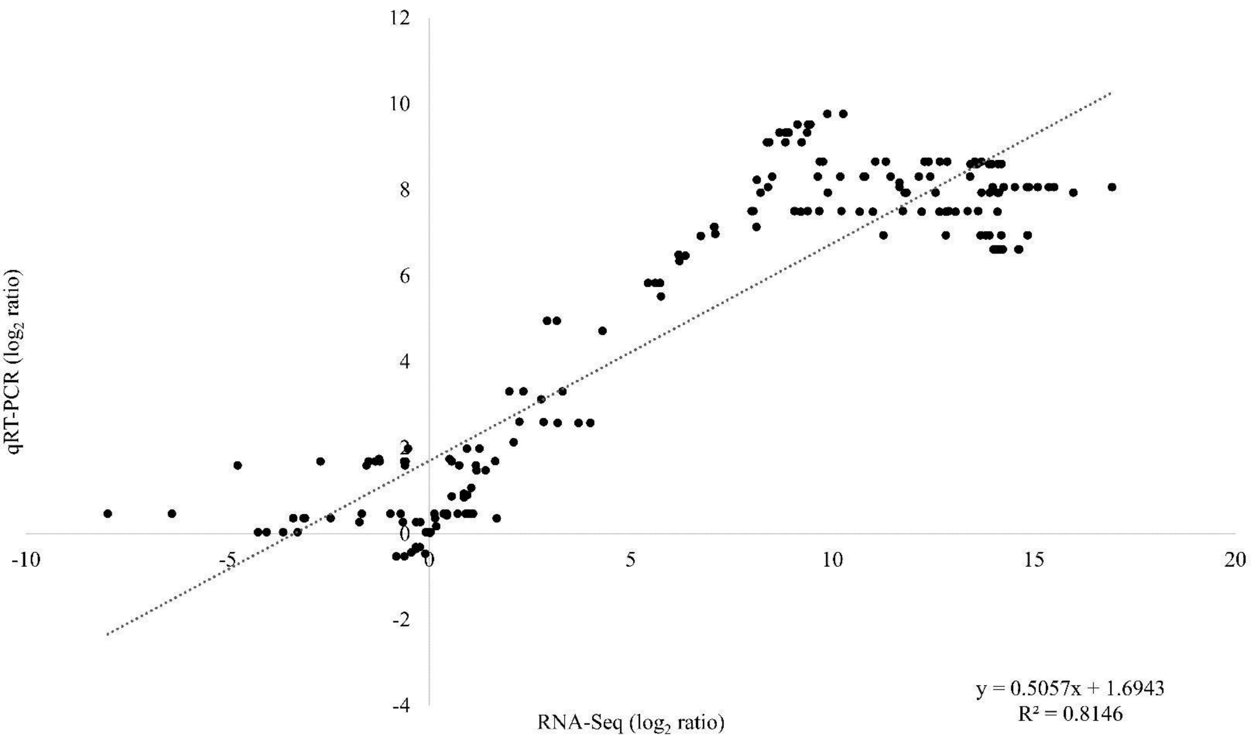

Figure S6. The correlation coefficient of the expression values from the RNA-seq and qRT-PCR data. Pearson correlation coefficient was calculated to confirm the result of the RNA-seq datasets. The correlation coefficient was 0.8146.

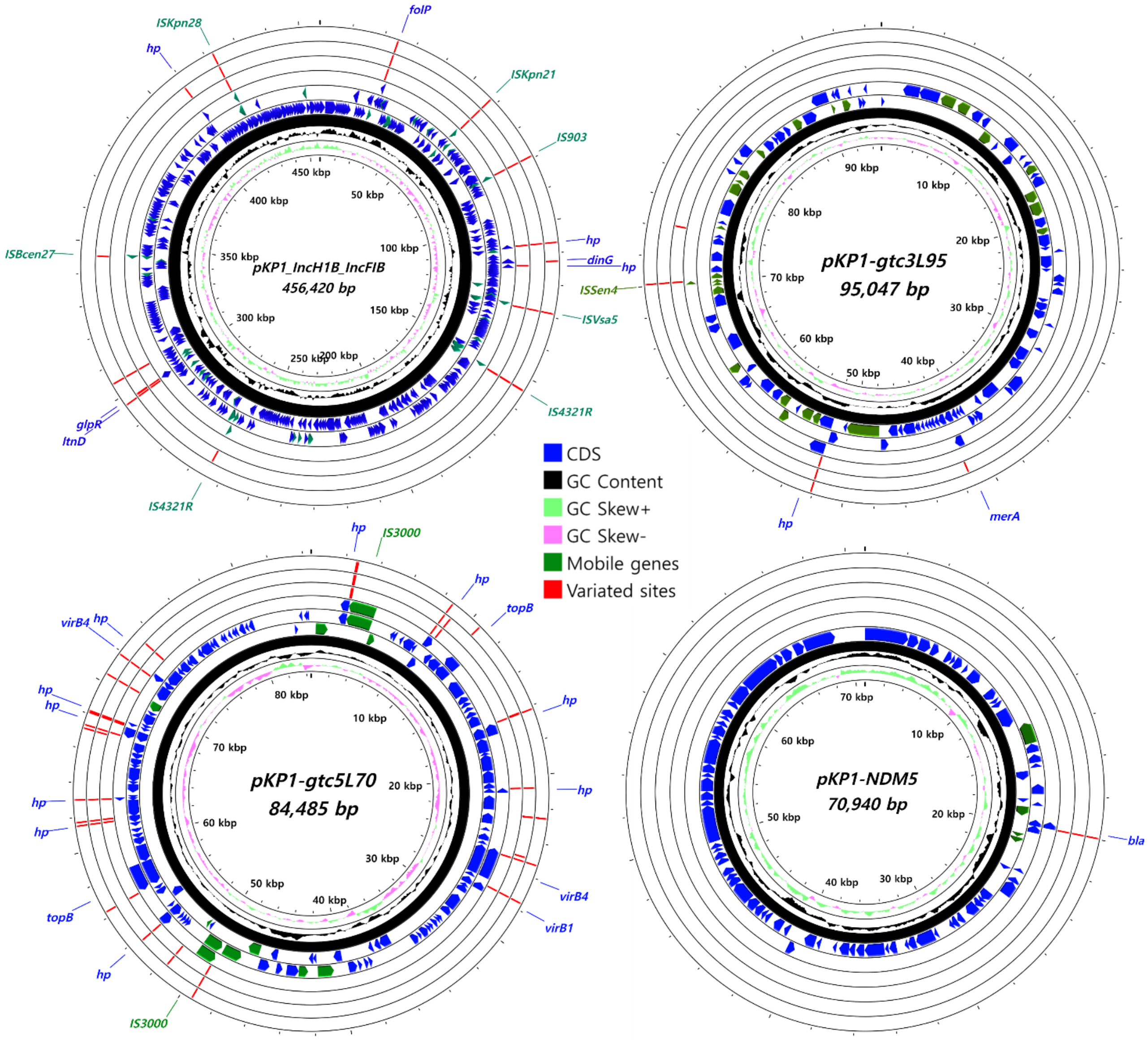

Figure S7. Plasmid map visualization of the locations and characteristics of gene variation sites resulting from ALE of KP1. The genomic locations of each variant site are indicated with red strips in the order of 139, 129, and 119 from the outermost circle inward. The locations of the genes with discovered variants are additionally highlighted on the fourth circle from the outside. The positions of CDS genes are depicted as blue arrows, while mobile genes, such as transposases, are depicted as green arrows. CGView was used as the visualization tool for characterizing the genomic structures.

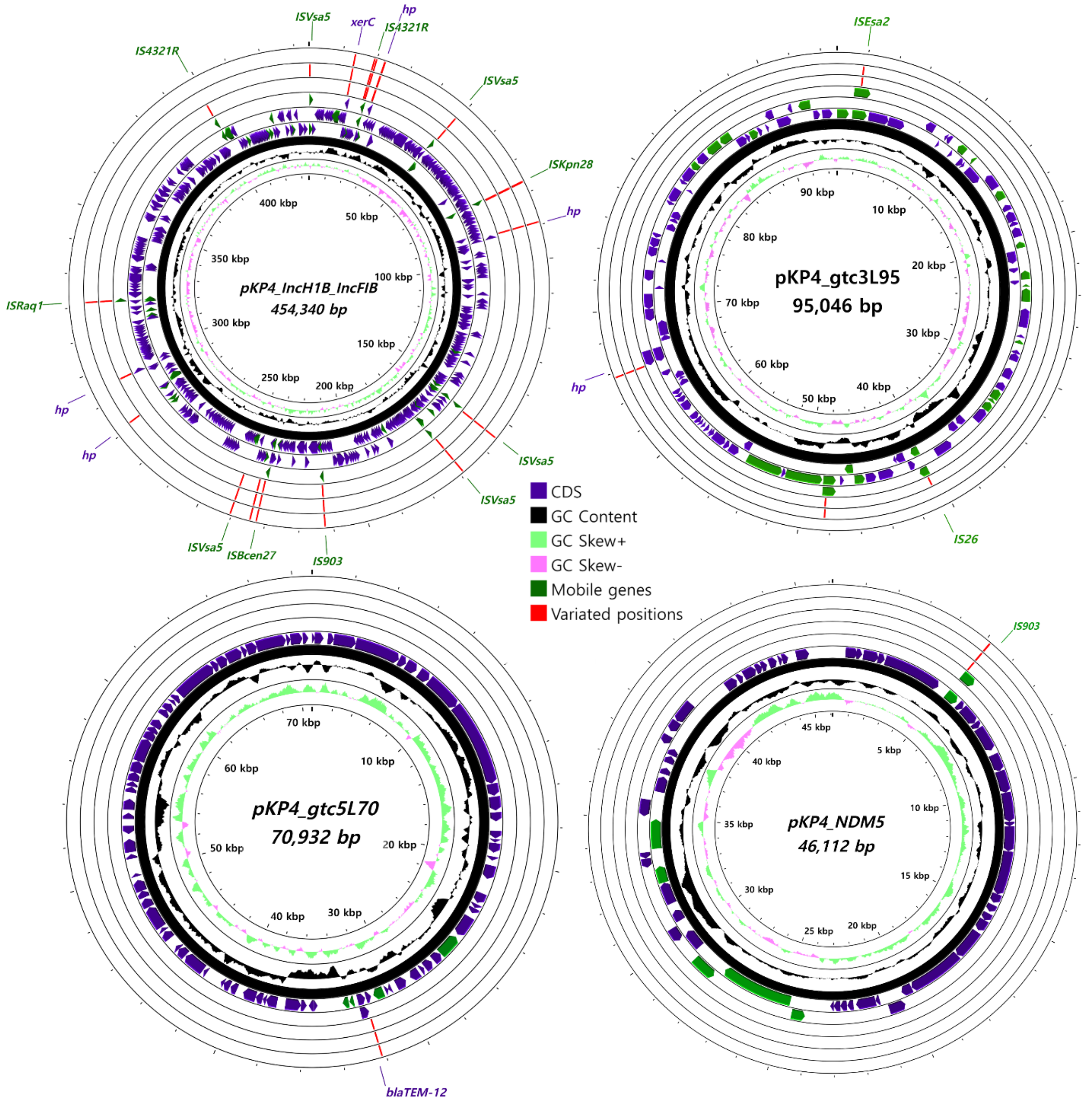

Figure S8. Plasmid map visualization of the locations and characteristics of gene variation sites resulting from ALE of KP4. The genomic locations of each variant site are indicated with red strips in the order of 139, 129, and 119 from the outermost circle inward. The locations of the genes with discovered variants are additionally highlighted on the fourth circle from the outside. The positions of CDS genes are depicted as blue arrows, while mobile genes, such as transposases, are depicted as green arrows. CGView was used as the visualization tool for characterizing the genomic structures.

Table S1. Summary of expression profiling.

Strain	Total reads	Mapped reads	Mapped rate	Count (>0)	Exp (>1)
**KP1**	28,538,759	23,756,518	83.24	4,681	4,128
**KP111**	44,468,195	39,903,713	89.74	4,643	4,215
**KP112**	32,696,333	27,293,335	83.48	4,679	4,182
**KP113**	35,305,523	29,776,071	84.34	4,677	4,203
**KP114**	33,808,436	27,809,475	82.26	4,681	4,168
**KP115**	32,739,344	27,910,685	85.25	4,673	4,152
**KP116**	33,533,288	28,648,089	85.43	4,673	4,133
**KP117**	31,105,405	26,272,192	84.46	4,679	4,215
**KP118**	31,271,000	26,484,489	84.69	4,674	4,150
**KP119**	40,165,194	34,043,596	84.76	4,679	4,196
**KP121**	30,058,443	25,992,330	86.47	4,667	4,258
**KP122**	31,512,650	26,638,588	84.53	4,679	4,194
**KP123**	34,541,919	28,930,001	83.75	4,678	4,232
**KP124**	30,218,688	25,828,996	85.47	4,681	4,210
**KP125**	29,224,245	24,708,446	84.55	4,671	4,188
**KP126**	34,846,297	29,466,114	84.56	4,643	4,146
**KP127**	31,590,317	26,206,192	82.96	4,639	4,146
**KP128**	36,016,453	30,312,906	84.16	4,644	4,187
**KP129**	31,437,900	26,788,703	85.21	4,649	4,220
**KP131**	29,432,196	26,261,720	89.23	4,668	4,154
**KP132**	33,084,031	29,104,634	87.97	4,672	4,199
**KP133**	31,437,581	27,266,054	86.73	4,666	4,173
**KP134**	34,885,278	29,223,314	83.77	4,681	4,130
**KP135**	30,864,018	25,663,566	83.15	4,679	4,136
**KP136**	35,869,622	30,064,325	83.82	4,678	4,077
**KP137**	36,132,826	31,582,045	87.41	4,676	4,086
**KP138**	37,424,727	32,302,884	86.31	4,672	3,998
**KP139**	29,681,862	25,615,979	86.3	4,671	4,168
**KP4**	32,138,712	27,629,904	85.97	4,677	4,155
**KP411**	35,187,735	29,475,449	83.77	4,677	4,236

**KP412**	25,218,091	21,788,016	86.4	4,666	4,232
**KP413**	34,279,645	28,404,892	82.86	4,686	4,144
**KP414**	36,614,845	30,658,719	83.73	4,677	4,083
**KP415**	29,841,639	25,443,133	85.26	4,678	4,119
**KP416**	36,575,100	30,280,099	82.79	4,683	4,136
**KP417**	34,494,298	29,244,516	84.78	4,671	4,063
**KP418**	33,117,146	28,142,905	84.98	4,663	4,078
**KP419**	34,853,969	29,504,149	84.65	4,678	4,060
**KP421**	48,246,174	42,218,151	87.51	4,675	4,140
**KP422**	37,577,435	31,947,686	85.02	4,681	4,132
**KP423**	32,545,396	28,560,594	87.76	4,672	4,179
**KP424**	43,363,580	37,235,700	85.87	4,675	4,105
**KP425**	41,000,727	34,056,477	83.06	4,687	4,101
**KP426**	32,393,201	27,248,539	84.12	4,674	4,077
**KP427**	31,406,718	26,570,896	84.6	4,677	4,119
**KP428**	31,893,533	26,302,451	82.47	4,663	4,039
**KP429**	28,679,467	24,516,390	85.48	4,673	4,101
**KP431**	32,253,916	28,116,326	87.17	4,674	4,205
**KP432**	33,535,745	28,781,776	85.82	4,672	4,199
**KP433**	26,806,019	22,425,374	83.66	4,681	4,168
**KP434**	34,862,677	27,803,727	79.75	4,688	4,221
**KP435**	34,032,037	28,053,205	82.43	4,695	4,115
**KP436**	35,762,968	30,158,243	84.33	4,671	4,054
**KP437**	31,327,676	26,258,468	83.82	4,673	4,092
**KP438**	34,763,232	29,762,094	85.61	4,677	4,175
**KP439**	34,117,358	29,498,213	86.46	4,675	4,038

The RNA expression profiles are presented in Table 19. The table includes the numbers of reads used for alignment and the percentage of reads mapped to the reference. Additionally, it lists the numbers of genes with non-zero count values and expression values greater than 1 in the samples.

Table S2. Summary of differential expression gene analysis.

Ancestor	Strain	Filtered ID	UP	DOWN	Significantly differentiated genes (FDR <0.05)	
					UP	DOWN
	**KP111**	3,952	434	183	13	7
	**KP112**	4,034	70	52	19	4
	**KP113**	4,049	260	215	17	3
	**KP114**	4,035	81	107	16	4
	**KP115**	4,092	219	253	15	5
	**KP116**	4,053	203	121	18	2
	**KP117**	4,126	418	267	19	1
	**KP118**	4,091	425	393	12	8
	**KP119**	4,091	315	333	13	5
	**KP121**	3,986	424	177	12	8
	**KP122**	4,046	66	62	18	2
	**KP123**	4,101	75	66	18	2
	**KP124**	4,088	59	55	13	7
**KP1**	**KP125**	4,037	239	208	11	9
	**KP126**	3,947	48	71	0	20
	**KP127**	4,005	240	249	1	19
	**KP128**	4,051	302	294	0	20
	**KP129**	4,124	415	412	1	19
	**KP131**	4,004	478	179	17	3
	**KP132**	4,036	568	202	16	4
	**KP133**	4,056	416	183	17	3
	**KP134**	4,044	161	117	18	4
	**KP135**	4,017	173	113	17	0
	**KP136**	4,050	188	161	18	2
	**KP137**	4,020	213	113	17	3
	**KP138**	4,021	371	399	8	11
	**KP139**	4,031	360	228	18	2

	**KP411**	3,860	183	147	8	12
	**KP412**	3,899	237	94	9	11
	**KP413**	3,886	52	90	9	11
	**KP414**	3,936	268	374	12	8
	**KP415**	3,856	39	6	19	0
	**KP416**	3,942	187	284	15	5
	**KP417**	3,897	152	154	18	2
	**KP418**	3,932	252	293	17	3
	**KP419**	3,951	224	276	14	6
	**KP421**	3,751	96	61	4	16
	**KP422**	3,995	113	152	12	8
	**KP423**	3,856	166	45	15	2
	**KP424**	3,970	248	294	13	6
**KP4**	**KP425**	3,960	203	318	14	6
	**KP426**	3,925	194	226	17	3
	**KP427**	4,027	206	183	18	2
	**KP428**	3,908	461	564	8	12
	**KP429**	4,001	241	266	17	2
	**KP431**	3,853	226	136	10	10
	**KP432**	3,796	194	83	11	9
	**KP433**	4,047	187	239	13	7
	**KP434**	3,937	97	62	12	8
	**KP435**	3,959	174	331	10	10
	**KP436**	3,874	177	214	16	4
	**KP437**	3,986	267	415	9	11
	**KP438**	3,979	267	263	14	6
	**KP439**	3,925	293	375	16	4

The summary of the differential expression gene analysis is provided in Table 18. The "Filtered ID" column presents the number of samples with a read count greater than 2 in the ID (read count > 2) and ID ≥ 1. The "UP" and "DOWN" columns contain values that consist of FDR (adjusted p-value) < 0.05 and $\log_2$FoldChange ≥ 1.

Table S3. Significantly differentiated gene ontology numbers.

Ancestor	Strain	Days	Differentiated genes (Matched with database)	
			UP	DOWN
KP1	KP11	1	434 (166)	183 (88)
		9	315 (152)	333 (124)
	KP12	1	424 (172)	177 (100)
		9	415 (178)	412 (152)
	KP13	1	478 (176)	179 (98)
		9	360 (149)	228 (99)
KP4	KP41	1	183 (88)	147 (72)
		9	224 (110)	276 (122)
	KP42	1	96 (41)	61 (40)
		9	241 (114)	266 (115)
	KP43	1	226 (103)	136 (69)
		9	293 (129)	375 (148)

The numbers of genes exhibiting differential expression (fold change > 2, FDR < 0.05) and those mapped to the ontology database are presented. Significantly differentiated genes identified per strain and day are shown, with the number of genes annotated in the gene ontology database provided in parentheses.

Table S4. Brief summary of variated CDS positions of KP119, 129 and 139 revealed by whole-genome resequencing.

Position	Strain			Gene	Nucleotide change	Amino acid change	Mutation type
	119	129	139				
Chromosome	■			*phoP*	A187T	I63F	Point
		■		*basS*	A469C	T157P	Point
	■	■	■	*hp*	T80TC	E352A	Frame shift
	■	■	■	*ISVsa5*	G716GA	I241N	Frame shift
	■		■	*hp*	C405CA	N137K	Frame shift
	■	■	■	*hp*	A419AG	N141E	Frame shift
		■	■	*ISKpn28*	G10GT	Y149V	Frame shift
		■	■		C49CG		
	■	■	■	*ISVsa5*	G716GA	I241N	
		■		*mlaE*	CCGCGCTGCTGTTTGCCGGCCGCGCCGGGTCGG311C	L106D	Frame shift
			■	*ISVsa5*	C1161A	E17	Deletion
	■	■	■	*HP*	G966C	-	
	■	■	■	*IS4321R*	A18AG	W129L	Point
	■	■	■		T138G	N89H	
	■		■	*ISVsa5*	A80AT	I241N	Frame shift
	■		■	*hp*	A1051AG	E352G	Frame shift
	■	■	■	*ISKpn1*	C476T	W104	Deletion
	■	■	■	*ISKpn1*	T97C	-	
	■	■	■	*ISEsa2*	C10CA	P81A	Point
	■	■	■	*hp*	GC80G	A197G	Frame shift
	■	■	■	*ISKpn21*	G497GA	S168K	Point
	■	■	■	*glpR*	A220T	-	
	■	■	■		G490A		
	■	■	■	*ltnD*	C450A	-	
		■	■		T786C		
	■	■	■	*dgcP*	A742C	I248L	Point

pKP1_IncH1B_IncFIB	■	■	■	*folP*	G25GA	D173R	Frame shift
	■	■	■	*ISKpn21*	G497GA	S168K	Frame shift
	■	■	■	*IS903*	T503TC	E9G	Frame shift
	■	■	■	*hp*	CG327C	G60E	Frame shift
			■	*dinG*	C1356A	W167R	Point
					A1377T	D174Y	
	■			*hp*	C319CCGCTT	L159S	Frame shift
	■	■	■	*ISVsa5*	G716GA		Frame shift
	■	■	■	*IS4321R*	A265C	W129L	Frame shift
	■	■	■		T382TC		
		■		*IS4321R*	GA620AG	S69L	Point
	■	■	■	*ltnD*	GA249AG	R124W	Point
		■		*glpR*	CCA35C	T13L	Point
					C39CTG		
		■		*ISBcen27*	GT458AG	T316L	Point
		■		*hp*	A410C	K137T	Point
	■	■	■	*ISKpn28*	T27TC	T193D	Frame shift
pKP1_gtc3L95		■		*merA*	C591CG	W198V	Frame shift
	■	■	■	*hp*	TG33T	D13I	Frame shift
	■	■	■	*ISSen4*	T245TG	A83G	Point
pKP1_gtc5L70	■	■	■	*hp*	C555G	E16Q	Point
	■	■	■	*IS3000*	AG52A	P624L	Frame shift
	■	■	■	*hp*	C33G	-	
	■	■			C450G		
			■	*topB*	TC97T	-	
		■		*hp*	A6C	T2S	Frame shift
		■			C10CCG		
	■	■	■		T55C		
	■	■		*hp*	C55CT	K91E	Frame shift
		■		*virB4*	T15TC	V631G	

					G17A		Frame shift
					GA275AG		
				virB1	G21GTT	L166N	Frame shift
				IS3000	A40AT	N431K	Frame shift
				hp	C327G	-	
				topB	AC4AAACCA	G617V	Frame shift
				hp	TA98T	Y14I	Frame shift
				hp	C32CT	R225K	Frame shift
				hp	T26TA	Y65L	Frame shift
				virB4	CAT58C	M117G	Frame shift
pKP1_NDM5				*bla*	C412CAA	-	

As a result of the resequencing, various sites of variant genes were identified, including multiple frameshifts.

Table S5. Brief summary of variated CDS positions of KP419, 429 and 439 revealed by whole-genome resequencing.

Position	Strain			Gene	Nucleotide change	Amino acid change	Mutation type
	419	429	439				
Chromosome		■		*mlaF*	GCGCGC448G	A153P	Frame shift
	■	■	■	*ISVsa5*	G716GA	I241N	Frame shift
	■	■	■	*ISVsa5*	A80AT	I241N	Frame shift
	■	■	■	*hp*	C152CT	R271K	Frame shift
					T699G		
		■		*ISVsa5*	C1197A	D5Y	Point
	■	■	■	*ISKpn1*	C5CT	S159K	Point
	■			*rstB*	G872T	A64E	Point
	■	■	■	*hp*	C275CG	E93G	Frame shift
	■	■	■	*ISKpn1*	GAA36G	K15A	Frame shift
			■	*narZ*	A3465C	C93G	Point
	■			*catC*	G43GATGT	G15D	Frame shift
	■	■		*ISKpn21*	G497GA	S168K	Point
			■	*pspB*	TTT4GCG	K74R	Point
			■		C19T		
			■		G28A		
			■		G31A		
			■		C34A		
	■	■	■	*pspA*	C565T	-	
	■	■	■		GA579AC		
	■	■	■		T592C		
			■		G598A		
	■			*hp*	T790G	-	
	■	■	■	*hp*	T80TC	E352G	Frame shift
	■				T481G		
	■	■	■	*dgcP*	A742C	-	Point
	■	■		*ISKpn1*	ATT26A	K169N	Frame shift
	■	■	■	*ISVsa5*	G716GA		

				hp	ACA1972CTG	C93E	Frame shift
				hp	T60TC	I276D	Frame shift
				hp	AG8A	L108 W	Point
				hp	CG21C	H275T	Frame shift
				hp	C14CT	R175K	Frame shift
				hp	C649CA	A267G	Frame shift
				hp	G16GGA	F54P	Frame shift
				hp	A39AG	W93L	Frame shift
				hp	C43CT	R115E	Frame shift
				hp	GT95AG	T77L	Point
				hp	T200TA	-	
				hp	G890A	P18L	Point
				hp	C5CCAGGG	G40A	Frame shift
				ISVsa5	C1161A	E17	Deletion
				ISVsa5	G716GA	I241N	
				hp	T2247TCC	H751L	Frame shift
				hp	C405CA<br>AAA408ACACAC<br>A418AG	N137T	Frame shift
				wecB	C870G	D73H	Point
pKP4_IncH1B_IncFIB				*ISVsa5*	G49T	E17	Deletion
				xerC	G115GC	C227 W	Frame shift
				IS4321R	CC123CTG<br>T699G	G281R	Frame shift
				hp	AC301CT<br>T429G	R174Q	Point
				ISVsa5	T589TG	197W	Addition
				ISKpn28	A460AGG<br>T477TA<br>C632T	P212L	Point

	■	■	■	*hp*	T80TC	E352G	Frame shift
	■	■	■	*ISVsa5*	G716GA		
	■	■	■	*ISVsa5*	A80AT		
	■	■	■	*IS903*	C417CCAG	140Q	Addition
	■	■	■	*ISBcen27*	T398TC	L134P	Frame shift
	■			*hp*	A557C	D168A	Point
	■				A570C	L191R	
	■				T572G		
	■	■		*ISRaq1*	AC244CT	T82L	Point
	■			*IS4321R*	T741G	N89H	Point
pKP4_gtc5L70	■	■	■	*bla_{TEM-12}*	TC19T	G155D	Frame shift
pKP4_gtc3L95	■	■		*ISEsa2*	CG502CTGGA	R168LE	Point
	■			*IS26*	T262A	W88R	Point
	■	■	■	*hp*	T80TC	E351G	Frame shift
pKP4_NDM5		■	■	*IS903*	C20CA	V1R	Frame shift
	■				AA24ACAC		
	■	■			A33AG		
			■		A35G		

As a result of the resequencing, various sites of variant genes were identified, including multiple frameshifts.

Table S6. Primers used for qRT-PCR.

Genes	Forward	Reverse
lsrB	CGTTTGCTGAAAAAGGGCGA	CTCTTTGCTGAACACCACGC
lsrD	ATTTAACCCTCGCCGGACTG	GCAGATACCCCACCAGCAAT
lsrG	CAACCTGCGCTTTGATGTCC	CTGCGGTAACAGGCCGATAA
arnA	CAGACCCTGCACAATCTGGT	CATCCAGTTGAACGGACGGA
arnC	TGAAGGTTACGACGTGGTGG	AGGCCCATACCGACAAACTG
entB	AACAGCGCGATGATGGAGAA	AACAGCGCGATGATGGAGAA
16S rRNA	TTCCACCTGCACCCTGAATC	TCTTTATCCCGCTGCCACAG
